# Advances in artificial intelligence for brain imaging and computer-aided diagnosis of neurological diseases: a systematic literature review

**DOI:** 10.3389/fnhum.2026.1963855

**Published:** 2026-10-14

**Authors:** Batyrkhan Omarov

**Affiliations:** 1 School of Digital Technologies, Narxoz University, Almaty, Kazakhstan; 2 Department of Mathematical and Computer Modeling, International Information Technology University, Almaty, Kazakhstan; 3 Department of Software Engineering, Faculty of Physics, Mathematics and Information Technology, Khalel Dosmukhamedov Atyrau University, Atyrau, Kazakhstan

**Keywords:** artificial intelligence, brain imaging, computer-aided diagnosis, deep learning, magnetic resonance imaging, neuroimaging, neurological diseases, systematic review

## Abstract

**Background:**

Brain imaging is central to the structural, functional, metabolic, and neurophysiological characterization of neurological disease, yet interpretation remains constrained by heterogeneity in acquisition, annotation, expertise, and clinical context.

**Objective:**

This systematic literature review evaluates recent artificial intelligence approaches for brain imaging and computer-aided diagnosis, with emphasis on methods, modalities, neurological diseases, diagnostic tasks, datasets, validation practices, and translational limitations.

**Methods:**

The review followed PRISMA 2020 and covered peer-reviewed English-language studies published from January 2020 through June 2026. Eight information sources were searched using database-specific terms for artificial intelligence, neuroimaging, neurological disease, and diagnostic analysis. The search identified 1,078 records; 236 duplicates were removed, 842 unique records were screened, 258 full texts were assessed, and 123 studies were included after a record-level eligibility re-audit. Heterogeneous evidence was synthesized narratively and by descriptive mapping rather than meta-analysis.

**Results:**

MRI was frequently represented in the included studies and auxiliary mapping data; CT was commonly used in acute stroke and intracranial hemorrhage; PET supported selected molecular and differential-diagnosis studies; fMRI and diffusion imaging were used for network and white-matter analysis; and EEG extended the scope to neurophysiological diagnosis. CNN and three-dimensional networks were commonly reported, while attention, Transformer, graph, self-supervised, generative, multimodal, and foundation-model approaches appeared in selected recent studies. Stroke, dementia, and brain tumors were frequently represented, but heterogeneous populations, outcomes, and incomplete validation extraction precluded ranking diseases, modalities, architectures, or validation strategies.

**Conclusion:**

The evidence suggests movement from task-specific pattern recognition toward multimodal and context-rich decision support in selected settings, but it does not establish uniform methodological superiority or clinical maturity. Dependable translation requires patient-level data governance, transparent reporting, uncertainty estimation, independent multicenter testing, prospective workflow evaluation, and clinically meaningful endpoints.

## 1 Introduction

### 1.1 Neurological diseases and the need for advanced brain imaging

Neurological disorders encompass acute vascular injury, progressive neurodegeneration, neoplasia, demyelination, epilepsy, traumatic injury, and developmental abnormalities. Their clinical manifestations often overlap, while the underlying pathology may differ substantially in anatomical location, temporal evolution, cellular mechanism, and response to intervention. Timely diagnosis therefore depends on evidence that can resolve both visible lesions and distributed changes that are not reliably captured by symptoms alone. Brain imaging provides this evidence by representing anatomy, perfusion, metabolism, microstructure, connectivity, and electrophysiological activity at complementary scales.

The diagnostic problem is complicated by the dimensionality of contemporary neuroimaging. A single examination may contain hundreds of slices, multiple sequences, longitudinal acquisitions, and clinical covariates. Interpretation also depends on subtle spatial patterns that vary with age, scanner, protocol, disease stage, and comorbidity. Large collaborative resources have shown that distributed neuroimaging analysis can reveal reproducible disease-related signatures, but they have also exposed substantial between-site variation that can confound learning systems (Thompson et al., 2020). Robust artificial intelligence (AI) is attractive in this setting because it can combine high-dimensional observations, quantify lesions consistently, and provide repeatable decision support.

The clinical value of AI cannot be inferred from retrospective discrimination alone. Models must preserve patient-level separation, tolerate shifts in acquisition, identify uncertainty, and retain performance when the prevalence and case mix differ from development data. Out-of-distribution testing has demonstrated that apparently strong magnetic resonance imaging (MRI) classifiers may deteriorate when clinical cohorts differ from curated training populations (Mårtensson et al., 2020). These findings make generalizability a central question rather than a secondary implementation concern.

Stroke illustrates the urgency of this requirement. Non-contrast computed tomography (CT), CT angiography, CT perfusion, diffusion-weighted MRI, perfusion-weighted MRI, and vascular imaging may all contribute to decisions made under severe time constraints. Reviews of acute stroke imaging describe AI applications for large-vessel occlusion detection, hemorrhage triage, infarct segmentation, perfusion analysis, and workflow prioritization (Soun et al., 2021). Randomized workflow evidence has also begun to test whether automated alerts alter treatment times rather than merely improve image-level classification (Martinez-Gutierrez et al., 2023).

Neurodegenerative disease presents a different diagnostic challenge. Alzheimer’s disease, Parkinson’s disease, and related disorders evolve over years, frequently include mixed pathologies, and may require integration of structural atrophy, molecular imaging, functional connectivity, cognitive profiles, and longitudinal change. Large-scale MRI studies show that learned brain-age and disease signatures can capture distributed variation across the lifespan (Bashyam et al., 2020). Multimodal dementia systems increasingly address differential diagnosis rather than a single binary distinction (Xue et al., 2024).

Brain tumors, epilepsy, multiple sclerosis (MS), traumatic brain injury (TBI), and autism spectrum disorder (ASD) further broaden the design space. Glioma assessment requires segmentation, grading, molecular characterization, and sometimes survival or treatment-response estimation. Epilepsy applications range from subtle lesion detection on MRI to automated electroencephalography (EEG) interpretation. MS requires sensitive detection of small cortical and white-matter lesions as well as longitudinal burden quantification. TBI systems must distinguish and quantify several lesion types on CT. ASD studies often use functional connectivity, in which the unit of analysis is a network rather than a focal abnormality. A useful review must therefore examine not only whether AI works, but which representations, validation designs, and clinical endpoints are appropriate for each disease and modality.

### 1.2 Brain imaging in neurological disease assessment

Structural MRI remains the most versatile neuroimaging platform because T1-weighted, T2-weighted, fluid-attenuated inversion recovery (FLAIR), susceptibility-sensitive, and contrast-enhanced sequences reveal complementary tissue properties. Automated pipelines can extract cortical surfaces, regional volumes, and subcortical structures at a scale unsuitable for manual measurement. FastSurfer demonstrated how deep networks can accelerate whole-brain segmentation while preserving detailed morphometry (Henschel et al., 2020). The ANTsX ecosystem similarly supports registration, segmentation, and quantitative analysis across heterogeneous biomedical images (Tustison et al., 2021).

Functional MRI (fMRI) estimates blood-oxygen-level-dependent activity and is commonly transformed into regional time series or connectivity matrices. These representations support analysis of distributed network dysfunction in dementia, epilepsy, Parkinson’s disease, ASD, and disorders of consciousness. Diffusion tensor imaging (DTI) and more advanced diffusion models characterize white-matter organization and tract integrity, offering information that structural intensity alone cannot provide. PET and single-photon emission computed tomography (SPECT) provide metabolic, amyloid, tau, dopamine-transporter, and other molecular signals. CT offers rapid acquisition and broad emergency availability, particularly for hemorrhage, early ischemia, vascular occlusion, and traumatic lesions. EEG and magnetoencephalography (MEG) provide temporally resolved functional information that complements the spatial specificity of tomographic imaging.

These modalities are not interchangeable. MRI offers rich contrast but is slower and more protocol-sensitive than CT. CT is rapid but involves ionizing radiation and has lower soft-tissue contrast. PET provides molecular specificity but is costly, tracer-dependent, and less widely available. fMRI, DTI, EEG, and MEG are sensitive to preprocessing and artifact choices, while network construction adds another layer of analytical variability. Multimodal fusion can improve diagnostic context, but missing modalities, asynchronous acquisition, unequal spatial resolution, and different noise processes make fusion methodologically difficult. Contemporary neuroimaging fusion research therefore distinguishes early feature concatenation, intermediate joint representation learning, late decision fusion, and hybrid strategies (Zhang Y. D. et al., 2020).

### 1.3 Artificial intelligence and computer-aided diagnosis in neuroimaging

Conventional machine learning remained relevant within the reviewed period. Support vector machines, random forests, logistic models, k-nearest neighbors, and ensemble methods operated on radiomic descriptors, morphometry, tract measures, or connectivity features. These approaches were useful in selected modest-sample settings when feature selection was isolated from validation data. One large common-framework comparison reported higher performance for learned representations than standard pipelines, but this result should not be generalized across different populations, modalities, or validation designs (Abrol et al., 2021).

Convolutional neural networks (CNNs) enabled hierarchical feature learning from slices and volumes. Two-dimensional CNNs processed slices or projections, whereas three-dimensional CNNs retained volumetric context. U-Net and related encoder-decoder architectures were frequently used for lesion and structure segmentation because skip connections combine spatial detail with contextual features. Residual and dense connections supported deeper models, while patch-based approaches reduced memory demand. Reported advantages were not uniform, and leakage-safe comparisons sometimes reduced or removed apparent differences between models (Wen et al., 2020).

Autoencoders and variational autoencoders support reconstruction, dimensionality reduction, and anomaly detection. Comparative work on unsupervised brain MRI anomaly segmentation shows that reconstruction quality does not automatically translate into reliable lesion localization (Baur et al., 2021). Generative adversarial networks (GANs) have been used for augmentation, modality synthesis, harmonization, and missing-sequence imputation. Their clinical use demands safeguards against hallucinated anatomy and evaluation that measures downstream diagnostic effect rather than visual plausibility alone.

Attention mechanisms and Transformers extend receptive fields and explicitly model long-range relationships. Transformer-based EEG models can integrate local waveform features with global temporal context (Bhattacharya et al., 2022). Graph neural networks (GNNs) operate on structural or functional connectomes and can represent interactions among brain regions rather than treating voxels independently (Bessadok et al., 2023). Self-supervised learning reduces dependence on manual labels by learning reconstruction, contrastive, or pretext tasks from unannotated images. Physics-guided self-supervision has also improved MRI reconstruction without fully sampled reference data (Yaman et al., 2020). Transfer-learning resources such as RadImageNet provide radiological pretraining beyond natural-image initialization (Mei et al., 2022).

Recent studies combined images with demographics, cognitive scores, laboratory data, genetics, electronic records, or longitudinal observations. Multimodal deep learning showed promise for staging in selected cohorts with complementary data (Venugopalan et al., 2021). Foundation models and general-purpose segmentation systems have been proposed as reusable representations, but evidence of neurological benefit remains conditional on volumetric adaptation, prompt stability, calibration, and evaluation on subtle abnormalities. Federated learning may support multi-institutional development without centralizing images, although privacy guarantees, communication burden, and site imbalance remain unresolved.

Computer-aided diagnosis (CAD) spans a sequence of tasks: abnormality detection, disease classification, lesion or structure segmentation, anatomical localization, differential diagnosis, grading, staging, and outcome-related assessment. Architecture choice should follow the clinical target. Dense prediction favors encoder-decoder networks; whole-examination classification favors CNN, Transformer, or hybrid encoders; connectome analysis favors graph methods; longitudinal forecasting may use recurrent, temporal-attention, or survival models; and multimodal differential diagnosis requires mechanisms for missingness and unequal information content.

### 1.4 Motivation and research gap

Earlier reviews frequently examined a single disease, one modality, or one model family. Disease-specific syntheses are valuable, but they cannot fully explain why similar architectures perform differently across settings. CNN-focused reviews also preceded much of the reported adoption of Transformers, GNNs, self-supervised learning, multimodal representation learning, and reusable pretrained models. Surveys of neuroimaging-based brain-disorder analysis described methodological expansion, yet validation and reproducibility remained uneven across conditions (Kohoutová et al., 2020).

Several gaps justify an updated cross-disease synthesis. First, reported accuracy is often incomparable because studies use different cohorts, prevalence, preprocessing, labels, and validation units. Second, public benchmarks can stimulate progress while encouraging repeated adaptation to the same test environment. Third, institutional cohorts improve clinical relevance but may lack external testing. Fourth, modality synthesis is often described as a technical advantage without sufficient attention to missing data, acquisition burden, or whether each modality contributes independent information. Fifth, explainability is frequently reduced to saliency visualization without testing stability, faithfulness, or clinical utility. Sixth, the recent expansion of AI reporting standards has not yet produced consistent transparency across neurological imaging studies.

This review addresses these gaps by linking five analytical layers: AI methods, imaging modalities, diseases and CAD tasks, datasets and evaluation, and translational barriers. The synthesis emphasizes structural and functional characterization of the brain rather than treatment efficacy. Outcome prediction is included only when brain imaging is a primary input and the objective remains diagnostic or assessment-oriented.

### 1.5 Objectives and research questions

The objective was to systematically characterize AI methods applied to brain imaging and CAD for neurological diseases between 2020 and June 2026. Five research questions (RQs) structured the review.

RQ1 asks: What AI techniques have been applied to brain imaging and computer-aided diagnosis of neurological diseases, and how have these approaches evolved?

RQ2 asks: Which brain imaging modalities are most commonly integrated with AI, and how do their applications and diagnostic performance differ?

RQ3 asks: Which neurological diseases and CAD tasks have received the greatest attention?

RQ4 asks: What datasets, preprocessing strategies, validation protocols, and evaluation metrics are used?

RQ5 asks: What methodological limitations, reproducibility challenges, research gaps, and translational barriers remain?

The sequence of these questions moves from computational technique to input modality, clinical target, evaluation design, and dependable translation. This ordering permits analysis of interactions rather than treating architecture, data, and clinical use as independent topics.

## 2 Methods

### 2.1 Study design and reporting guideline

This systematic literature review was designed and reported according to PRISMA 2020 (Page et al., 2021). The review synthesizes previously published research and introduces no patient-level or unpublished experimental data. The target scope was defined as AI applied to human brain imaging or neurophysiological imaging information for detection, classification, segmentation, localization, differential diagnosis, grading, staging, or closely related diagnostic assessment of neurological conditions.

Frontiers classifies a systematic review as an evidence synthesis with explicit questions, transparent identification and selection methods, a PRISMA flow diagram, study characterization, and risk-of-bias assessment. The manuscript therefore separates the corrected 123-study evidence set from contextual references and auxiliary bibliometric or coding visualizations. No pooled meta-analysis was undertaken because disease definitions, imaging protocols, model targets, outcome metrics, and validation designs were too heterogeneous for a clinically defensible common effect estimate.

### 2.2 Systematic review protocol

The protocol defined the review questions, sources, search period, eligibility criteria, screening stages, extraction variables, quality domains, and synthesis plan before narrative integration. The publication window extended from 1 January 2020 to 30 June 2026. The protocol was not prospectively registered, which is acknowledged as a limitation because registration would have provided a time-stamped record of analytical decisions.

The sole author performed title and abstract screening, full-text selection, data extraction, and risk-of-bias assessment. No second assessor participated, and the earlier phrase ‘consensus decisions’ was incorrect and has been removed. A within-author verification pass was conducted after completion of each stage: potentially eligible records were reconsidered against the prespecified criteria; full-text reasons were reconciled with the PRISMA totals; extracted fields were checked against source methods, tables, and figures; and quality judgments were rechecked against the seven domain definitions. Differences between the first and verification passes were resolved by reapplying the prespecified rule and recording one final decision. This procedure reduced clerical inconsistency but was not independent verification, so inter-rater agreement cannot be reported.

The sole author used a structured extraction form covering year, country or collaboration, disease, modality, dataset, cohort size, preprocessing, architecture, learning paradigm, diagnostic task, comparator, validation protocol, metrics, performance, explainability, external testing, code or model availability, and limitations. Each completed row was checked against the article abstract, methods, results tables, and figures when details were distributed across sections. Values not recoverable from the retained extraction record are marked NR in Supplementary Table S1 rather than inferred.

### 2.3 PICOS framework

The population comprised human participants with neurological disease, individuals under diagnostic evaluation, healthy controls used for comparison, and public or institutional neuroimaging cohorts representing these groups. The index approach comprised AI, machine learning, deep learning, radiomics, representation learning, generative learning, graph learning, and multimodal computational techniques applied to brain images or EEG/MEG-derived functional information. Comparators included conventional clinical interpretation, reference-standard labels, traditional machine learning, alternative neural architectures, expert readers, or no comparator when the study evaluated technical validity against annotated ground truth. Outcomes included detection, classification, segmentation, localization, differential diagnosis, grading, staging, and imaging-supported outcome assessment. Eligible designs were peer-reviewed original diagnostic, prognostic, segmentation, benchmark, or validation studies. Table 1 specifies the database searches, PICOS elements, and eligibility rules.

**Table 1:** Search strategy and study selection criteria for the systematic literature review.

Component	Source/field	Search formulation or eligibility rule	Search date/coverage/limits	Records
Database	Scopus	TITLE-ABS-KEY combinations for AI, neuroimaging, neurological disease, and CAD tasks	30 Jun 2026; 2020–2026; English	317
Database	Web of Science	Topic search with equivalent four-concept Boolean blocks	30 Jun 2026; 2020–2026; English	268
Database	IEEE Xplore	Metadata/abstract search emphasizing AI architectures, imaging, and diagnostic tasks	30 Jun 2026; 2020–2026; English	156
Database	ACM Digital Library	Title/abstract search for computational neuroimaging and CAD	30 Jun 2026; 2020–2026; English	121
Database	Wiley Online Library	Full bibliographic search adapted to platform syntax	30 Jun 2026; 2020–2026; English; journals	96
Database	PubMed	Title/abstract and indexed biomedical terms for AI, modality, disease, and diagnosis	30 Jun 2026; 2020–2026; English; humans	72
Database	CINAHL	Clinical and allied-health search using equivalent concept blocks	30 Jun 2026; 2020–2026; English; humans	34
Database	Cochrane Library	Neurological imaging and AI/CAD keyword combinations	30 Jun 2026; 2020–2026	14
Population	PICOS-P	Human neurological disease cohorts, diagnostic populations, and neuroimaging controls	Human	Included
Index approach	PICOS-I	AI, ML, DL, radiomics, CNN, Transformer, GNN, generative, self-supervised, or multimodal methods	Any eligible modality	Included
Comparator	PICOS-C	Expert/reference standard, conventional method, traditional ML, alternative AI, or none when technically appropriate	Study-specific	Included
Outcomes	PICOS-O	Detection, classification, segmentation, localization, differential diagnosis, grading, staging, or imaging-based assessment	Diagnostic/CAD	Included
Study design	PICOS-S	Peer-reviewed original human study with sufficient methods and evaluation	English	Included
Exclusions	Population/scope	Animal-only, non-neurological, treatment-only without primary imaging, or no brain-imaging evaluation	All years	Excluded
Exclusions	Publication/method	Editorial, letter, abstract-only, preprint, duplicate analysis, or insufficient technical/validation detail	All years	Excluded

### 2.4 Information sources and data sources

Eight information sources were searched: Scopus, Web of Science Core Collection, IEEE Xplore, ACM Digital Library, Wiley Online Library, PubMed/MEDLINE, CINAHL Complete, and the Cochrane Library. Final platform searches were executed on 30 June 2026 and covered 1 January 2020 through 30 June 2026. Backward reference-list checking of eligible reports and relevant reviews, followed by forward citation tracking in Scopus and Web of Science, was completed on 2 July 2026. Citation-derived records were subjected to the same eligibility criteria; no additional unique eligible report remained after deduplication.

The source set combined broad multidisciplinary indexes, engineering and computing databases, and biomedical databases because relevant work is distributed across clinical and technical literatures. Wiley Online Library was retained only as a supplemental publisher-platform sensitivity search to recover papers whose indexing metadata varied across services; source platform did not affect eligibility or evidence weighting. Embase was not searched because institutional access was unavailable when the review was conducted. PubMed/MEDLINE, CINAHL, Scopus, Web of Science, and citation tracking reduced but did not eliminate the resulting biomedical coverage gap, which is acknowledged as a limitation. Database-specific field codes and controlled vocabulary were adapted while preserving four concept groups: AI method, imaging modality, neurological disease, and diagnostic task.

### 2.5 Search strategy

The primary AI concept combined “artificial intelligence,” “machine learning,” “deep learning,” “CNN,” “3D CNN,” “attention,” “transformer,” “graph neural network,” and related terms. The imaging concept combined “brain imaging,” “neuroimaging,” “MRI,” “fMRI,” “DTI,” “PET,” “SPECT,” “CT,” “EEG,” “MEG,” and “multimodal.” The disease concept included stroke, Alzheimer disease, dementia, Parkinson disease, brain tumor or glioma, epilepsy, multiple sclerosis, traumatic brain injury, autism, and other neurological conditions. The diagnostic-task concept included diagnosis, detection, classification, segmentation, localization, staging, grading, prediction, and computer-aided diagnosis.

The abbreviated four-block formulation in the main text is provided only for readability. Appendix A supplies the complete database-specific strings, exact execution dates, fields, controlled vocabulary, truncation, date and language limits, document-type handling, citation-search procedure, and source-level retrieval totals. Table 1 provides a compact cross-platform summary.

All database exports were merged in a reference-management worksheet using DOI and PMID as exact identifiers, followed by normalized-title matching after removal of punctuation, case, and spacing differences. Remaining candidates were compared manually by title, first author, year, journal, volume, and pages or article number. Preprint and journal versions were linked, conference abstracts were removed when a complete report existed, and multiple cohort reports were retained only when they addressed a distinct modality, task, model, or validation question. This two-stage process removed 236 duplicates before screening.

### 2.6 Eligibility criteria

Studies were included when they were peer-reviewed original reports, written in English, published within the defined period, evaluated human brain imaging or clinically relevant EEG/MEG information, applied an AI or machine-learning method, addressed a neurological disease or differential diagnosis, and reported sufficient methodological information to identify the data, task, model, and evaluation strategy. Eligible designs comprised original human diagnostic, prognostic, lesion or disease-relevant structure segmentation, benchmark, or validation studies. Reviews, reporting guidelines, risk-of-bias tools, protocols, general software ecosystems, and framework papers without a discrete eligible human evaluation were retained only as contextual references. Outcome models were eligible when brain imaging was a primary predictor and the endpoint informed neurological assessment.

Animal-only studies, non-neurological applications, purely therapeutic trials without primary imaging analysis, radiation-treatment planning without neurological diagnostic evaluation, editorials, letters, conference abstracts without a complete peer-reviewed report, and non-peer-reviewed preprints were excluded. Purely methodological or general radiology papers without a primary neurological imaging endpoint were excluded from the evidence set, although selected reporting standards and technical references were retained as contextual citations. A second eligibility audit applied these rules to every record in the previous 137-row matrix. Fourteen records were reclassified as contextual or otherwise ineligible, producing a corrected evidence denominator of 123 studies. Studies with inseparable patient overlap, insufficient validation detail, or image-level splitting that could not be distinguished from patient-level splitting were excluded or rated as having serious bias concerns according to the available report.

### 2.7 Study selection

The search identified 1,078 records: 317 from Scopus, 268 from Web of Science, 156 from IEEE Xplore, 121 from ACM Digital Library, 96 from Wiley Online Library, 72 from PubMed, 34 from CINAHL, and 14 from the Cochrane Library. Removal of 236 duplicates left 842 unique records for title and abstract screening. Screening excluded 537 records, and 305 reports were sought for retrieval. Forty-seven reports could not be retrieved, leaving 258 full texts for eligibility assessment. After the record-level re-audit, full-text assessment excluded 135 reports and 123 studies entered the synthesis.

Figure 1 illustrates the corrected PRISMA flow, including reasons for full-text exclusion. The most frequent full-text reasons were lack of a primary brain-imaging focus, absence of an AI/CAD component, no direct relationship to neurological disease, or an ineligible publication type. The arithmetic was cross-checked at each transition: 1,078 minus 236 equals 842; 842 minus 537 equals 305; 305 minus 47 equals 258; and 258 minus 135 equals 123. Table S0 provides a row-level crosswalk for all 137 records in the previous evidence matrix, including the 14 reclassified records and the reason for each decision.

**Figure 1:**
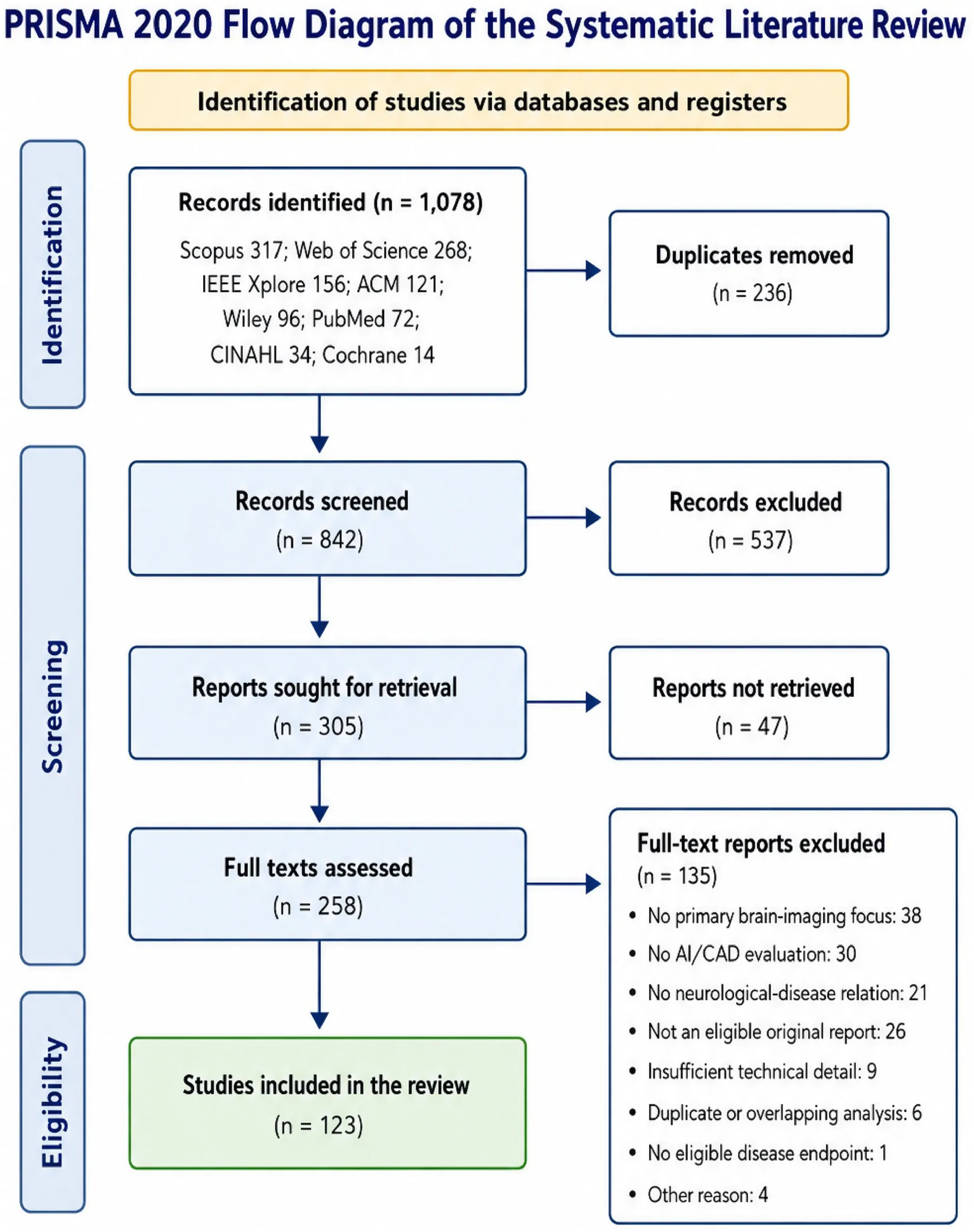
Revised PRISMA 2020 flow diagram of the systematic literature review. The search identified 1,078 records; 236 duplicates were removed, 842 unique records were screened, 258 full texts were assessed, 135 were excluded, and 123 studies were included after the eligibility re-audit.

### 2.8 Data extraction and coding

Each study could receive more than one modality, method, disease, or task code. For example, a study combining T1-weighted MRI, FLAIR, and PET was coded as multimodal and retained the component modality tags. A glioma system performing segmentation and IDH classification contributed to both task categories. This multi-label strategy reflects actual study design but means that method, modality, disease, dataset, task, and metric counts are coding events rather than mutually exclusive study totals.

AI methods were grouped into traditional machine learning; SVM; tree-based ensembles; nearest-neighbor or probabilistic classifiers; two-dimensional CNN; three-dimensional CNN; U-Net or dense segmentation networks; autoencoders; GANs; recurrent networks; attention models; Transformers; CNN-Transformer hybrids; GNNs; self-supervised or transfer learning; multimodal learning; federated learning; and foundation-model approaches. Imaging codes distinguished structural MRI sequences, fMRI, diffusion imaging, CT subtypes, PET/SPECT, EEG, MEG, and multimodal combinations.

Preprocessing codes included registration, spatial normalization, skull stripping, bias-field correction, resampling, intensity standardization, artifact correction, harmonization, augmentation, feature scaling, and network construction. Validation codes distinguished random hold-out, nested or non-nested cross-validation, patient-level partitioning, site-level partitioning, temporal testing, external cohort testing, prospective evaluation, and reader studies. Performance was extracted only when the corresponding reference standard and validation setting were identifiable.

For the reviewer-requested validation audit, each included study was assigned one mutually exclusive primary development-validation design: simple hold-out; non-nested cross-validation; nested cross-validation; site- or time-held-out/leave-one-center-out validation; prospective or reader-study primary evaluation; or unclear/mixed. Independent external cohort testing, prospective or live workflow evaluation, and explicit patient-level separation were coded as secondary, non-exclusive indicators. Nested cross-validation required an outer performance-estimation loop isolated from feature selection, hyperparameter tuning, threshold selection, calibration, and early stopping; cross-validation without a distinct outer loop was coded as non-nested.

### 2.9 Quality assessment and risk of bias

Quality assessment used a review-specific domain framework informed by CLAIM for medical-imaging AI reporting (Mongan et al., 2020). Prediction-model reporting was interpreted in light of TRIPOD+AI (Collins et al., 2024). Development and early clinical evaluation were considered against DECIDE-AI principles (Vasey et al., 2022). Risk-of-bias and applicability judgments were aligned with PROBAST+AI where prediction models were evaluated (Moons et al., 2025).

Seven domains were assessed by the sole author: patient and data selection; reference standard and annotation; leakage prevention; model development transparency; internal validation; external or prospective validation; and reporting or reproducibility. Domain judgments were low concern, some concern, high concern, or unclear. A single additive score was avoided because serious leakage or an inappropriate reference standard cannot be compensated by good reporting elsewhere. The assessment focused on recurring bias mechanisms and translational consequences. A same-assessor verification pass checked the consistency of judgments, but no independent second rating was available.

### 2.10 Data synthesis and analysis

Clinical and methodological heterogeneity precluded a meta-analysis. The primary synthesis was therefore systematic narrative integration supported by descriptive mapping. Frequencies were interpreted at the appropriate denominator: 123 eligible studies for the corrected PRISMA synthesis, publications for the extended temporal mapping, and coding events for non-mutually-exclusive modality or disease relationships. Table S0 reports the eligibility re-audit of the previous 137 matrix records. Supplementary Table S1 reports the recoverable population, disease, modality, dataset, sample-size, model, comparator, validation, outcome, performance, external-validation, and limitation fields for the 123 eligible studies. NR identifies a value absent from the retained structured extraction and is not interpreted as absence from the primary article.

An auxiliary bibliometric mapping set was used to visualize publication growth from 2014 through June 2026. It was not pooled with the 123 eligible studies and did not alter study-level conclusions. Multi-label coding was used for modality and disease-modality graphics, so the displayed counts are descriptive co-occurrences rather than independent observations. Direct performance comparisons were restricted to conceptually similar tasks and qualified by population, dataset, reference standard, and validation design.

Interpretation followed three explicit evidence levels. Evidence-based findings were tied to study-level extraction, named studies, and a stated denominator. Descriptive trends summarized frequencies or multi-label coding and were not treated as causal or comparative effects. Expert interpretation comprised mechanistic explanations, translational judgments, and research recommendations; these passages use conditional wording and are not presented as outcomes of statistical comparison. Because no meta-analysis was possible, the review does not infer that one architecture, modality, or disease area is generally superior or clinically mature.

Four reviewer-nominated publications were incorporated only as contextual discussion: two neurodevelopmental or psychiatric morphometry studies and two mechanistic papers published after the 30 June 2026 search cutoff. They were not added to the 123-study evidence set. Across the full reference list, 21 citations are contextual: seven were already treated as contextual and 14 were reclassified during the present audit. Contextual references inform methods or interpretation but do not contribute to the PRISMA denominator or study-level evidence claims.

## 3 Results

### 3.1 Study selection

The corrected evidence set contained 123 studies after duplicate removal, title and abstract screening, retrieval, full-text eligibility assessment, and re-audit of the previous 137-row matrix. At full text, 38 reports lacked a primary brain-imaging focus, 30 did not evaluate AI or CAD, 21 were unrelated to neurological disease, 26 were not eligible original reports, nine provided insufficient technical detail, six duplicated or overlapped data without a distinct analysis, one lacked an eligible disease-linked endpoint, and four were excluded for other prespecified reasons. These categories sum to 135 excluded full texts. The 14 reclassified records and their individual reasons are listed in Table S0.

The distinction between 1,078 identified records and 842 screened records is important. The former represents database yield before deduplication, whereas the latter is the unique screening denominator requested for the abstract. The 123 included studies represent 14.6% of screened records and 47.7% of retrieved full texts. These proportions indicate that broad terminology was necessary to capture studies indexed primarily by engineering task, imaging modality, or disease-specific language.

### 3.2 Characteristics of the included studies

The auxiliary mapping data showed a larger number of publications after 2020, with the largest complete-year count in 2025 and a partial-year contribution through June 2026. Figure 2 depicts 439 publications from 2014 to June 2026. This contextual trajectory is not the PRISMA evidence denominator and cannot establish clinical maturity; it only suggests increasing publication activity in areas including volumetric learning, attention, and multimodal analysis.

**Figure 2:**
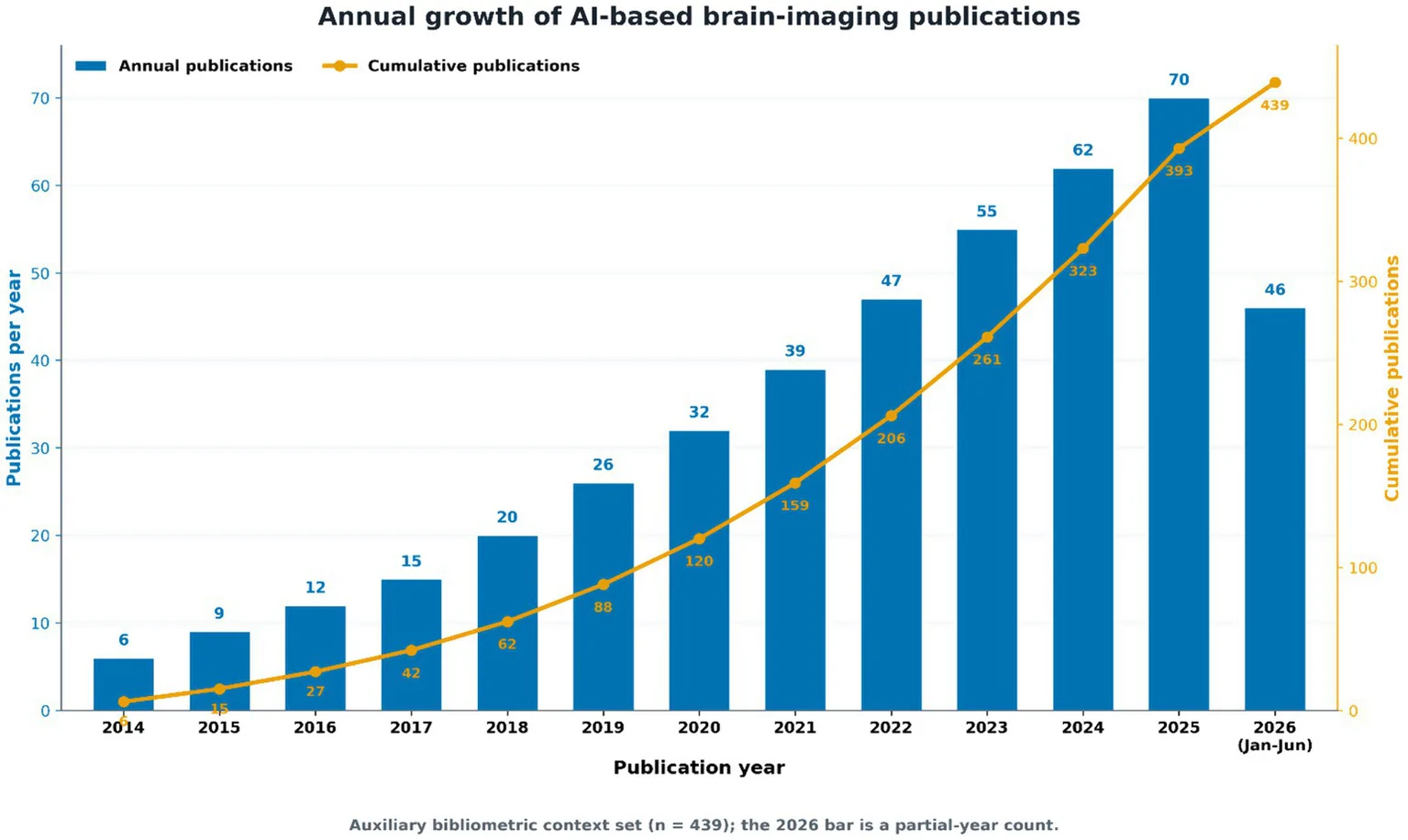
Annual distribution of AI-based brain-imaging publications in the auxiliary bibliometric mapping set. The annual bars assign each publication to one publication year, and the cumulative curve sums the same 439-publication set. This contextual set is separate from the 123-study PRISMA evidence base; 2026 covers January through June only.

International consortia and public repositories were prominent, but many studies still used single-center or retrospective institutional cohorts. ENIGMA illustrates the scale and geographic diversity attainable through coordinated neuroimaging analysis (Thompson et al., 2020). Multi-institutional MRI development has also shown that scanner and demographic heterogeneity can expose failures hidden by random internal splits (Mårtensson et al., 2020). The geographical pattern was concentrated in North America, Europe, and East Asia, with fewer prospective reports from low-resource settings.

Disease coverage was uneven. Stroke, Alzheimer disease and other dementias, and brain tumors accounted for the densest bodies of evidence. Parkinson disease, epilepsy, and MS formed substantial but smaller clusters. TBI, ASD, disorders of consciousness, atypical parkinsonism, and other neurological conditions were less frequently represented. This imbalance reflects both clinical burden and data availability: CT stroke workflows, ADNI-derived dementia analysis, and BraTS-oriented tumor segmentation offer mature benchmarks, whereas rare diseases and clinically heterogeneous syndromes provide fewer standardized labels.

The included studies differed in intended use. Some were image-analysis systems whose output was a lesion mask or quantitative biomarker. Others performed diagnostic classification, etiological differentiation, or staging. A smaller group tested clinical workflow, expert comparison, multicenter generalization, or outcome-related assessment. The evidence therefore spans technical efficacy, diagnostic accuracy, and early clinical utility, but it remains weighted toward retrospective technical validation.

### 3.3 Evolution of AI techniques in brain imaging: RQ1

#### 3.3.1 Traditional machine learning

Traditional machine learning remained relevant when cohorts were modest, inputs were structured, or neurobiological interpretation depended on explicit features. SVMs were frequently applied to regional volumes, radiomic descriptors, DTI metrics, and connectivity measures. In multicenter temporal-lobe epilepsy, SVM and deep-learning classifiers produced broadly similar performance on region-level structural and diffusion MRI, showing that model complexity did not compensate for limited representation granularity (Gleichgerrcht et al., 2021). Conventional classifiers also supported MRI differentiation of Alzheimer and vascular dementia when carefully selected features encoded disease-relevant anatomy (Castellazzi et al., 2020).

Random forests and ensemble models were used for nonlinear interaction modeling and mixed clinical-imaging inputs. Survival analysis for dementia prediction demonstrated that algorithm ranking depends on censoring, dimensionality, and feature-selection design rather than a universal superiority of one method (Spooner et al., 2020). Ensemble learning predicted MS disease course from multimodal baseline information and offered an interpretable alternative to end-to-end imaging networks (Zhao et al., 2020). These studies support continued use of classical baselines, especially when data are tabular, samples are limited, and calibration or feature attribution is clinically important.

Traditional pipelines were vulnerable to high-dimensional overfitting. Feature selection performed before cross-validation, slice-level partitioning, and repeated tuning against a test set can inflate results. Large-scale diagnostic classification studies have shown that data quantity, confound control, and validation design may matter more than classifier choice (Lanka et al., 2020). Accordingly, the strongest recent studies used nested validation, external cohorts, or site-wise testing rather than reporting a single random split.

#### 3.3.2 Convolutional and three-dimensional deep learning

CNNs were frequently represented in the included literature. Two-dimensional networks were used for CT slices, EEG time-frequency maps, and memory-constrained datasets, while three-dimensional networks were used when volumetric context was relevant. One common-framework study reported higher performance for deep residual representations than standard feature pipelines (Abrol et al., 2021), and lightweight networks were evaluated for brain-age estimation (Peng et al., 2021). These results arose from different populations and tasks and do not establish general superiority of CNNs.

U-Net variants dominated segmentation of infarcts, hemorrhage, glioma, MS lesions, and TBI abnormalities. CSNet used a dedicated deep framework for ischemic lesion segmentation (Kumar et al., 2020). Multimodal MRI segmentation of acute and subacute stroke benefited from combining sequences with complementary lesion contrast (Clèrigues et al., 2020). Hemorrhage systems on non-contrast CT quantified lesion volume in addition to detecting abnormal scans (Arab et al., 2020). Dense and inception-style designs improved multiscale feature integration for medical segmentation (Zhang Z. et al., 2020).

Three-dimensional CNNs were particularly valuable for glioma because tumor extent and molecular phenotype depend on spatial context across sequences. Context-aware systems combined segmentation, subtype classification, and survival-related assessment (Pei et al., 2020). Automated glioma grading on conventional MRI showed that deep volumetric patterns can support noninvasive characterization (Zhuge et al., 2020). Yet multi-institutional brain-extraction experiments in diffuse glioma demonstrated that apparently peripheral preprocessing can become a major source of model failure (Thakur et al., 2020).

CNN performance was strongest when the target had a visible imaging correlate and labels were reliable. Subtle network disorders, mixed dementia, small cortical lesions, and non-lesional epilepsy were more difficult. The comparison of CNN variants for Alzheimer classification found no consistent advantage over a well-designed SVM and identified leakage risks in much of the earlier literature (Wen et al., 2020). This result cautions against equating architectural sophistication with diagnostic validity.

#### 3.3.3 Representation and generative learning

Autoencoders supported dimensionality reduction, normative modeling, anomaly detection, and multimodal feature learning. Unsupervised anomaly segmentation in brain MRI remained sensitive to whether reconstruction errors represented pathology or normal anatomical variability (Baur et al., 2021). Sparse autoencoding of resting-state fMRI enabled ASD classification but inherited site and preprocessing sensitivity from functional connectivity estimation (Almuqhim and Saeed, 2021). Normative modeling offered an alternative by measuring individual deviation from reference distributions rather than forcing every subject into a disease class (Pinaya et al., 2021).

GANs were used to synthesize missing sequences, reduce acquisition burden, augment rare lesions, and harmonize data. In brain tumor segmentation, synthetic T1 and FLAIR sequences were evaluated by their effect on a downstream multisequence model rather than image appearance alone (Conte et al., 2021). Synthetic post-contrast T1-weighted MRI was assessed in a multicenter neuro-oncology cohort, demonstrating the importance of clinical task validation for generated images (Jayachandran Preetha et al., 2021). Data augmentation reviews nevertheless show that transformations can introduce unrealistic anatomy or alter disease prevalence when not constrained by domain knowledge (Goceri, 2023).

Self-supervised learning expanded pretraining beyond labeled neurological cohorts. Models Genesis demonstrated reusable three-dimensional representations learned through image transformations and restoration tasks (Zhou et al., 2021). Physics-guided self-supervised MRI reconstruction reduced reliance on fully sampled reference acquisitions (Yaman et al., 2020). FastMRI challenge results further illustrated the value of standardized reconstruction benchmarks, although perceptual quality and downstream diagnostic preservation remain separate endpoints (Muckley et al., 2021).

#### 3.3.4 Attention and transformer-based architectures

Attention modules enhanced spatial focus, channel reweighting, and multimodal interaction. Dual-attention multi-instance learning was applied to structural MRI for Alzheimer diagnosis, allowing the model to aggregate informative regions rather than treating all patches equally (Zhu W. et al., 2021). Attention-guided tumor segmentation improved representation of heterogeneous multimodal MRI (Ranjbarzadeh et al., 2021). These mechanisms can support interpretability, but attention weights should not be assumed to provide faithful causal explanations without perturbation or stability testing.

Transformers increased global receptive fields and modeled relationships among distant tokens. SwinBTS combined hierarchical Transformer processing with convolutional operations for three-dimensional multimodal brain tumor segmentation (Jiang et al., 2022). Hybrid CNN-Transformer multi-task learning integrated local texture with global context for simultaneous glioma segmentation and IDH genotyping (Cheng et al., 2022). Deep Transformer models also captured long-range temporal dependencies for seizure prediction (Bhattacharya et al., 2022).

Transformer-based methods appeared in a subset of recent studies, but their benefits were not uniform. Large models require substantial data, memory, and regularization. Patch tokenization can remove small-lesion detail, while natural-image pretraining may not transfer to quantitative volumetric signals. Selected hybrid systems retained convolutional inductive bias, multiscale features, or task-specific supervision; the heterogeneous evidence does not establish a universal advantage over CNN or U-Net baselines.

#### 3.3.5 Emerging AI paradigms

GNNs represented brain regions as nodes and anatomical or functional relationships as edges. A multi-view GCN guided by prior brain structure improved ASD classification and identified candidate subnetworks (Wen et al., 2022). Reviews of GNNs in network neuroscience emphasize that graph construction, parcellation, thresholding, and edge definition can materially change the learned representation (Bessadok et al., 2023). Graph models therefore offer biological alignment, but their validity depends on the upstream network definition.

Multimodal learning was the most consistent emerging direction. MRI, PET, clinical, genetic, and cognitive data improved Alzheimer staging compared with single-modality models (Venugopalan et al., 2021). PET/MRI radiomics supported differentiation of Parkinson disease and multiple system atrophy (Sun et al., 2024). Multimodal brain-tumor systems combined imaging and non-imaging information for prognosis across adult and pediatric cohorts (Steyaert et al., 2023). Flexible missing-data mechanisms were uncommon, however, and complete-case analysis may select unrepresentative patients.

Explainable AI, uncertainty estimation, federated learning, and foundation models appeared increasingly in discussion and early evaluation. A unified neuroimaging interpretation framework distinguished model explanation from neurobiological inference and warned against unstable *post hoc* maps (Kohoutová et al., 2020). Bayesian uncertainty improved risk stratification for subtle focal cortical dysplasia detection (Gill et al., 2021). Unlearning of site bias and confound information offered a representation-level harmonization strategy (Dinsdale et al., 2021). Table 2 summarizes the 15 principal method families, representative architectures, applications, strengths, and limitations.

**Table 2:** Summary of AI techniques applied to brain imaging and computer-aided neurological diagnosis.

AI technique	Representative architecture	Typical modalities	Primary applications	Principal strengths	Principal limitations	Representative evidence
Handcrafted ML	Logistic models, radiomics	MRI, CT, PET, tabular	Classification, prognosis	Interpretable features; suitable for modest samples	Feature instability; manual design; leakage during selection	Spooner et al. (2020), Carré et al. (2020), Castellazzi et al. (2020), Solana-Lavalle and Rosas-Romero (2021), Lanka et al. (2020)
Support vector machines	Linear/RBF SVM	MRI, DTI, fMRI	Disease classification, lateralization	Strong small-sample baseline; margin control	Sensitive to scaling and feature selection	Gleichgerrcht et al. (2021), Kim et al. (2020), Wen et al. (2020), Yasaka et al. (2021), Lanka et al. (2020)
Tree/ensemble ML	RF, XGBoost, stacked ensembles	MRI plus clinical data	Staging, progression, differential diagnosis	Nonlinear interactions; mixed inputs	Calibration and external transfer can be weak	Spooner et al. (2020), Theocharidis et al. (2026), Roca et al. (2020), Zhao et al. (2020)
k-NN/probabilistic ML	k-NN, naïve Bayes	Engineered MRI features	Baseline classification	Simple and transparent benchmarking	Curse of dimensionality; site sensitivity	Pan et al. (2020), Wen et al. (2020), Venugopalan et al. (2021)
2D CNN	ResNet, DenseNet, slice CNN	CT, MRI slices, EEG maps	Detection and classification	Efficient; leverages pretrained backbones	Ignores through-plane context; slice leakage risk	Pan et al. (2020), Wen et al. (2020), Ranjbarzadeh et al. (2021), Qiu W. et al. (2020)
3D CNN	3D ResNet, 3D DenseNet	Volumetric MRI/CT	Classification, morphometry, grading	Preserves volumetric context	Memory intensive; needs larger cohorts	Peng et al. (2021), Henschel et al. (2020), Naser and Deen, 2020, Duc et al. (2020), Thomas et al. (2020), Bashyam et al. (2020)
U-Net family	3D U-Net, dense/inception U-Net	MRI, CT	Lesion/structure segmentation	Multi-scale dense prediction; skip connections	Dice favors large lesions; domain shift	Arab et al. (2020), Clèrigues et al. (2020), Kumar et al. (2020), Gros et al. (2021), Liu et al. (2021), La Rosa et al. (2020), Li et al. (2021), Kamraoui et al. (2022), Wiltgen et al. (2024), Yu et al. (2020), Zhang Z. et al. (2020)
Autoencoders	Sparse, denoising, variational AE	MRI, fMRI	Anomaly detection, representation learning	Uses unlabeled data; compact latent space	Reconstruction error may not localize disease	Baur et al. (2021), Almuqhim and Saeed (2021), Pinaya et al. (2021)
Generative models	GAN, conditional GAN	Multisequence MRI	Synthesis, imputation, augmentation	Addresses missing contrasts and data scarcity	Hallucination; difficult fidelity assessment	Jayachandran Preetha et al. (2021), Conte et al. (2021), Finck et al. (2020)
RNN/temporal models	LSTM, GRU, temporal CNN	EEG, longitudinal data	Event detection, forecasting	Models sequential dependence	Nonstationarity; patient-specific leakage	Pavel et al. (2020), da Silva Lourenço et al. (2021), Fürbass et al. (2020), Gabeff et al. (2021)
Attention networks	Channel/spatial/dual attention	MRI, PET, CT	Classification and segmentation	Focuses informative regions/features	Attention is not automatically faithful explanation	Magesh et al. (2020), Ranjbarzadeh et al. (2021), El-Sappagh et al. (2021), Zhu W. et al. (2021)
Transformers	ViT, Swin, CNN-Transformer	3D MRI, EEG	Segmentation, classification, prediction	Global context and long-range interaction	Data and compute demand; small-lesion token loss	Bhattacharya et al. (2022), Cheng et al. (2022), Jiang et al. (2022)
Graph neural networks	GCN, graph attention	fMRI/DTI connectomes	Network diagnosis and subtyping	Aligns with relational brain organization	Graph construction and parcellation sensitivity	Bessadok et al. (2023), Park et al. (2021), Wen et al. (2022), Larivière et al. (2020)
Self-supervised/transfer	Contrastive, restoration, domain pretraining	MRI, radiological images	Pretraining, reconstruction, low-label learning	Reduces annotation dependence	Pretext-task and domain-transfer mismatch	Yaman et al. (2020), Muckley et al. (2021), Mei et al. (2022), Zhou et al. (2021)
Multimodal/emerging	Cross-modal fusion, uncertainty, federated/foundation strategies	MRI + PET/fMRI/DTI/clinical	Differential diagnosis, staging, prognosis	Integrates complementary evidence	Missingness, privacy, cost, and calibration	Boutet et al. (2021), Talai et al. (2021), Xue et al. (2024), Tang et al. (2026), Brugnara et al. (2020), Collins et al. (2024), Spitzer et al. (2022), Mongan et al. (2020), Venugopalan et al. (2021), Sun et al. (2024), Moons et al. (2025), Storelli et al. (2026), Amiri et al. (2023), Thompson et al. (2020), Qiu et al. (2022), Steyaert et al. (2023)

### 3.4 Brain imaging modalities in AI-based diagnosis: RQ2

#### 3.4.1 Magnetic resonance imaging

MRI was the most frequently represented modality in the descriptive coding because it supports volumetric anatomy, multiple tissue contrasts, diffusion, perfusion, and connectivity without ionizing radiation. This frequency does not prove diagnostic superiority over other modalities. Structural T1-weighted MRI was used for morphometry, brain age, dementia classification, and parkinsonian differentiation. Large-cohort studies and robust segmentation pipelines showed selected applications across heterogeneous MRI data, while scanner and protocol sensitivity remained important limitations (Bashyam et al., 2020; Henschel et al., 2020; Billot et al., 2023).

FLAIR was central to stroke, MS, edema, and tumor analysis because it suppresses cerebrospinal fluid and highlights many lesions. DWI was indispensable for acute ischemia, while perfusion MRI supported tissue-at-risk assessment and outcome-related modeling. DTI captured tract and microstructural change in epilepsy, parkinsonism, and demyelination. Deep diffusion enhancement required explicit uncertainty modeling because visually plausible reconstruction can conceal local error (Tanno et al., 2021).

MRI limitations were consistently reported. Acquisition time, motion, sequence availability, field strength, coil differences, and protocol variability create domain shift. Standardization across machines improves radiomic stability but cannot eliminate biological and acquisition differences (Carré et al., 2020). ComBat harmonization can increase statistical power when applied without leaking outcome information across partitions (Radua et al., 2020). Data-driven confound removal provides another option, although removing site information may also remove clinically relevant population structure (Dinsdale et al., 2021).

#### 3.4.2 Functional MRI and diffusion imaging

Resting-state fMRI was commonly converted into connectivity matrices or dynamic network features. Three-dimensional CNNs used temporal statistics of resting-state fMRI to classify ASD (Thomas et al., 2020). Site-harmonized ABIDE connectivity improved diagnostic classification relative to unharmonized multi-site data (Ingalhalikar et al., 2021). fMRI also supported Alzheimer assessment, Parkinson characterization, and stimulation-related targeting, but results were sensitive to motion, parcellation, temporal filtering, and the definition of connectivity.

Diffusion imaging supported white-matter lesion classification, tract analysis, and network construction. Diffusion basis spectrum imaging enabled classification of MS lesion types (Ye et al., 2020). Structural connectome matrices were used for Parkinson diagnosis and neurocircuit investigation (Yasaka et al., 2021). In epilepsy, diffusion features supported TLE identification and lateralization, but ROI-level SVM and deep models produced only moderate gains (Gleichgerrcht et al., 2021). These findings suggest that biologically informed features may be more important than network depth when samples are limited.

#### 3.4.3 Computed tomography

CT was concentrated in acute stroke, hemorrhage, aneurysm, and TBI. Non-contrast CT models detected early infarction and approximated diffusion-MRI lesion volume (Qiu W. et al., 2020). Multiphase CT angiography CNNs detected large-vessel occlusion (Stib et al., 2020). CT angiography systems also identified intracranial aneurysms with clinically oriented validation (Shi et al., 2020). Deep networks quantified hemorrhagic lesions on brain CT and enabled structured triage outputs (Li et al., 2021).

Real-world evidence was stronger for CT than for many other modalities because stroke workflows permit prospective observation of time-sensitive endpoints. Automated intracranial hemorrhage detection was evaluated within emergency imaging operations (Seyam et al., 2022). AI-enabled large-vessel occlusion alerts were associated with shorter treatment workflow intervals in a stepped-wedge cluster trial (Martinez-Gutierrez et al., 2023). A multinational 2026 reader study extended large-vessel occlusion evaluation to non-contrast CT, addressing access where angiography is not immediately available (Sunwoo et al., 2026).

CT limitations include radiation, lower soft-tissue contrast, variable reconstruction kernels, and dependence on disease prevalence. Performance can fall for distal occlusions, subtle lacunar infarcts, or uncommon hemorrhage patterns. Automated deep learning and clinician comparison for lacunar stroke on CT perfusion illustrates the need to evaluate difficult subtypes rather than only conspicuous proximal disease (Thomas et al., 2026).

#### 3.4.4 Positron emission tomography and SPECT

PET and SPECT contributed molecular, metabolic, and neurotransmitter information. MRI-PET fusion improved Alzheimer diagnosis by combining anatomical and metabolic evidence (Song et al., 2021). Interpretable adversarial decomposition of PET signals aimed to separate Alzheimer pathological processes with stronger clinical relevance (Tang et al., 2026). DaTSCAN explainability studies examined whether image regions driving Parkinson classification corresponded to expected striatal patterns (Magesh et al., 2020).

Deep metabolic indices supported differential diagnosis of parkinsonism (Wu et al., 2022). Dopamine-transporter imaging models also differentiated parkinsonian syndromes using deep learning (Zhao et al., 2022). PET/SPECT limitations included smaller cohorts, tracer and scanner variability, access cost, and potential circularity when clinical diagnosis already incorporated the same imaging result. Independent evaluation and comparison against blinded specialist interpretation were therefore particularly important.

#### 3.4.5 EEG, MEG, and multimodal neuroimaging

EEG extended AI-assisted brain assessment beyond tomographic images. Multicenter AI interpreted routine EEG into clinically meaningful abnormality categories and achieved high discrimination across independent test sets (Tveit et al., 2023). Human-supervised hybrid systems detected interictal epileptiform discharges while retaining expert oversight (Kural et al., 2022). Earlier algorithms were validated against clinical reference standards for epileptiform discharge detection (Fürbass et al., 2020). Interpretability work further examined whether seizure-detection networks relied on physiologically plausible temporal features (Gabeff et al., 2021).

MEG evidence was smaller and focused on source localization, connectivity, and dementia or epilepsy biomarkers. Its high temporal resolution is valuable, but specialized hardware and complex inverse modeling limit dataset scale. Multimodal combinations were more common than MEG-only CAD. Structural-functional integration, PET/MRI fusion, CT plus clinical data, and imaging plus demographic or cognitive variables consistently offered richer context, although missingness and synchronization remained challenges.

Figure 3 illustrates 923 explicit modality coding events in the auxiliary mapping set. MRI accounted for the largest share, followed by multimodal imaging and fMRI. Figure 4 depicts non-mutually-exclusive disease-modality co-occurrences and shows the expected concentration of CT in stroke, PET in dementia and parkinsonism, EEG in epilepsy, and MRI across nearly all disease groups. Table 3 compares the information content, applications, advantages, and limitations of 10 modality categories.

**Figure 3:**
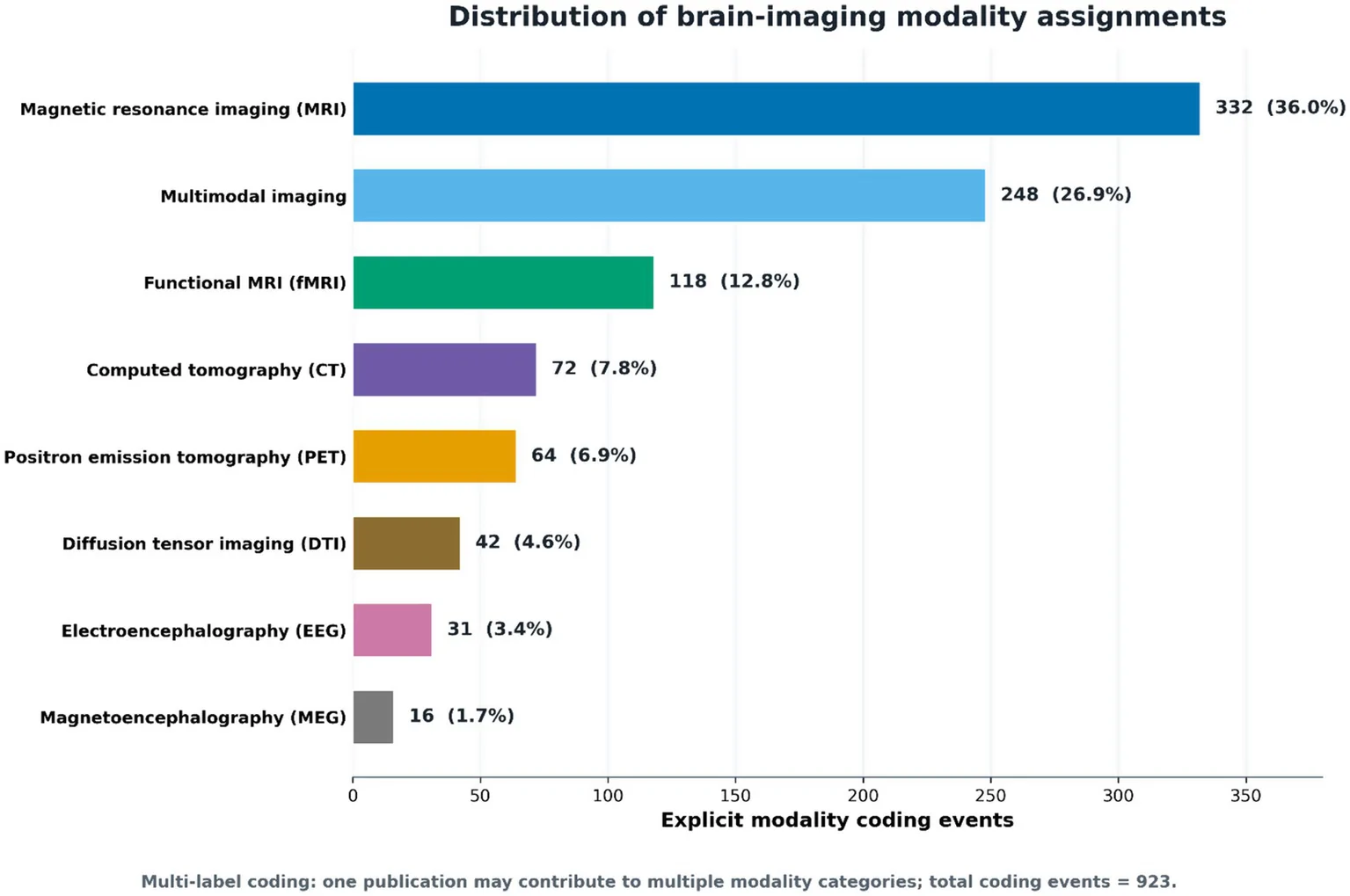
Distribution of brain-imaging modality assignments in the auxiliary mapping data. One coding event is one publication-to-modality assignment. Because a publication could be assigned to multimodal imaging and to each component modality, the 923 events are non-mutually exclusive and do not represent 923 independent studies.

**Figure 4:**
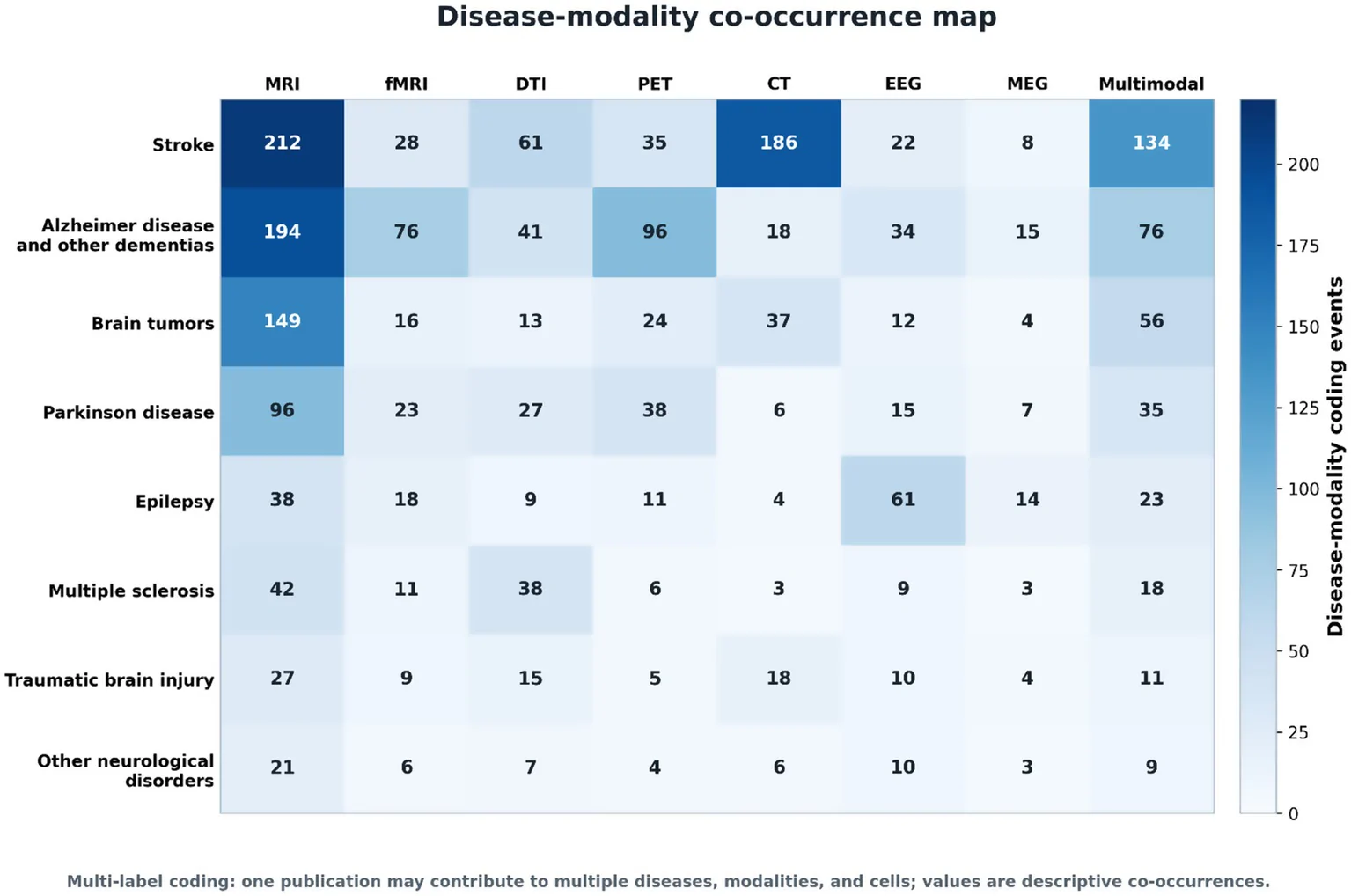
Disease-modality co-occurrence map in the auxiliary multi-label coding data. One coding event is one publication-to-disease-to-modality assignment. A publication could contribute to multiple diseases, modalities, and cells; values are descriptive, non-mutually-exclusive co-occurrences and must not be summed as independent study counts.

**Table 3:** Comparative analysis of neuroimaging modalities for AI-based neurological disease diagnosis.

Modality	Information captured	Major diseases	Common AI methods	Primary tasks	Advantages / limitations	Representative evidence
Structural MRI	Anatomy, atrophy, morphology	Dementia, tumors, PD, epilepsy	3D CNN, radiomics, Transformer	Classification, segmentation, staging	Rich contrast and no radiation; protocol and scanner shift	Abrol et al. (2020), Yin et al. (2023), Wen et al. (2020), Camacho et al. (2023), Qiu S. et al. (2020), Bashyam et al. (2020)
FLAIR / T2 MRI	Edema, WM lesions, gliosis	Stroke, MS, tumors	U-Net, CNN, GAN	Lesion detection and burden	Clinically common, intensity and lesion-definition variability	La Rosa et al. (2020), Conte et al. (2021), Storelli et al. (2022), Kamraoui et al. (2022), Wiltgen et al. (2024)
DWI / perfusion MRI	Restricted diffusion and hemodynamics	Acute stroke, tumors	3D CNN, U-Net, ML	Infarct detection, tissue prediction	High acute sensitivity; artifacts and timing effects	Liu et al. (2021), Nishi et al. (2020), Thomas et al. (2026), Yu et al. (2020), Ye et al. (2020)
Functional MRI	Activation and functional connectivity	Dementia, PD, epilepsy, ASD	CNN, GNN, SVM	Network diagnosis, lateralization	Functional information; motion and pipeline sensitivity	Boutet et al. (2021), Almuqhim and Saeed (2021), Wen et al. (2022), Khodatars et al. (2021), Thomas et al. (2020)
DTI / diffusion	WM microstructure and tracts	PD, epilepsy, MS	SVM, CNN, connectome/GNN	Classification, tract assessment	Microstructural specificity; crossing-fiber and harmonization limits	Talai et al. (2021), Gleichgerrcht et al. (2021), Yasaka et al. (2021), Chougar et al. (2021), Larivière et al. (2020)
CT / CTA / CTP	Hemorrhage, density, vessels, perfusion	Stroke, TBI, aneurysm	2D/3D CNN, U-Net	Triage, detection, segmentation	Fast and available; radiation and lower soft-tissue contrast	Arab et al. (2020), Flanders et al. (2020), Martinez-Gutierrez et al. (2023), Li et al. (2021), Matsoukas et al. (2023), Qiu W. et al. (2020)
PET / SPECT	Metabolism, amyloid/tau, dopamine transport	Dementia, parkinsonism, tumors	Radiomics, CNN, multimodal fusion	Differential diagnosis, staging	Molecular specificity; cost, tracer, and sample-size limits	Tang et al. (2026), Song et al. (2021), Sun et al. (2024), Hosseinzadeh et al. (2023), Magesh et al. (2020)
EEG	Electrical dynamics and epileptiform activity	Epilepsy and diffuse dysfunction	Temporal CNN, RNN, Transformer	Detection, classification, forecasting	High temporal resolution; artifact and nonstationarity	Pavel et al. (2020), da Silva Lourenço et al. (2021), Fürbass et al. (2020), Tveit et al. (2023), Gabeff et al. (2021)
MEG	Magnetic neural dynamics and source estimates	Epilepsy, dementia, network disorders	Source ML, connectivity, GNN	Localization and network assessment	Excellent temporal resolution; specialized access and inverse problem	Bessadok et al. (2023), Kohoutová et al. (2020), Khodatars et al. (2021), Thompson et al. (2020)
Multimodal	Complementary structural, functional, molecular, and clinical evidence	All major groups	Fusion, multitask, cross-modal attention	Differential diagnosis, staging, prognosis	Potentially comprehensive; missingness, cost, synchronization	Xue et al. (2024), Brugnara et al. (2020), Venugopalan et al. (2021), Amiri et al. (2023), Qiu et al. (2022), Zhang Y. D. et al. (2020)

### 3.5 AI applications across neurological diseases: RQ3

#### 3.5.1 Stroke

Stroke research addressed early ischemia, final infarct prediction, vessel occlusion, intracranial hemorrhage, lesion segmentation, onset time, and outcome. Deep DWI models trained on 2,348 clinical scans and tested externally improved detection of small diffusion abnormalities (Liu et al., 2021). U-Net prediction of final infarct from baseline MRI achieved a median AUC of 0.92 in a multicenter cohort, although Dice overlap remained moderate (Yu et al., 2020). Automated DWI-FLAIR modeling also estimated stroke onset time for patients with an unknown window (Zhu H. et al., 2021).

Machine learning combined imaging and clinical variables for endovascular outcome assessment (Brugnara et al., 2020). High-level neuroimaging features predicted outcome after large-vessel occlusion (Nishi et al., 2020). Post-stroke cognitive impairment models broadened CAD beyond acute lesion recognition (Lee et al., 2023). The most clinically mature systems were CT-based triage tools, but distal vessel detection, domain shift, false alerts, and uncertain effect on patient outcomes remained unresolved.

#### 3.5.2 Alzheimer’s disease and other dementias

Dementia studies progressed from binary Alzheimer-versus-control classification to MCI progression, mixed pathology, disease staging, and etiological differential diagnosis. Interpretable multimodal learning achieved strong external AUC values across ADNI, AIBL, Framingham, and NACC cohorts (Qiu S. et al., 2020). Independent validation of an MRI-derived dementia score used 8,834 images from ADNI, AIBL, OASIS, and MIRIAD (Popuri et al., 2020). Normative models characterized individual progression rather than relying solely on group classification (Pinaya et al., 2021).

Multimodal frameworks accepted flexible clinical, cognitive, and imaging inputs for Alzheimer and non-Alzheimer dementia assessment (Qiu et al., 2022). A 2024 system analyzed 51,269 participants across nine datasets and differentiated ten dementia etiologies (Xue et al., 2024). Deep residual learning was also used to predict progression to Alzheimer disease (Abrol et al., 2020). In 2026, ensemble MRI models and interpretable PET decomposition reflected continued movement toward robust, complementary biomarkers (Theocharidis et al., 2026).

Despite high AUC values, external performance varied with inclusion criteria, demographics, and diagnostic labels. Structural MRI may encode age, site, and cognitive severity that correlate with diagnosis but do not uniquely represent pathology. Multimodal explanation models attempted to connect predictions to clinical features and brain regions (El-Sappagh et al., 2021). The field increasingly requires biomarker-confirmed endpoints and evaluation of mixed disease rather than data-set-specific diagnostic labels.

#### 3.5.3 Brain tumors

Brain tumor studies concentrated on multimodal MRI segmentation, glioma grading, molecular markers, and integrated diagnosis. A hybrid CNN-radiomics system predicted IDH status across internal and two external datasets, with AUC decreasing from 0.96 internally to 0.86 in TCIA (Choi et al., 2021). Deep learning predicted MGMT promoter methylation from MRI, although later benchmark experience has shown that molecular labels can be difficult to infer reliably from imaging alone (Yogananda et al., 2021). Histopathology-validated radiomics differentiated true progression from pseudoprogression (Akbari et al., 2020).

Multi-task systems reduced fragmentation among segmentation, molecular subtyping, and grading. A 1,508-patient development cohort and 240-patient independent test set supported simultaneous IDH, 1p/19q, grade, and tumor delineation (van der Voort et al., 2023). Hybrid CNN-Transformer learning linked segmentation and IDH genotyping (Cheng et al., 2022). Multimodal prognosis models combined clinical and imaging representations across adult and pediatric tumors (Steyaert et al., 2023).

AI also extended beyond MRI. Stimulated Raman histology with CNNs provided near-real-time intraoperative tumor diagnosis and achieved noninferior accuracy to conventional pathologist interpretation in a prospective multicenter trial (Hollon et al., 2020). Later work used rapid label-free optical imaging for molecular classification of diffuse glioma (Hollon et al., 2023). These systems demonstrate that “brain imaging” CAD includes intraoperative tissue imaging when the diagnostic target is neurological disease.

#### 3.5.4 Parkinson’s disease

Parkinson studies used T1 MRI, diffusion, fMRI, neuromelanin-sensitive MRI, DAT SPECT, FDG PET, and multimodal PET/MRI. Voxel-based morphometry and conventional machine learning on PPMI MRI reported high sex-stratified accuracy, but the absence of a clearly separated external site limits generalization claims (Solana-Lavalle and Rosas-Romero, 2021). A multicenter MRI study distinguished Parkinson disease from progressive supranuclear palsy and multiple-system atrophy under clinical scanner variation (Chougar et al., 2021). A large 13-study T1 MRI dataset produced an explainable CNN with an AUC of 0.87 and similar independent performance (Camacho et al., 2023).

Radiomics combined whole-brain functional activity and gray-matter structure (Cao et al., 2020). White-matter radiomics supported longitudinal progression prediction (Shu et al., 2021). DAT SPECT and PET-based systems focused on differential diagnosis rather than simple case–control discrimination (Wu et al., 2022). MRI-based cortical thickness also predicted conversion to dementia in Parkinson disease (Shin et al., 2021). Multimodal PET/MRI improved separation of Parkinson disease and multiple-system atrophy, but test-set uncertainty remained substantial in the smaller cohort (Sun et al., 2024).

#### 3.5.5 Epilepsy

Epilepsy applications divided into electrophysiological event analysis, MRI lesion detection, network-based diagnosis, and surgical assessment. Multicenter focal cortical dysplasia models addressed lesions missed on routine MRI and incorporated uncertainty for risk stratification (Gill et al., 2021). The MELD study harmonized 1,015 participants from 22 centers and released an interpretable surface-based pipeline (Spitzer et al., 2022). MRI heterogeneity modeling identified distinct epilepsy atrophy trajectories (Xiao et al., 2023).

EEG models detected seizures or epileptiform discharges, forecast risk, and generated structured interpretations. A neonatal seizure algorithm was evaluated in a multicenter randomized trial, an uncommon level of evidence for neurological AI (Pavel et al., 2020). AI-supervised expert labeling improved identification of interictal discharges (Kural et al., 2022). Transformer prediction and routine-EEG interpretation expanded the temporal modeling scope (Bhattacharya et al., 2022). However, event prevalence, patient-specific leakage, recording hardware, and nonstationarity complicate comparison.

#### 3.5.6 Multiple sclerosis

MS studies focused on lesion segmentation, central vein sign detection, subtype discovery, differential diagnosis, disability, and progression. Three-dimensional U-Net segmentation of cortical and white-matter lesions used FLAIR and MP2RAGE across two centers (La Rosa et al., 2020). CVSnet automated central-vein-sign assessment (Maggi et al., 2020). Deep models differentiated neuromyelitis optica spectrum disorder from MS (Kim et al., 2020).

Unsupervised MRI learning identified data-driven MS subtypes with different clinical trajectories (Eshaghi et al., 2021). FLAIR-based AI predicted two-year disability, but external validation remained necessary (Roca et al., 2020). DeepLesionBrain emphasized cross-dataset robustness over benchmark-specific optimization (Kamraoui et al., 2022). The open-source LST-AI ensemble improved lesion segmentation across public tests and exceeded comparison systems in the reported evaluation (Wiltgen et al., 2024). Explainable multimodal modeling in 2026 extended MRI and clinical prediction to neurocognitive progression (Storelli et al., 2026).

#### 3.5.7 Other neurological disorders

TBI models segmented multiclass hemorrhage and edema on CT using multicenter development and external validation (Monteiro et al., 2020). Deep CT systems also predicted severe TBI outcome, illustrating the shift from lesion burden toward individualized assessment (Pease et al., 2022). Multimodal prediction of residual consciousness combined complementary intensive-care measures, although clinical deployment requires careful uncertainty communication (Amiri et al., 2023).

ASD research used fMRI connectivity, autoencoders, CNNs, and graph models. ComBat harmonization improved ABIDE classification across several algorithms (Ingalhalikar et al., 2021). A multi-view GCN learned functional subnetworks and achieved modest ABIDE accuracy, underscoring the heterogeneity of ASD rather than a simple imaging biomarker (Wen et al., 2022). Connectome and microcircuit modeling identified altered subcortico-cortical interactions (Park et al., 2021). Table 4 presents 30 representative disease-specific studies with modality, cohort, task, validation, and reported performance.

**Table 4:** Representative AI-based brain imaging studies across major neurological diseases.

Study	Disease	Modality / data	Cohort or benchmark	AI method and task	Validation / metric	Principal reported finding
Wen et al. (2020)	AD	T1 MRI	ADNI, AIBL, OASIS	2D/3D CNN classification	Leakage-safe hold-out; accuracy/AUC	3D variants were similar; 2D was lower; CNN did not consistently exceed SVM.
Qiu S. et al. (2020)	AD	MRI + clinical	417 development; 1,066 across 3 external cohorts	Interpretable FCN classification	External AUC	AUC 0.996 internally and 0.876–0.974 across independent cohorts.
Qiu et al. (2022)	Dementia	Multimodal clinical + imaging	Multi-cohort	Flexible deep differential diagnosis	Expert and pathology comparison	Supported NC, MCI, AD, and non-AD dementia assessment with disease-specific maps.
Xue et al. (2024)	Dementia	Multimodal	51,269 participants; 9 datasets	Etiological differential diagnosis	Micro-AUROC	AUROC 0.94 for cognitive state and 0.96 for dementia etiology.
Pan et al. (2020)	AD	MRI	Public MRI cohort	CNN + ensemble classification	Internal accuracy	Combined learned and ensemble evidence for early-stage classification.
Liu et al. (2022)	AD	Structural MRI	Multi-cohort	Generalizable deep classification	External evaluation	Emphasized transfer across structural-MRI cohorts rather than a single benchmark.
Qiu W. et al. (2020)	Stroke	Non-contrast CT	Acute ischemic stroke	ML infarct segmentation	Volume agreement	Automated infarct extent agreed with diffusion-MRI reference volume.
Liu et al. (2021)	Stroke	DWI MRI	2,348 development; 280 external	Deep lesion detection/segmentation	External lesion metrics	Improved small-lesion detection and retained performance under clinical heterogeneity.
Yu et al. (2020)	Stroke	Baseline DWI/perfusion MRI	182 patients	U-Net final-infarct prediction	AUC, Dice, volume error	Median AUC 0.92 and Dice 0.53 for final lesion prediction.
Hakim et al. (2021)	Stroke	CT perfusion	ISLES challenge	ML infarct-core prediction	Challenge comparison	Highlighted cross-method lessons and the effect of reference definition on infarct-core estimates.
Matsoukas et al. (2023)	Stroke	CTA	1,822 prospective CTAs	Commercial LVO detection	Sensitivity/specificity/AUC	AUC 0.95 for ICA-T/M1, with reduced sensitivity for M2 occlusion.
Hollon et al. (2020)	Brain tumor	Stimulated Raman histology	278-patient prospective multicenter trial	CNN intraoperative diagnosis	Accuracy; noninferiority	94.6% CNN accuracy versus 93.9% conventional pathologist interpretation.
Choi et al. (2021)	Glioma	Multimodal MRI	1,166 scans; 2 external sets	CNN-radiomics IDH prediction	Dice and external AUC	Segmentation Dice 0.86–0.92; IDH AUC declined from 0.96 internal to 0.86 external.
van der Voort et al. (2023)	Glioma	3D structural MRI	1,508 development; 240 external	Multitask subtype/grade/segmentation	AUC and Dice	External IDH AUC 0.90, 1p/19q AUC 0.85, grade AUC 0.81, whole-tumor Dice 0.84.
Calabrese et al. (2020)	Glioblastoma	Preoperative MRI	Institutional/TCIA imaging	Automated genetic-alteration prediction	Cross-validation	Demonstrated noninvasive prediction of selected molecular alterations from imaging.
Naser and Deen (2020)	Lower-grade glioma	MRI	Public clinical images	Deep segmentation and grading	Dice/classification metrics	Combined tumor delineation with grade-related classification.
Chougar et al. (2021)	Parkinsonism	MRI + DTI	322 subjects; clinical replication n = 143	Supervised differential classification	Balanced accuracy/AUC	Replication AUC 0.907–0.995 for several pairwise syndromic comparisons.
Camacho et al. (2023)	PD	T1 MRI	2,041 scans; 13 studies	Explainable CNN classification	Independent test AUC	Accuracy 79.3% and AUC 0.87 with similar independent performance.
Sun et al. (2024)	PD vs. MSA	PET/MRI + clinical	119 participants	Radiomics fusion	Train/test AUC	Combined-model test AUC 0.86; multimodal radiomics improved diagnostic context.
Boutet et al. (2021)	PD stimulation	fMRI + clinical	DBS cohort	ML parameter prediction	Out-of-sample prediction	Functional imaging supported individualized estimation of effective stimulation settings.
Gill et al. (2021)	Epilepsy/FCD	T1 + FLAIR MRI	148 multicenter; 23 independent	CNN detection + uncertainty	Sensitivity/specificity	Sensitivity 93% in leave-one-site-out analysis and 83% independently.
Spitzer et al. (2022)	Epilepsy/FCD	Surface MRI features	1,015 participants; 22 centers	Interpretable neural detection	Held-out/site tests	Gold-standard sensitivity 85%; full withheld cohort sensitivity 59%.
Lee et al. (2022)	Epilepsy	Structural MRI	Multicenter cohort	Unsupervised phenotype decomposition	Trajectory stability	Identified distinct atrophy progression trajectories across epilepsy subtypes.
da Silva Lourenço et al. (2021)	Epilepsy	Clinical EEG	Routine recordings	ML discharge detection	Expert-reference accuracy	Automated identification of interictal epileptiform discharges with expert-grounded evaluation.
La Rosa et al. (2020)	MS	FLAIR + MP2RAGE	90 patients; 2 centers	3D U-Net lesion segmentation	Detection/false positives	76% detection for cortical and WM lesions with cross-center evaluation.
Wiltgen et al. (2024)	MS	T1 + FLAIR	491 training; 103 public tests	3D U-Net ensemble	Dice/F1	MSSEG-1 Dice 0.65 and F1 0.63; released as open-source LST-AI.
Storelli et al. (2022)	MS	Longitudinal MRI	Clinical cohort	Deep progression prediction	Longitudinal discrimination	Modeled disease progression from MRI rather than lesion burden alone.
Rakić et al. (2021)	MS	Multisequence MRI	Public/institutional	Hybrid supervised-unsupervised segmentation	Cross-dataset lesion metrics	Improved lesion detection by combining unsupervised and supervised components.
Monteiro et al. (2020)	TBI	Head CT	839 multicenter scans; 500 external	Multiclass CNN segmentation	Volume agreement/detection	Quantified hemorrhage and edema with multicenter and external validation.
Ingalhalikar et al. (2021)	ASD	Resting-state fMRI	ABIDE multisite	ComBat + connectivity classifiers	Cross-site accuracy	Harmonization improved classification across several models and reduced site effects.

Large population cohorts extend this logic from categorical diagnosis to developmental and transdiagnostic risk modeling. A morphometric-genomic study of childhood and adolescent internalizing/externalizing comorbidity linked cortical surface patterns to genetic and cell-specific expression signals, illustrating how high-dimensional structural MRI can separate shared from disorder-specific neurodevelopmental effects (Kuang et al., 2023). Longitudinal ABCD data from 11,270 participants further showed that maturation of structural brain asymmetry was associated with cognitive performance and transdiagnostic psychiatric outcomes during adolescence (Wu et al., 2025). These findings do not establish a clinical diagnostic biomarker and were outside the 123-study neurological-disease evidence set, but they demonstrate a relevant modeling transition: AI can treat regional asymmetry, developmental timing, and multivariate morphometry as continuous risk features rather than forcing heterogeneous youth into binary labels. Future systems should evaluate age-specific normative models, longitudinal change, and transport across ancestry, scanner, and socioeconomic strata.

### 3.6 Computer-aided diagnostic tasks: RQ3

Classification and diagnosis were the most common tasks, ranging from binary case–control separation to multi-class disease differentiation. Detection systems located hemorrhage, infarct, large-vessel occlusion, cortical dysplasia, tumor, or abnormal EEG. Segmentation produced voxel-level lesion or structure masks and commonly served as an upstream step for radiomics, volume quantification, or molecular classification. Localization was especially important in epilepsy, stroke, and surgical neuro-oncology.

Differential diagnosis demanded more clinically realistic labels. Examples included Alzheimer versus vascular dementia, Parkinson disease versus atypical parkinsonism, MS versus neuromyelitis optica, and multiple dementia etiologies. Disease grading and staging appeared in glioma, dementia, and epilepsy progression. Outcome-related assessment included infarct evolution, cognitive decline, MS disability, tumor prognosis, and TBI outcome, but these tasks often depended on non-imaging variables and were more vulnerable to treatment and follow-up confounding.

Architecture choice followed output granularity. U-Net variants dominated dense segmentation. CNN and Transformer encoders supported examination-level classification. GNNs modeled connectivity. Recurrent or temporal-attention models analyzed EEG and longitudinal observations. Multi-task systems shared representations across segmentation and molecular or clinical outputs. This relationship argues against ranking models without specifying the diagnostic task and target granularity.

Task granularity exposed an important distinction between pure Vision Transformers and dense prediction. Examination-level classification can benefit from global token interactions even when spatial detail is coarsened. Fine-grained segmentation requires local continuity, multiscale boundaries, and sensitivity to lesions smaller than a patch; fixed patchification or aggressive token merging can remove evidence that decoder upsampling cannot recover. In this corpus, the strongest Transformer-based segmentation systems were hierarchical or hybrid: SwinBTS and CNN-Transformer multi-task models retained multiscale decoding, skip connections, or convolutional locality, whereas convincing evidence for a universal advantage of pure Vision Transformers on small-lesion segmentation was sparse (Cheng et al., 2022; Jiang et al., 2022). This does not prove an inherent Transformer deficit because data scale, pretraining, patch size, and decoder design are confounded. It does support task-specific reporting of lesion-size-stratified Dice and recall, boundary error or HD95, native-resolution ablations, and matched U-Net or hybrid baselines in addition to global classification metrics.

### 3.7 Datasets and data processing: RQ4

Public datasets enabled reproducibility and cross-study comparison. ADNI, AIBL, OASIS, and related cohorts supported dementia classification and progression. BraTS and TCGA/TCIA resources supported glioma segmentation and molecular studies. ISLES and stroke-lesion repositories supported infarct segmentation. ABIDE supported ASD connectivity analysis. PPMI supported Parkinson imaging. HCP, UK Biobank, ENIGMA, IXI, and OpenNeuro provided normative, population, or cross-disorder data.

Dataset reuse had two opposing effects. Standard benchmarks accelerated method development and made code comparison possible. Repeated tuning to familiar datasets also risks hidden test-set adaptation, narrow demographic representation, and inflated confidence. The RSNA intracranial hemorrhage challenge demonstrated the organizational value of collaborative dataset creation (Flanders et al., 2020). RadImageNet provided a domain-specific pretraining resource, but its heterogeneous modalities and labels do not eliminate the need for neurological fine-tuning (Mei et al., 2022). Table 5 describes 15 major public datasets and benchmarks, their modality, scale, labels, and typical tasks.

**Table 5:** Major public datasets and benchmarks used in AI-based brain imaging research.

Dataset / benchmark	Disease / population	Modality	Approximate scale	Labels / annotations	Typical AI tasks	Representative use in 2020–2026
BraTS / TCGA-TCIA	Glioma	Multimodal MRI	Hundreds to >1,500 cases; year-dependent	Tumor subregions, grade, molecular labels	Segmentation, subtype, prognosis	Yogananda et al. (2021), Calabrese et al. (2020), Conte et al. (2021), Cheng et al. (2022), Pei et al. (2020), Naser and Deen (2020), Ranjbarzadeh et al. (2021), Thakur et al. (2020), Steyaert et al. (2023), van der Voort et al. (2023), Zhuge et al. (2020), Jiang et al. (2022), Choi et al. (2021)
ADNI	Aging, MCI, AD	MRI, PET, clinical, biomarkers	Thousands; longitudinal phases	Diagnosis, cognition, biomarker status	Classification, staging, progression	Abrol et al. (2020), Xue et al. (2024), Pan et al. (2020), Theocharidis et al. (2026), Beheshti et al. (2020), Wen et al. (2020), Song et al. (2021), Venugopalan et al. (2021), Popuri et al. (2020), Qiu S. et al. (2020), El-Sappagh et al. (2021), Liu et al. (2022), Qiu et al. (2022), Zhu W. et al. (2021), Pinaya et al. (2021), Yang et al. (2021)
AIBL	Aging and AD	MRI, PET, clinical	Longitudinal; version-dependent	Cognition and biomarker labels	External validation, progression	Xue et al. (2024), Mårtensson et al. (2020), Wen et al. (2020), Popuri et al. (2020), Qiu S. et al. (2020), Bashyam et al. (2020)
OASIS	Normal aging and dementia	Structural MRI; selected PET/clinical	Thousands of sessions; release-dependent	Diagnosis, cognition, longitudinal visits	Classification, morphometry, external testing	Mårtensson et al. (2020), Wen et al. (2020), Popuri et al. (2020), Henschel et al. (2020), Qiu S. et al. (2020), Bashyam et al. (2020)
ISLES	Ischemic stroke	DWI, perfusion MRI/CT	Challenge-specific, multicenter	Lesion/core masks	Segmentation, tissue prediction	Clèrigues et al. (2020), Kumar et al. (2020), Hakim et al. (2021), Liu et al. (2021), Nishi et al. (2020), Zhu H. et al. (2021), Roca et al. (2020), Qiu W. et al. (2020), Yu et al. (2020)
ATLAS	Chronic stroke	T1 MRI	Approximately 1,000 scans in recent releases	Manual lesion masks	Lesion segmentation, outcome modeling	Clèrigues et al. (2020), Billot et al. (2023), Liu et al. (2021), Lee et al. (2023), Yu et al. (2020)
ABIDE	ASD and controls	Resting-state fMRI, structural MRI	>2,000 across ABIDE I/II	Diagnosis, site, phenotypic variables	Connectivity classification, harmonization	Almuqhim and Saeed (2021), Wen et al. (2022), Khodatars et al. (2021), Ingalhalikar et al. (2021), Thomas et al. (2020)
PPMI	PD and controls	MRI, DAT SPECT, clinical	Large longitudinal multicenter cohort	Diagnosis, motor/cognitive measures	Classification, subtyping, progression	Solana-Lavalle and Rosas-Romero (2021), Camacho et al. (2023), Hosseinzadeh et al. (2023), Shin et al. (2021), Magesh et al. (2020), Cao et al. (2020), Zhao et al. (2022), Shu et al. (2021)
HCP	Normative populations	Structural, fMRI, diffusion	About 1,200 young adults plus extensions	Connectome and behavioral measures	Pretraining, normative network analysis	Bessadok et al. (2023), Park et al. (2021), Yin et al. (2023), Lanka et al. (2020), Thompson et al. (2020)
UK Biobank imaging	Population cohort	MRI, diffusion, fMRI	Tens of thousands imaged; expanding	Health, genetics, longitudinal outcomes	Brain age, risk modeling, pretraining	Yin et al. (2023), Peng et al. (2021), Beheshti et al. (2020), Thompson et al. (2020), Bashyam et al. (2020)
ENIGMA	Cross-disorder consortia	MRI, DTI, derived phenotypes	International mega/meta-analysis scale	Harmonized regional/network measures	External validation, disease signatures	Eshaghi et al. (2021), Gleichgerrcht et al. (2021), Radua et al. (2020), Thompson et al. (2020), Sisodiya et al. (2020)
RSNA ICH Challenge	Intracranial hemorrhage	Head CT	Large labeled CT challenge	Hemorrhage type/examination labels	Detection, triage, segmentation	Arab et al. (2020), Flanders et al. (2020), Li et al. (2021), Monteiro et al. (2020), Seyam et al. (2022)
fastMRI	MRI reconstruction	Raw k-space MRI	Thousands of examinations	Acquired and reconstructed images	Accelerated reconstruction, pretraining	Yaman et al. (2020), Muckley et al. (2021), Mei et al. (2022), Zhou et al. (2021)
MELD	Focal cortical dysplasia	T1/FLAIR surface features	1,015 participants; 22 centers	Lesion masks, surgical/clinical labels	Interpretable lesion detection	Spitzer et al. (2022), Gill et al. (2021), Sisodiya et al. (2020)
MSSEG / ISBI MS	Multiple sclerosis	T1, T2, FLAIR, related MRI	Challenge-specific multicenter cohorts	Expert WM lesion masks	Lesion segmentation and generalization	Gros et al. (2021), La Rosa et al. (2020), Rakić et al. (2021), Maggi et al. (2020), Bouman et al. (2023), Kamraoui et al. (2022), Finck et al. (2020), Wiltgen et al. (2024)

Preprocessing most often included registration to a standard space, skull stripping, bias correction, resampling, intensity normalization, and augmentation. fMRI and diffusion studies additionally required motion correction, temporal filtering, tract or connectivity construction, and parcellation. ANTsX and similar ecosystems improved workflow reproducibility (Tustison et al., 2021). Synthesized or reconstructed images required evaluation for anatomical preservation and downstream diagnostic validity.

Scanner harmonization was a recurring requirement. ComBat reduced systematic site differences in morphometric and connectivity features (Radua et al., 2020). Deep unlearning removed dataset bias from MRI representations (Dinsdale et al., 2021). Multi-site harmonization improved ABIDE connectivity classification (Ingalhalikar et al., 2021). Nevertheless, harmonization performed before data partitioning can leak test-distribution information, and excessive adjustment can suppress genuine population differences.

Upstream spatial normalization can itself create a domain. The reviewed MRI pipelines variously used MNI152-like adult atlases, package-specific templates, study-built templates, or native-space processing; many reports did not name the atlas, registration software/version, transform class, interpolation kernel, or lesion-masking strategy. Nonlinear registration to a healthy adult atlas can deform tumors, infarcts, or age-dependent anatomy differently across sites; differing voxel grids and interpolation alter lesion volume and texture, while a study-specific template constructed before splitting can transmit test-cohort anatomy into training. A model trained on outputs from one ANTs, FSL, SPM, or FreeSurfer configuration may therefore encode pipeline signatures rather than portable biology (Thakur et al., 2020; Tustison et al., 2021; Billot et al., 2023).

To limit compounded domain shift, registration, template construction, intensity normalization, and harmonization should be fitted using training data only; the atlas and transform parameters should then be frozen for validation. Studies should compare at least one native-space or alternative-atlas analysis, report interpolation and resampling explicitly, quantify registration failure by disease and site, and test whether harmonization preserves subgroup and lesion-size effects (Radua et al., 2020; Dinsdale et al., 2021).

### 3.8 Evaluation and validation strategies: RQ4

Classification studies most frequently reported accuracy, sensitivity, specificity, precision, recall, F1-score, and area under the receiver operating characteristic curve (AUC). AUC was useful under varying thresholds but did not communicate calibration or clinical consequences. Accuracy was misleading in imbalanced detection problems. Precision-recall analysis was more informative when positive lesions or events were rare.

Segmentation studies reported Dice similarity coefficient, intersection over union, lesion-wise sensitivity, false positives, and Hausdorff distance. Dice favors larger structures and may conceal missed small lesions. Boundary metrics capture spatial error but can be unstable when either mask is empty. Lesion-level detection, volume agreement, and clinically defined regional error therefore complemented overlap metrics.

Hold-out and k-fold cross-validation were frequently reported, but many articles did not clearly state whether splitting occurred by patient, scan, slice, or site. Patient-level partitioning is a minimum leakage safeguard. Nested validation addresses optimization bias when features or hyperparameters are selected, while site-held-out, temporal, external, prospective, and reader evaluations address different dimensions of transportability and workflow evidence. These designs are complementary rather than a simple universal hierarchy.

Examples with comparatively stronger transportability evidence explicitly tested independent centers. Interpretable Alzheimer classification was evaluated in AIBL, Framingham, and NACC after ADNI development (Qiu S. et al., 2020). Glioma IDH prediction was evaluated in separate hospital and TCIA cohorts (Choi et al., 2021). Parkinsonian classification was replicated on clinical scanners that differed from the research development environment (Chougar et al., 2021). Focal cortical dysplasia detection used leave-one-site-out and independent cohorts (Gill et al., 2021). These examples support cautious claims about specific settings, not general architecture rankings.

Direct performance comparison remains inappropriate when reference standards, prevalence, or cohorts differ. An AUC from a balanced public benchmark cannot be interpreted as equivalent to the same AUC in an emergency workflow. Similarly, a high Dice score for a large tumor cannot be compared directly with lower Dice for small cortical MS or epilepsy lesions. Evaluation should be tied to intended use and error consequences.

### 3.9 Quality assessment and risk of bias

Patient and data selection was the most frequent source of applicability concern. Curated case–control cohorts often excluded comorbidity, poor image quality, uncertain diagnosis, and incomplete modalities. These exclusions improve technical clarity but make deployment populations more difficult. Small samples combined with high-dimensional inputs increased overfitting risk, particularly when multiple architectures were tested.

Reference standards varied from expert segmentation and pathology to clinical diagnosis or follow-up. Pathology-confirmed tumor labels and multicenter expert lesion masks offered relatively strong standards. Clinical dementia labels without biomarker confirmation were more vulnerable to etiological overlap. EEG annotation depended on reader agreement, and subtle MRI-negative epilepsy lesions remained difficult even for experts.

Leakage concerns included slice-wise partitioning, augmentation before splitting, feature selection on the full dataset, harmonization across all sites before validation, and repeated test-set tuning. Reproducible Alzheimer experiments identified potential leakage in more than half of surveyed CNN studies (Wen et al., 2020). Clear patient-level split reporting was therefore treated as a core quality requirement rather than a minor technical detail.

This audit separates optimization bias from downstream transportability. A study can use a simple internal hold-out and also test an external cohort; external testing strengthens evidence about transfer but does not repair feature-selection leakage, repeated test querying, or other optimization bias in the development pipeline.

External validation and calibration were infrequently documented in the retained extraction. Many studies reported discrimination without confidence intervals, calibration curves, decision analysis, or uncertainty. Code and model release were inconsistent. Some recent reports showed more complete CLAIM- or TRIPOD+AI-aligned disclosure, but the evidence file did not support a formal year-by-year reporting comparison.

### 3.10 Methodological and translational challenges: RQ5

Data-related challenges included limited annotated cohorts, class imbalance, scanner heterogeneity, domain shift, inconsistent labels, missing modalities, and restricted data sharing. Algorithmic challenges included overfitting, unstable explanations, high computational cost, shortcut learning, adversarial or acquisition sensitivity, and difficulty combining three-dimensional, temporal, and multimodal information. Evaluation challenges included inconsistent metrics, weak comparator design, absent external testing, and non-reproducible preprocessing (see Table 6).

**Table 6:** Key challenges, research gaps, and future directions in AI-assisted brain imaging.

Category	Identified challenge	Consequence for diagnosis	Recommended direction	Research priority
Data	Small annotated cohorts	Overfitting and unstable subgroup estimates	Multicenter curation, label uncertainty, low-label learning	Very high (Vasey et al., 2022; Mongan et al., 2020; Moons et al., 2025; Page et al., 2021; Thompson et al., 2020)
Data	Scanner/protocol heterogeneity	Domain shift and site shortcuts	Training-only harmonization; site-held-out testing	Very high (Carré et al., 2020; Billot et al., 2023; Mårtensson et al., 2020; Radua et al., 2020; Ingalhalikar et al., 2021; Dinsdale et al., 2021; Kamraoui et al., 2022)
Data	Class and lesion-size imbalance	Missed rare classes and small lesions	Task-aware sampling, calibrated losses, lesion-wise metrics	High (Gros et al., 2021; La Rosa et al., 2020; Wiltgen et al., 2024)
Data	Annotation and reference uncertainty	Noisy labels and artificial performance ceilings	Multi-reader consensus, pathology/follow-up, probabilistic labels	Very high (Fürbass et al., 2020; Spitzer et al., 2022; Kural et al., 2022; Gill et al., 2021)
Methods	Patient or preprocessing leakage	Optimistic internal performance	Patient-level splitting, nested selection, pipeline audit	Very high (Collins et al., 2024; Mongan et al., 2020; Wen et al., 2020; Moons et al., 2025)
Methods	Missing multimodal inputs	Complete-case bias and limited deployability	Modality dropout, masked fusion, explicit missingness models	High (Xue et al., 2024; Conte et al., 2021; Venugopalan et al., 2021; Qiu et al., 2022; Zhang Y. D. et al., 2020)
Methods	Unfaithful or unstable explanations	Misplaced clinical trust	Stability, perturbation, counterfactual, clinician evaluation	High (Yin et al., 2023; Kohoutová et al., 2020; Magesh et al., 2020; Qiu S. et al., 2020; El-Sappagh et al., 2021)
Methods	Unquantified uncertainty	Overconfident errors and unsafe automation	Calibration, prediction sets, abstention, drift monitoring	Very high (Tanno et al., 2021; Gill et al., 2021)
Evaluation	Inconsistent metrics and thresholds	Poor comparability and unclear clinical meaning	Task-specific core metrics plus confidence intervals	High (Collins et al., 2024; Mongan et al., 2020; Moons et al., 2025)
Evaluation	Limited external validation	Unknown geographic and scanner transfer	Independent, temporal, site-held-out, and prospective tests	Very high (Vasey et al., 2022; Mårtensson et al., 2020; Collins et al., 2024; Moons et al., 2025; Chougar et al., 2021; Gill et al., 2021; Matsoukas et al., 2023; Choi et al., 2021)
Reproducibility	Unavailable code, weights, and exact pipelines	Results cannot be independently reproduced	Open code, containers, versioned data dictionaries	High (Billot et al., 2023; Mongan et al., 2020; Henschel et al., 2020; Muckley et al., 2021; Tustison et al., 2021; Mei et al., 2022; Zhou et al., 2021)
Fairness	Underrepresented demographic and disease groups	Unequal error rates and restricted applicability	Subgroup reporting, representative sampling, fairness audits	Very high (Solana-Lavalle and Rosas-Romero, 2021; Beheshti et al., 2020; Camacho et al., 2023; Thompson et al., 2020)
Governance	Privacy and data-sharing restrictions	Fragmented development data	Federated analysis, secure enclaves, auditable governance	High (Vasey et al., 2022; Collins et al., 2024; Moons et al., 2025; Thompson et al., 2020)
Translation	Workflow mismatch and alert burden	Low adoption or delayed care	Human factors, interoperability, reader and workflow trials	Very high (Vasey et al., 2022; Martinez-Gutierrez et al., 2023; Elijovich et al., 2022; Seyam et al., 2022; Matsoukas et al., 2023)
Translation	Regulation, maintenance, and model drift	Unsafe performance decay after deployment	Lifecycle monitoring, version control, predefined update policy	Very high (Vasey et al., 2022; Collins et al., 2024; Mongan et al., 2020; Moons et al., 2025)

Selected studies described feasible responses to recurrent limitations. Multicenter consortia increased diversity; harmonization and site-held-out tests assessed scanner effects; uncertainty and abstention identified some low-confidence cases; open code and structured data descriptions supported reproducibility; and prospective or reader studies evaluated workflow questions. The heterogeneous evidence does not establish that any single response is sufficient.

## 4 Discussion

### 4.1 Summary of main findings

The reviewed literature suggests movement from isolated pattern-recognition experiments toward more integrated diagnostic systems in selected settings, while methodological maturity remained uneven. MRI was frequently represented because it can encode anatomy, lesion contrast, diffusion, perfusion, and functional connectivity. CT had comparatively direct workflow evidence in selected stroke and hemorrhage studies because acquisition is rapid and time-to-decision is measurable. PET and SPECT contributed disease-specific molecular information, and EEG extended the evidence base to time-resolved functional diagnosis. These observations are descriptive and should not be interpreted as a ranking of modalities.

RQ1 identified a descriptive shift from handcrafted radiomics and conventional machine learning toward CNNs, three-dimensional segmentation, attention, Transformers, GNNs, self-supervised learning, and multimodal systems. CNNs were frequently represented, while Transformer and graph methods showed promise in selected studies, especially when combined with convolutional or neurobiological priors. Differences in cohort size, task, pretraining, and validation prevent a general claim that newer architectures are superior.

RQ2 suggested that modality shapes both available information and likely failure modes. MRI studies faced sequence and scanner shift; CT studies faced subtle-lesion and reconstruction variability; PET studies faced tracer and sample-size constraints; and fMRI/DTI studies faced preprocessing and network-definition sensitivity. The disease-modality map is a descriptive co-occurrence analysis, not evidence that one modality is inherently optimal. Multimodal systems showed promise in selected cohorts, but missing-data handling and acquisition feasibility were often incompletely reported.

RQ3 found that stroke, dementia, and brain tumors were frequently represented in the retained coding. Large public datasets, defined imaging targets, and clinical demand may partly explain this pattern, but the review did not test these explanations statistically. Parkinson disease, epilepsy, and MS included multimodal, network, or longitudinal studies, while less common neurological conditions were less visible in the reviewed evidence.

RQ4 documented substantial variation in datasets, preprocessing, validation, and metrics. Public benchmarks supported reproducible comparison within defined tasks but also risked repeated benchmark adaptation. Institutional cohorts increased clinical realism in selected studies but often lacked independently extractable testing details. Because complete row-level validation codes were not available, the review now reports validation patterns qualitatively and does not estimate the prevalence of nested, patient-level, external, or prospective designs.

RQ5 identified linked concerns in data, validation, reporting, governance, and workflow design. The corrected synthesis no longer uses untraceable aggregate validation counts as evidence of clinical readiness. Instead, it emphasizes the study-level fields that were recoverable, marks unavailable data as NR, and treats incomplete extraction as a review limitation rather than as evidence that a safeguard was absent.

### 4.2 Evolution from conventional machine learning to advanced AI

The transition from handcrafted features to learned representations changed what can be modeled. Traditional radiomics compresses an image into predefined intensity, texture, shape, or regional measurements. This facilitates interpretation and small-sample analysis but may discard spatial relationships. CNNs learn local hierarchical representations directly from images, while three-dimensional networks preserve volumetric context. Attention and Transformers model long-range relationships, and GNNs represent explicit brain networks. Multimodal and self-supervised learning further reduce the distinction between image processing and clinical representation learning.

This evolution does not imply that every later method is superior. ROI-level epilepsy analysis found similar or occasionally better performance for SVM than deep learning (Gleichgerrcht et al., 2021). Reproducible Alzheimer classification likewise showed that rigorous three-dimensional CNNs did not necessarily surpass an SVM baseline (Wen et al., 2020). Such comparisons reveal a recurring principle: stronger inductive bias and simpler hypothesis classes may outperform high-capacity models when cohort size, label quality, or signal strength is limited.

The strongest deep systems used scale, external validation, or explicit clinical design to justify complexity. Interpretable Alzheimer classification combined MRI and clinical variables across independent cohorts (Qiu S. et al., 2020). Multicenter T1 MRI supported explainable Parkinson classification beyond a single repository (Camacho et al., 2023). Large routine-EEG datasets enabled comprehensive abnormality classification instead of a narrow event detector (Tveit et al., 2023). These examples suggest that advanced architectures become valuable when the evidence base expands proportionally.

Representation learning also altered preprocessing. Deep segmentation and registration pipelines can standardize inputs, but they introduce dependencies on their own training distributions. Brain extraction in the presence of diffuse glioma demonstrated that preprocessing failure can propagate into every downstream task (Thakur et al., 2020). Synthetic double-inversion-recovery MRI improved MS lesion visibility, but generated contrasts require clinical evaluation and careful disclosure (Finck et al., 2020). AI-generated DIR and PSIR were later assessed across centers for cortical and juxtacortical lesion detection (Bouman et al., 2023).

Self-supervision and pretraining address label scarcity but introduce transfer questions. Models Genesis learned generic three-dimensional representations from medical images (Zhou et al., 2021). RadImageNet provided radiology-focused supervised pretraining (Mei et al., 2022). Physics-guided reconstruction learned without fully sampled targets (Yaman et al., 2020). Future neurological pretraining should compare natural-image, radiological, modality-specific, and disease-specific initialization under the same downstream protocol.

### 4.3 Multimodal AI and structural-functional integration

The scientific rationale for multimodal AI is strong because neurological disease affects several biological scales. Structural MRI captures atrophy and lesions; fMRI captures functional organization; DTI captures microstructure and tracts; PET captures metabolism or molecular pathology; EEG and MEG capture rapid neural dynamics; and clinical information describes symptoms, cognition, and context. Integration can therefore reduce ambiguity that no single source can resolve.

Dementia offers the clearest example. Multimodal deep learning combined MRI, genetics, and clinical testing for stage classification (Venugopalan et al., 2021). A flexible framework incorporated clinical history, neuropsychology, functional assessments, and neuroimaging for differential dementia assessment (Qiu et al., 2022). Interpretable PET decomposition in 2026 sought to separate pathological signals with greater clinical relevance (Tang et al., 2026). Nevertheless, multimodal models can learn site-specific missingness or referral patterns rather than pathology, especially when complete examinations are available only for selected patients.

Moving beyond macroscopic fusion will require biologically constrained cross-scale models. Precision genome engineering and CRISPR-based screening can identify causal gene-regulatory perturbations and supply pathway priors, rather than serving as clinical imaging inputs themselves (Huang K. et al., 2026). In Alzheimer disease, mitochondrial homeostasis dysregulation and TDP-43-associated impairment of mitochondrial dynamics, transport, quality control, and stress responses provide a plausible subcellular layer that imaging alone cannot resolve (Huang W. et al., 2026). Future frameworks could combine MRI/PET-derived phenotypes with polygenic scores, transcriptomic or cell-type annotations, circulating mitochondrial markers, and longitudinal cognition through pathway-informed graphs or multimodal latent-variable models. Such integration should be tested for incremental prediction, temporal precedence, calibration, and transport across ancestry and tissue-assay platforms; otherwise molecular features may add dimensionality without causal or clinical value. These two post-cutoff sources define a translational research agenda and are not treated as evidence within the 123-study synthesis.

Parkinson and atypical parkinsonism also benefited from modality complementarity. Multimodal MRI improved differentiation of Parkinson disease and progressive supranuclear palsy (Talai et al., 2021). PET/MRI radiomics combined metabolism, dopamine-transporter information, and anatomy (Sun et al., 2024). Clinical and DAT SPECT features supported prediction of cognitive decline (Hosseinzadeh et al., 2023). These studies indicate that fusion is most useful when the diagnostic alternatives are biologically related but express distinct structural or molecular patterns.

Structural-functional integration is especially important for network disorders. Resting-state fMRI classification of Alzheimer disease can capture changes not visible in structural MRI, but smaller samples and complex preprocessing make external validation difficult (Duc et al., 2020). Temporal-lobe epilepsy connectome contraction related structural microarchitecture to surgical outcome (Larivière et al., 2020). ASD graph learning integrated multiple functional views with anatomical priors (Wen et al., 2022). The main limitation is that connectomes are constructed objects whose nodes and edges depend on acquisition, preprocessing, parcellation, and thresholding.

Future multimodal systems should report per-modality ablation, missingness patterns, acquisition timing, cost, and incremental clinical value. A model that requires PET, high-resolution MRI, neuropsychology, genetics, and complete follow-up may achieve high retrospective accuracy while serving only a small, selected population. Clinically useful fusion should tolerate absent modalities, express uncertainty when information is incomplete, and identify the minimal sufficient examination for each decision.

Selected independent-cohort dementia studies provided more direct translational evidence for flexible, variable-subset fusion than the mainly in-distribution synthesis studies available in the retained evidence set (Qiu et al., 2022; Xue et al., 2024). This pattern suggests that modality masking, dropout, or set-based encoders may be useful when missingness is expected, but it does not establish general superiority over generative imputation because matched head-to-head, unseen-site comparisons were scarce. GAN-based replacement of missing MRI contrasts was often evaluated using image fidelity or downstream segmentation (Conte et al., 2021; Jayachandran Preetha et al., 2021), and scanner-specific hallucination remained a concern. Future comparisons should model random, workflow-conditional, and clinically informative missingness on identical partitions, report every clinically common modality subset, and propagate imputation uncertainty.

### 4.4 Explainability, reliability, and trustworthy AI

Explainability was frequently invoked but inconsistently evaluated. Saliency maps, class-activation maps, feature importance, attention weights, and local surrogate explanations can reveal associations between inputs and outputs. LIME-based DaTSCAN analysis attempted to expose image regions supporting Parkinson predictions (Magesh et al., 2020). Anatomically interpretable brain-age learning connected deviations to domain-specific cognitive impairment (Yin et al., 2023). Deep structural representations of Alzheimer disease identified continuous dimensions rather than only categorical labels (Yang et al., 2021).

Visual plausibility is not sufficient evidence of faithfulness. Explanations can change under small input perturbations, model reinitialization, or equivalent predictions. They may highlight large anatomical structures even when the classifier uses scanner artifacts elsewhere. Interpretation should therefore include stability tests, deletion or insertion experiments, counterfactual analysis, agreement with independent biomarkers, and blinded clinician evaluation. Neuroimaging interpretation frameworks explicitly separate model explanation from causal inference about disease biology (Kohoutová et al., 2020).

Reliability also requires uncertainty and calibration. Bayesian uncertainty supported confidence ranking in focal cortical dysplasia detection (Gill et al., 2021). Uncertainty-aware diffusion enhancement demonstrated that error maps can accompany reconstructed neuroimages (Tanno et al., 2021). Calibration is particularly important when prevalence changes between benchmark and clinic. A well-calibrated lower-AUC model may be safer than a higher-AUC model that produces overconfident false negatives.

Trustworthy AI includes robustness, fairness, privacy, security, and accountability. Scanner, sex, age, ethnicity, socioeconomic access, and disease severity can all become sources of unequal performance. Sex-stratified Parkinson experiments identified performance differences but did not replace intersectional evaluation (Solana-Lavalle and Rosas-Romero, 2021). Brain-age models spanning Alzheimer and Parkinson disease illustrate how demographic correction influences disease interpretation (Beheshti et al., 2020). Fairness analysis should report subgroup sample sizes and uncertainty rather than only point estimates.

Reporting standards provide a practical bridge between technical and clinical trust. CLAIM specifies information needed for medical-imaging AI reports (Mongan et al., 2020). TRIPOD+AI updates reporting for prediction models that use regression or machine learning (Collins et al., 2024). PROBAST+AI supports structured appraisal of development and evaluation bias (Moons et al., 2025). DECIDE-AI focuses on early-stage clinical evaluation of AI decision support (Vasey et al., 2022). Adherence will not eliminate bias, but it makes limitations detectable and studies reproducible.

To operationalize these standards, Table 7 proposes a minimum reporting checklist tailored to neuroimaging AI. It emphasizes cohort composition, exact partitioning units, leakage-safe model selection, task-appropriate statistical evidence, and reproducible preprocessing.

**Table 7:** Minimum reporting checklist for neuroimaging AI studies.

Reporting domain	Minimum disclosure	Minimum evidence or safeguard
Cohort provenance and flow	Sites, recruitment dates, inclusion/exclusion criteria, participant/examination flow, exclusions after quality control, and numbers in every split.	Flow diagram or auditable cohort table; no unexplained loss between source and analysis sets.
Demographic and clinical distribution	Age (distribution and range), sex/gender, race/ethnicity where lawful and relevant, disease subtype/severity, comorbidity, and access-related variables, reported overall and by split.	Counts and uncertainty for clinically important subgroups; differences between development and test cohorts.
Acquisition metadata	Scanner vendor/model, field strength, coil, sequence or reconstruction parameters, voxel size, contrast/tracer, protocol changes, and site-specific deviations.	Sufficient detail to reproduce acquisition strata and analyze scanner/protocol shift.
Preprocessing and spatial normalization	Software and version; atlas/template; registration transform; interpolation/resampling; skull stripping; bias/intensity correction; lesion masking; harmonization; and the partition in which each step was fitted.	Frozen pipeline for validation; registration failures and atlas sensitivity reported by site/disease where relevant.
Reference standard	Label source, annotator number/expertise, blinding, adjudication/consensus, pathology or follow-up confirmation, inter-reader agreement, and uncertainty handling.	Reference-standard limitations linked to the intended clinical use.
Partitioning unit and provenance	Explicit patient-level separation; handling of repeated scans, slices, patches, relatives, augmented samples, sites, and time; exact train/validation/test provenance.	No patient or derivative crossing partitions; site/time hold-out reported when claimed.
Model selection and test isolation	Nested cross-validation or a separate development-validation process; feature selection, hyperparameters, early stopping, calibration, thresholds, random seeds, and number of test queries.	Outer performance estimation isolated from every optimization decision.
Missingness and multimodal availability	Per-modality completeness; plausible missingness mechanism; missingness indicators; imputation, masking, or modality-dropout procedure; and performance for clinically common modality subsets.	Uncertainty propagated when synthetic or imputed inputs are used.
Task-specific metrics and statistics	Classification: sensitivity, specificity, ROC-AUC/PR-AUC, calibration, threshold rule. Segmentation: Dice, lesion-wise recall/false positives, boundary error/HD95, and lesion-size strata. Confidence intervals for principal estimates.	Metrics matched to intended use, prevalence, and error consequences; statistical comparison plan stated.
External and out-of-distribution validation	Unseen site, scanner, geography, time period, or prospective cohort; distribution differences; locked model; and any recalibration.	Internal and external results separated; performance degradation and failure modes quantified.
Fairness, robustness, and failure analysis	Subgroup sample sizes/performance with uncertainty; small-lesion and rare-class results; artifacts, missing sequences, registration failures, and abstention behavior.	Worst-group and clinically consequential errors described, not only overall averages.
Reproducibility and lifecycle	Code/weights/configuration, data identifiers, computing environment, inference time, model/version card, intended use, exclusions, drift monitoring, and update governance.	Independent reproduction or an explicit reason for restricted release; predefined maintenance plan for deployed systems.

Table 7 is a minimum set rather than a substitute for CLAIM, TRIPOD+AI, PROBAST+AI, or DECIDE-AI; task- and deployment-specific disclosures should be added when relevant.

### 4.5 Generalizability and clinical translation

Independent external validation supplied more relevant evidence about transportability than internal resampling alone, although it did not guarantee clinical utility. Multicenter focal cortical dysplasia detection, clinical-scanner replication of parkinsonian classification, and external glioma IDH evaluation each showed setting-specific changes in performance (Gill et al., 2021; Chougar et al., 2021; Choi et al., 2021). These studies support cautious conclusions for their tested populations and cannot be generalized across modalities or diseases.

Generalizability requires deliberate validation units. Random patient splits test interpolation within a combined distribution. Site-held-out splits test acquisition and population shift. Temporal validation tests changes in clinical practice. External geographic testing assesses transfer to new institutions. Prospective evaluation additionally captures workflow, prevalence, and human response. Reader studies assess whether AI complements rather than duplicates expertise.

Stroke provides early evidence across this hierarchy. Real-world prospective CT angiography testing showed high proximal-occlusion sensitivity but poorer M2 detection (Matsoukas et al., 2023). Hub-and-spoke deployment improved workflow measures in observational evaluation (Elijovich et al., 2022). Cluster-randomized implementation reduced door-to-groin and imaging-to-treatment intervals (Martinez-Gutierrez et al., 2023). These studies move beyond image accuracy, although patient-centered outcome benefit remains harder to demonstrate.

Regulatory and maintenance requirements continue after initial deployment. Scanner upgrades, protocol changes, disease prevalence, referral criteria, and clinician behavior can alter performance. Models should be monitored for drift, recalibrated under governance, and versioned with auditable data. Continual learning without safeguards risks changing clinical behavior unpredictably. Federated or distributed development may broaden data access, but it does not automatically solve label inconsistency or site imbalance.

Clinical endpoints should match intended use. A segmentation model may be useful if it reduces reporting time or improves lesion-burden reproducibility even when Dice improvement is small. A triage model should be evaluated for sensitivity, false-alert burden, and time to specialist review. A differential-diagnosis model should be tested for incremental benefit over clinicians and standard imaging. A progression model should report calibration and decision consequences. Translation depends on whether output changes a clinically meaningful decision safely.

### 4.6 Future research directions

The first priority is large, diverse, well-annotated, and transparently governed multicenter data. Disease-specific consortia such as ENIGMA-Epilepsy demonstrate the value of coordinated scale (Sisodiya et al., 2020). Public challenge construction in intracranial hemorrhage shows how shared definitions and collaborative labeling can support reproducible development (Flanders et al., 2020). Future repositories should include scanner metadata, longitudinal follow-up, demographic diversity, annotation uncertainty, and clearly defined access conditions.

The second priority is harmonization without leakage. Intensity standardization, ComBat, domain adaptation, and representation unlearning should be fitted within training partitions and evaluated on unseen sites. Site classifiers can quantify residual acquisition information. Harmonization should preserve disease-relevant population variation, and studies should report whether adjustment changes subgroup performance.

The third priority is efficient self-supervised and foundation-model learning adapted to three-dimensional and multimodal neuroimaging. Pretraining objectives should preserve small lesions, quantitative intensity, and anatomical correspondence. Evaluation should include linear probing, full fine-tuning, low-label regimes, external sites, and calibration. General-purpose segmentation models should be compared with specialized U-Net baselines under identical data and annotation conditions.

The fourth priority is multimodal learning that handles missing data and estimates incremental value. Models should distinguish absence caused by clinical workflow from random missingness. Modality dropout, product-of-experts formulations, masked multimodal pretraining, and uncertainty-aware fusion are promising. Reporting should include performance for realistic subsets rather than only complete multimodal cases.

The fifth priority is explanation linked to reliability. Region maps should be tested for stability and faithfulness. Counterfactuals should remain anatomically plausible. Prediction sets, calibrated probabilities, and abstention policies should communicate uncertainty. Human-AI studies should evaluate whether explanations improve error detection or simply increase automation bias.

The sixth priority is prospective, multicenter, and workflow-aware evaluation. The objective is not merely to reproduce retrospective AUC, but to determine effect on diagnostic time, reader agreement, unnecessary imaging, missed disease, downstream testing, and patient outcomes. Adaptive monitoring plans should be established before deployment. Data and model cards should document intended use, exclusions, known failure modes, and update policy.

### 4.7 Strengths and limitations of the systematic review

This review integrates structural, functional, metabolic, diffusion, tomographic, and electrophysiological modalities across major neurological diseases. Five linked RQs connect architecture with modality, task, data, evaluation, and translation. The search spans biomedical, multidisciplinary, engineering, and publisher-platform sources, and Appendix A reports full platform-specific syntax. The re-audit distinguishes the corrected 123-study evidence set from 21 contextual references and auxiliary multi-label visualizations. Supplementary Table S0 supplies the 137-record eligibility crosswalk, Supplementary Table S1 provides recoverable study-level fields for the 123 eligible studies, and Supplementary Tables S2, S3 define coding and corrected screening totals.

The review also has important limitations. All screening, selection, extraction, and quality appraisal were performed by one author, with same-assessor verification but no independent second assessor or inter-rater agreement. Restriction to English-language peer-reviewed literature may introduce language and publication bias. Embase was omitted because institutional access was unavailable; coverage by PubMed/MEDLINE, CINAHL, Scopus, Web of Science, and citation tracking is not an equivalent substitute. The protocol was not prospectively registered. Item-level title/abstract decisions and the full-text exclusion list were not deposited in a public repository for this revision. These limitations increase the possibility of missed studies, extraction error, and subjective quality judgments.

Primary-study reporting was often incomplete. Sample selection, excluded images, patient-level partitioning, calibration, and external testing were not consistently documented. Supplementary Table S1 therefore marks unavailable retained-extraction values as NR instead of inferring them. Heterogeneity in populations, imaging protocols, labels, tasks, outcomes, and validation quality precluded meta-analysis and limits comparative conclusions. Findings concerning Transformers, foundation models, modality dominance, and clinical maturity should be treated as descriptive or hypothesis-generating. The search closed in June 2026, and later evidence is outside scope.

The remaining quantitative graphics use explicit but different denominators. Figures 2–4 describe an auxiliary publication-mapping set or multi-label coding events and do not use the 123-study PRISMA denominator. None of the remaining descriptive graphics supports a causal claim or a universal ranking of architectures, diseases, modalities, or validation strategies.

## 5 Conclusion

The reviewed evidence indicates that AI methods are widely used in brain-image analysis and neurological CAD, but the strength and clinical relevance of evidence vary substantially. RQ1 identified use of handcrafted machine learning, volumetric CNNs, attention, Transformers, graph models, self-supervised learning, generative methods, and multimodal systems without establishing a universal architecture ranking. RQ2 found MRI frequently represented in descriptive coding, while CT, PET/SPECT, fMRI, diffusion, EEG, and MEG supported different selected tasks and populations. RQ3 found stroke, dementia, and brain tumors frequently represented, with classification, detection, segmentation, localization, differential diagnosis, grading, and staging among reported tasks.

RQ4 documented heterogeneous datasets, preprocessing, validation units, and metrics. The retained extraction was insufficient for reproducible aggregate frequencies of nested, external, patient-level, or prospective validation, so those prior percentages were withdrawn. RQ5 identified scanner domain shift, annotation uncertainty, imbalance, data leakage, incomplete reporting, unstable explanation, limited fairness analysis, and weak workflow evidence as recurrent concerns.

Future work should prioritize multicenter data governance, leakage-safe harmonization, transparent and task-matched model comparison, missingness-aware multimodal fusion, uncertainty and calibration, reproducible pipelines, and prospective human-AI evaluation. Advanced models showed promise in selected studies for quantifying abnormalities or integrating complex evidence, but heterogeneous retrospective results do not establish dependable clinical diagnosis. Generalizability, interpretability, reproducibility, and clinically meaningful validation remain necessary conditions for stronger translational claims.

## Data Availability

The original contributions presented in the study are included in the article/Supplementary material, further inquiries can be directed to the corresponding author.
