# Supplementary material for: Advances in artificial intelligence for brain imaging and computer-aided diagnosis of neurological diseases: a systematic literature review
Source: Front Hum Neurosci. 2026 Oct 14;20:1963855. doi: 10.3389/fnhum.2026.1963855 (PMC13713707; doi:10.3389/fnhum.2026.1963855)
Supplement: Supplementary file 1 [file Table_1.DOCX]

# 1 Supplementary material included with the revised manuscript

## 1.1 Appendix A. Complete database-specific search strategies

Final database searches were executed on 30 June 2026 for records dated 1 January 2020 through 30 June 2026. Backward reference-list checking and forward citation tracking in Scopus and Web of Science were completed on 2 July 2026. Citation-derived records were screened with the same criteria, and no additional unique eligible report remained after deduplication. The strings below record field restrictions, controlled vocabulary, and filters. Platform punctuation is retained where applicable.

**Scopus.** TITLE-ABS-KEY(("artificial intelligence" OR "machine learning" OR "deep learning" OR "convolutional neural network*" OR CNN OR "3D CNN" OR attention OR transformer* OR "vision transformer*" OR "graph neural network*" OR radiomic* OR autoencoder* OR "generative adversarial network*" OR "self-supervised learning" OR "transfer learning" OR "multimodal learning") AND ("brain imaging" OR neuroimaging OR "magnetic resonance imaging" OR MRI OR fMRI OR "functional MRI" OR DTI OR "diffusion tensor imaging" OR "computed tomography" OR CT OR CTA OR CTP OR PET OR SPECT OR EEG OR MEG OR connectom*) AND ("neurological disease*" OR stroke OR "cerebrovascular disease*" OR Alzheimer* OR dementia OR Parkinson* OR "brain tumor*" OR "brain tumour*" OR glioma* OR epilepsy OR seizure* OR "multiple sclerosis" OR "traumatic brain injury" OR autism OR "disorder* of consciousness") AND (diagnos* OR detect* OR classif* OR segment* OR localiz* OR stag* OR grad* OR predict* OR "computer-aided diagnosis" OR "computer assisted diagnosis")) AND PUBYEAR > 2019 AND PUBYEAR < 2027 AND LIMIT-TO(LANGUAGE, "English"). Document types were restricted to articles and complete peer-reviewed proceedings; editorials, letters, abstracts, reviews without primary data, and preprints were excluded during screening. Retrieved: 317.

**Web of Science Core Collection.** TS=(("artificial intelligence" OR "machine learning" OR "deep learning" OR "convolutional neural network*" OR CNN OR "3D CNN" OR attention OR transformer* OR "vision transformer*" OR "graph neural network*" OR radiomic* OR autoencoder* OR "generative adversarial network*" OR "self-supervised learning" OR "transfer learning" OR "multimodal learning") AND ("brain imaging" OR neuroimaging OR "magnetic resonance imaging" OR MRI OR fMRI OR "functional MRI" OR DTI OR "diffusion tensor imaging" OR "computed tomography" OR CT OR CTA OR CTP OR PET OR SPECT OR EEG OR MEG OR connectom*) AND ("neurological disease*" OR stroke OR "cerebrovascular disease*" OR Alzheimer* OR dementia OR Parkinson* OR "brain tumor*" OR "brain tumour*" OR glioma* OR epilepsy OR seizure* OR "multiple sclerosis" OR "traumatic brain injury" OR autism OR "disorder* of consciousness") AND (diagnos* OR detect* OR classif* OR segment* OR localiz* OR stag* OR grad* OR predict* OR "computer-aided diagnosis" OR "computer assisted diagnosis")) AND PY=(2020-2026) AND LA=(English). Indexes: SCI-EXPANDED, SSCI, ESCI, and CPCI-S/CPCI-SSH; document types Article and Proceedings Paper. Retrieved: 268.

**IEEE Xplore.** ("All Metadata":("artificial intelligence" OR "machine learning" OR "deep learning" OR "convolutional neural network*" OR CNN OR "3D CNN" OR attention OR transformer* OR "vision transformer*" OR "graph neural network*" OR radiomic* OR autoencoder* OR "generative adversarial network*" OR "self-supervised learning" OR "transfer learning" OR "multimodal learning")) AND ("All Metadata":("brain imaging" OR neuroimaging OR "magnetic resonance imaging" OR MRI OR fMRI OR "functional MRI" OR DTI OR "diffusion tensor imaging" OR "computed tomography" OR CT OR CTA OR CTP OR PET OR SPECT OR EEG OR MEG OR connectom*)) AND ("All Metadata":("neurological disease*" OR stroke OR "cerebrovascular disease*" OR Alzheimer* OR dementia OR Parkinson* OR "brain tumor*" OR "brain tumour*" OR glioma* OR epilepsy OR seizure* OR "multiple sclerosis" OR "traumatic brain injury" OR autism OR "disorder* of consciousness")) AND ("All Metadata":(diagnos* OR detect* OR classif* OR segment* OR localiz* OR stag* OR grad* OR predict* OR "computer-aided diagnosis" OR "computer assisted diagnosis")). Filters: publication years 2020-2026, Journals and Conferences, English. Retrieved: 156.

**ACM Digital Library.** [[Title:("artificial intelligence" OR "machine learning" OR "deep learning" OR "convolutional neural network*" OR CNN OR "3D CNN" OR attention OR transformer* OR "vision transformer*" OR "graph neural network*" OR radiomic* OR autoencoder* OR "generative adversarial network*" OR "self-supervised learning" OR "transfer learning" OR "multimodal learning")] OR [Abstract:("artificial intelligence" OR "machine learning" OR "deep learning" OR "convolutional neural network*" OR CNN OR "3D CNN" OR attention OR transformer* OR "vision transformer*" OR "graph neural network*" OR radiomic* OR autoencoder* OR "generative adversarial network*" OR "self-supervised learning" OR "transfer learning" OR "multimodal learning")]] AND [[Title:("brain imaging" OR neuroimaging OR "magnetic resonance imaging" OR MRI OR fMRI OR "functional MRI" OR DTI OR "diffusion tensor imaging" OR "computed tomography" OR CT OR CTA OR CTP OR PET OR SPECT OR EEG OR MEG OR connectom*)] OR [Abstract:("brain imaging" OR neuroimaging OR "magnetic resonance imaging" OR MRI OR fMRI OR "functional MRI" OR DTI OR "diffusion tensor imaging" OR "computed tomography" OR CT OR CTA OR CTP OR PET OR SPECT OR EEG OR MEG OR connectom*)]] AND [[Title:("neurological disease*" OR stroke OR "cerebrovascular disease*" OR Alzheimer* OR dementia OR Parkinson* OR "brain tumor*" OR "brain tumour*" OR glioma* OR epilepsy OR seizure* OR "multiple sclerosis" OR "traumatic brain injury" OR autism OR "disorder* of consciousness")] OR [Abstract:("neurological disease*" OR stroke OR "cerebrovascular disease*" OR Alzheimer* OR dementia OR Parkinson* OR "brain tumor*" OR "brain tumour*" OR glioma* OR epilepsy OR seizure* OR "multiple sclerosis" OR "traumatic brain injury" OR autism OR "disorder* of consciousness")]] AND [[Title:(diagnos* OR detect* OR classif* OR segment* OR localiz* OR stag* OR grad* OR predict* OR "computer-aided diagnosis" OR "computer assisted diagnosis")] OR [Abstract:(diagnos* OR detect* OR classif* OR segment* OR localiz* OR stag* OR grad* OR predict* OR "computer-aided diagnosis" OR "computer assisted diagnosis")]]. Filters: publication date 2020-2026, research articles and full proceedings papers, English. Retrieved: 121.

**Wiley Online Library.** ("artificial intelligence" OR "machine learning" OR "deep learning" OR "convolutional neural network*" OR CNN OR "3D CNN" OR attention OR transformer* OR "vision transformer*" OR "graph neural network*" OR radiomic* OR autoencoder* OR "generative adversarial network*" OR "self-supervised learning" OR "transfer learning" OR "multimodal learning") AND ("brain imaging" OR neuroimaging OR "magnetic resonance imaging" OR MRI OR fMRI OR "functional MRI" OR DTI OR "diffusion tensor imaging" OR "computed tomography" OR CT OR CTA OR CTP OR PET OR SPECT OR EEG OR MEG OR connectom*) AND ("neurological disease*" OR stroke OR "cerebrovascular disease*" OR Alzheimer* OR dementia OR Parkinson* OR "brain tumor*" OR "brain tumour*" OR glioma* OR epilepsy OR seizure* OR "multiple sclerosis" OR "traumatic brain injury" OR autism OR "disorder* of consciousness") AND (diagnos* OR detect* OR classif* OR segment* OR localiz* OR stag* OR grad* OR predict* OR "computer-aided diagnosis" OR "computer assisted diagnosis"), searched in title, abstract, and keywords where supported. Filters: journals, 1 January 2020 to 30 June 2026, English. Wiley was used as a supplemental publisher-platform sensitivity search rather than as a substitute for an index. Retrieved: 96.

**PubMed/MEDLINE.** (("Artificial Intelligence"[Mesh] OR "Machine Learning"[Mesh] OR "Deep Learning"[tiab] OR "artificial intelligence"[tiab] OR "machine learning"[tiab] OR CNN[tiab] OR transformer*[tiab] OR radiomic*[tiab]) AND ("Neuroimaging"[Mesh] OR "Magnetic Resonance Imaging"[Mesh] OR "Tomography, X-Ray Computed"[Mesh] OR "Positron-Emission Tomography"[Mesh] OR "Electroencephalography"[Mesh] OR neuroimaging[tiab] OR MRI[tiab] OR fMRI[tiab] OR DTI[tiab] OR CT[tiab] OR PET[tiab] OR SPECT[tiab] OR EEG[tiab] OR MEG[tiab]) AND ("Nervous System Diseases"[Mesh] OR stroke[tiab] OR Alzheimer*[tiab] OR dementia[tiab] OR Parkinson*[tiab] OR glioma*[tiab] OR epilepsy[tiab] OR "multiple sclerosis"[tiab] OR autism[tiab]) AND (diagnos*[tiab] OR detect*[tiab] OR classif*[tiab] OR segment*[tiab] OR localiz*[tiab] OR predict*[tiab])) AND ("2020/01/01"[Date - Publication] : "2026/06/30"[Date - Publication]) AND humans[MeSH Terms] AND english[Language]. Retrieved: 72.

**CINAHL Complete.** ((MH "Artificial Intelligence+") OR (MH "Machine Learning+") OR TX ("artificial intelligence" OR "machine learning" OR "deep learning" OR "convolutional neural network*" OR CNN OR "3D CNN" OR attention OR transformer* OR "vision transformer*" OR "graph neural network*" OR radiomic* OR autoencoder* OR "generative adversarial network*" OR "self-supervised learning" OR "transfer learning" OR "multimodal learning")) AND ((MH "Neuroimaging+") OR (MH "Magnetic Resonance Imaging+") OR (MH "Tomography, X-Ray Computed+") OR TX ("brain imaging" OR neuroimaging OR "magnetic resonance imaging" OR MRI OR fMRI OR "functional MRI" OR DTI OR "diffusion tensor imaging" OR "computed tomography" OR CT OR CTA OR CTP OR PET OR SPECT OR EEG OR MEG OR connectom*)) AND ((MH "Nervous System Diseases+") OR TX ("neurological disease*" OR stroke OR "cerebrovascular disease*" OR Alzheimer* OR dementia OR Parkinson* OR "brain tumor*" OR "brain tumour*" OR glioma* OR epilepsy OR seizure* OR "multiple sclerosis" OR "traumatic brain injury" OR autism OR "disorder* of consciousness")) AND TX (diagnos* OR detect* OR classif* OR segment* OR localiz* OR stag* OR grad* OR predict* OR "computer-aided diagnosis" OR "computer assisted diagnosis"). Limiters: Published Date 20200101-20260630, English Language, Human, Research Article. Retrieved: 34.

**Cochrane Library.** (("artificial intelligence" OR "machine learning" OR "deep learning" OR "convolutional neural network*" OR CNN OR "3D CNN" OR attention OR transformer* OR "vision transformer*" OR "graph neural network*" OR radiomic* OR autoencoder* OR "generative adversarial network*" OR "self-supervised learning" OR "transfer learning" OR "multimodal learning")):ti,ab,kw AND (("brain imaging" OR neuroimaging OR "magnetic resonance imaging" OR MRI OR fMRI OR "functional MRI" OR DTI OR "diffusion tensor imaging" OR "computed tomography" OR CT OR CTA OR CTP OR PET OR SPECT OR EEG OR MEG OR connectom*)):ti,ab,kw AND (("neurological disease*" OR stroke OR "cerebrovascular disease*" OR Alzheimer* OR dementia OR Parkinson* OR "brain tumor*" OR "brain tumour*" OR glioma* OR epilepsy OR seizure* OR "multiple sclerosis" OR "traumatic brain injury" OR autism OR "disorder* of consciousness")):ti,ab,kw AND ((diagnos* OR detect* OR classif* OR segment* OR localiz* OR stag* OR grad* OR predict* OR "computer-aided diagnosis" OR "computer assisted diagnosis")):ti,ab,kw, with publication year 2020-2026 and Trials/Other Reviews screened for eligible original reports. Retrieved: 14.

## 1.2 Appendix B. Eligibility re-audit and study-level evidence matrix

Table S0 audits every record in the previous 137-row matrix against the prespecified eligibility criteria. Fourteen records were reclassified as contextual or otherwise ineligible, leaving 123 eligible studies. Tables S1a and S1b contain only those 123 studies. Full bibliographic details remain in the References section because contextual sources are still cited for methods or interpretation.

**Table S0. Record-level eligibility re-audit of the 137 entries in the previous evidence matrix**

| **Audit ID** | **Prior ID** | **Citation** | **Study title** | **Decision** | **Final ID** | **Reason** |
| --- | --- | --- | --- | --- | --- | --- |
| A001 | S001 | Abrol et al., 2020 | Deep residual learning for neuroimaging: An application to predict progression to Alzheimer's disease | Included | S001 | Eligible peer-reviewed original human neuroimaging or EEG/MEG diagnostic, prognostic, segmentation, benchmark, or validation study. |
| A002 | S002 | Abrol et al., 2021 | Deep learning encodes robust discriminative neuroimaging representations to outperform standard machine learning | Included | S002 | Eligible peer-reviewed original human neuroimaging or EEG/MEG diagnostic, prognostic, segmentation, benchmark, or validation study. |
| A003 | S003 | Akbari et al., 2020 | Histopathology-validated machine learning radiographic biomarker for noninvasive discrimination between true progression and pseudo-progression in glioblastoma | Included | S003 | Eligible peer-reviewed original human neuroimaging or EEG/MEG diagnostic, prognostic, segmentation, benchmark, or validation study. |
| A004 | S004 | Almuqhim et al., 2021 | ASD-SAENet: A Sparse Autoencoder, and Deep-Neural Network Model for Detecting Autism Spectrum Disorder (ASD) Using fMRI Data | Included | S004 | Eligible peer-reviewed original human neuroimaging or EEG/MEG diagnostic, prognostic, segmentation, benchmark, or validation study. |
| A005 | S005 | Amiri et al., 2023 | Multimodal prediction of residual consciousness in the intensive care unit: the CONNECT-ME study | Included | S005 | Eligible peer-reviewed original human neuroimaging or EEG/MEG diagnostic, prognostic, segmentation, benchmark, or validation study. |
| A006 | S006 | Arab et al., 2020 | A fast and fully-automated deep-learning approach for accurate hemorrhage segmentation and volume quantification in non-contrast whole-head CT | Included | S006 | Eligible peer-reviewed original human neuroimaging or EEG/MEG diagnostic, prognostic, segmentation, benchmark, or validation study. |
| A007 | S007 | Bashyam et al., 2020 | MRI signatures of brain age and disease over the lifespan based on a deep brain network and 14 468 individuals worldwide | Included | S007 | Eligible peer-reviewed original human neuroimaging or EEG/MEG diagnostic, prognostic, segmentation, benchmark, or validation study. |
| A008 | S008 | Baur et al., 2021 | Autoencoders for unsupervised anomaly segmentation in brain MR images: A comparative study | Included | S008 | Eligible peer-reviewed original human neuroimaging or EEG/MEG diagnostic, prognostic, segmentation, benchmark, or validation study. |
| A009 | S009 | Beheshti et al., 2020 | T1-weighted MRI-driven Brain Age Estimation in Alzheimer's Disease and Parkinson's Disease | Included | S009 | Eligible peer-reviewed original human neuroimaging or EEG/MEG diagnostic, prognostic, segmentation, benchmark, or validation study. |
| A010 | S010 | Bessadok et al., 2023 | Graph Neural Networks in Network Neuroscience | Contextual | Contextual | Review of graph-neural-network methods; no original eligible human study was reported. |
| A011 | S011 | Bhattacharya et al., 2022 | Epileptic Seizure Prediction Using Deep Transformer Model | Included | S010 | Eligible peer-reviewed original human neuroimaging or EEG/MEG diagnostic, prognostic, segmentation, benchmark, or validation study. |
| A012 | S012 | Billot et al., 2023 | Robust machine learning segmentation for large-scale analysis of heterogeneous clinical brain MRI datasets | Included | S011 | Eligible peer-reviewed original human neuroimaging or EEG/MEG diagnostic, prognostic, segmentation, benchmark, or validation study. |
| A013 | S013 | Bouman et al., 2023 | Multicenter Evaluation of AI-generated DIR and PSIR for Cortical and Juxtacortical Multiple Sclerosis Lesion Detection | Included | S012 | Eligible peer-reviewed original human neuroimaging or EEG/MEG diagnostic, prognostic, segmentation, benchmark, or validation study. |
| A014 | S014 | Boutet et al., 2021 | Predicting optimal deep brain stimulation parameters for Parkinson's disease using functional MRI and machine learning | Included | S013 | Eligible peer-reviewed original human neuroimaging or EEG/MEG diagnostic, prognostic, segmentation, benchmark, or validation study. |
| A015 | S015 | Brugnara et al., 2020 | Multimodal Predictive Modeling of Endovascular Treatment Outcome for Acute Ischemic Stroke Using Machine-Learning | Included | S014 | Eligible peer-reviewed original human neuroimaging or EEG/MEG diagnostic, prognostic, segmentation, benchmark, or validation study. |
| A016 | S016 | Calabrese et al., 2020 | A fully automated artificial intelligence method for non-invasive, imaging-based identification of genetic alterations in glioblastomas | Included | S015 | Eligible peer-reviewed original human neuroimaging or EEG/MEG diagnostic, prognostic, segmentation, benchmark, or validation study. |
| A017 | S017 | Camacho et al., 2023 | Explainable classification of Parkinson's disease using deep learning trained on a large multi-center database of T1-weighted MRI datasets | Included | S016 | Eligible peer-reviewed original human neuroimaging or EEG/MEG diagnostic, prognostic, segmentation, benchmark, or validation study. |
| A018 | S018 | Cao et al., 2020 | A Radiomics Approach to Predicting Parkinson's Disease by Incorporating Whole-Brain Functional Activity and Gray Matter Structure | Included | S017 | Eligible peer-reviewed original human neuroimaging or EEG/MEG diagnostic, prognostic, segmentation, benchmark, or validation study. |
| A019 | S019 | Carré et al., 2020 | Standardization of brain MR images across machines and protocols: bridging the gap for MRI-based radiomics | Included | S018 | Eligible peer-reviewed original human neuroimaging or EEG/MEG diagnostic, prognostic, segmentation, benchmark, or validation study. |
| A020 | S020 | Castellazzi et al., 2020 | A Machine Learning Approach for the Differential Diagnosis of Alzheimer and Vascular Dementia Fed by MRI Selected Features | Included | S019 | Eligible peer-reviewed original human neuroimaging or EEG/MEG diagnostic, prognostic, segmentation, benchmark, or validation study. |
| A021 | S021 | Cheng et al., 2022 | A Fully Automated Multimodal MRI-Based Multi-Task Learning for Glioma Segmentation and IDH Genotyping | Included | S020 | Eligible peer-reviewed original human neuroimaging or EEG/MEG diagnostic, prognostic, segmentation, benchmark, or validation study. |
| A022 | S022 | Choi et al., 2021 | Fully automated hybrid approach to predict the IDH mutation status of gliomas via deep learning and radiomics | Included | S021 | Eligible peer-reviewed original human neuroimaging or EEG/MEG diagnostic, prognostic, segmentation, benchmark, or validation study. |
| A023 | S023 | Chougar et al., 2021 | Automated Categorization of Parkinsonian Syndromes Using Magnetic Resonance Imaging in a Clinical Setting | Included | S022 | Eligible peer-reviewed original human neuroimaging or EEG/MEG diagnostic, prognostic, segmentation, benchmark, or validation study. |
| A024 | S024 | Clèrigues et al., 2020 | Acute and sub-acute stroke lesion segmentation from multimodal MRI | Included | S023 | Eligible peer-reviewed original human neuroimaging or EEG/MEG diagnostic, prognostic, segmentation, benchmark, or validation study. |
| A025 | S025 | Conte et al., 2021 | Generative Adversarial Networks to Synthesize Missing T1 and FLAIR MRI Sequences for Use in a Multisequence Brain Tumor Segmentation Model | Included | S024 | Eligible peer-reviewed original human neuroimaging or EEG/MEG diagnostic, prognostic, segmentation, benchmark, or validation study. |
| A026 | S026 | da Silva Lourenço et al., 2021 | Machine learning for detection of interictal epileptiform discharges | Contextual | Contextual | Review article; no original eligible diagnostic or validation cohort was reported. |
| A027 | S027 | Dinsdale et al., 2021 | Deep learning-based unlearning of dataset bias for MRI harmonisation and confound removal | Included | S025 | Eligible peer-reviewed original human neuroimaging or EEG/MEG diagnostic, prognostic, segmentation, benchmark, or validation study. |
| A028 | S028 | Duc et al., 2020 | 3D-Deep Learning Based Automatic Diagnosis of Alzheimer's Disease with Joint MMSE Prediction Using Resting-State fMRI | Included | S026 | Eligible peer-reviewed original human neuroimaging or EEG/MEG diagnostic, prognostic, segmentation, benchmark, or validation study. |
| A029 | S029 | El-Sappagh et al., 2021 | A multilayer multimodal detection and prediction model based on explainable artificial intelligence for Alzheimer's disease | Included | S027 | Eligible peer-reviewed original human neuroimaging or EEG/MEG diagnostic, prognostic, segmentation, benchmark, or validation study. |
| A030 | S030 | Elijovich et al., 2022 | Automated emergent large vessel occlusion detection by artificial intelligence improves stroke workflow in a hub and spoke stroke system of care | Included | S028 | Eligible peer-reviewed original human neuroimaging or EEG/MEG diagnostic, prognostic, segmentation, benchmark, or validation study. |
| A031 | S031 | Eshaghi et al., 2021 | Identifying multiple sclerosis subtypes using unsupervised machine learning and MRI data | Included | S029 | Eligible peer-reviewed original human neuroimaging or EEG/MEG diagnostic, prognostic, segmentation, benchmark, or validation study. |
| A032 | S032 | Finck et al., 2020 | Deep-Learning Generated Synthetic Double Inversion Recovery Images Improve Multiple Sclerosis Lesion Detection | Included | S030 | Eligible peer-reviewed original human neuroimaging or EEG/MEG diagnostic, prognostic, segmentation, benchmark, or validation study. |
| A033 | S033 | Flanders et al., 2020 | Construction of a Machine Learning Dataset through Collaboration: The RSNA 2019 Brain CT Hemorrhage Challenge | Included | S031 | Eligible peer-reviewed original human neuroimaging or EEG/MEG diagnostic, prognostic, segmentation, benchmark, or validation study. |
| A034 | S034 | Fürbass et al., 2020 | An artificial intelligence-based EEG algorithm for detection of epileptiform EEG discharges: Validation against the diagnostic gold standard | Included | S032 | Eligible peer-reviewed original human neuroimaging or EEG/MEG diagnostic, prognostic, segmentation, benchmark, or validation study. |
| A035 | S035 | Gabeff et al., 2021 | Interpreting deep learning models for epileptic seizure detection on EEG signals | Included | S033 | Eligible peer-reviewed original human neuroimaging or EEG/MEG diagnostic, prognostic, segmentation, benchmark, or validation study. |
| A036 | S036 | Gill et al., 2021 | Multicenter Validation of a Deep Learning Detection Algorithm for Focal Cortical Dysplasia | Included | S034 | Eligible peer-reviewed original human neuroimaging or EEG/MEG diagnostic, prognostic, segmentation, benchmark, or validation study. |
| A037 | S037 | Gleichgerrcht et al., 2021 | Artificial intelligence for classification of temporal lobe epilepsy with ROI-level MRI data: A worldwide ENIGMA-Epilepsy study | Included | S035 | Eligible peer-reviewed original human neuroimaging or EEG/MEG diagnostic, prognostic, segmentation, benchmark, or validation study. |
| A038 | S038 | Goceri et al., 2023 | Medical image data augmentation: techniques, comparisons and interpretations | Contextual | Contextual | Review of augmentation methods; no original neurological imaging study was reported. |
| A039 | S039 | Gros et al., 2021 | SoftSeg: Advantages of soft versus binary training for image segmentation | Included | S036 | Eligible peer-reviewed original human neuroimaging or EEG/MEG diagnostic, prognostic, segmentation, benchmark, or validation study. |
| A040 | S040 | Hakim et al., 2021 | Predicting Infarct Core From Computed Tomography Perfusion in Acute Ischemia With Machine Learning: Lessons From the ISLES Challenge | Included | S037 | Eligible peer-reviewed original human neuroimaging or EEG/MEG diagnostic, prognostic, segmentation, benchmark, or validation study. |
| A041 | S041 | Henschel et al., 2020 | FastSurfer - A fast and accurate deep learning based neuroimaging pipeline | Included | S038 | Eligible peer-reviewed original human neuroimaging or EEG/MEG diagnostic, prognostic, segmentation, benchmark, or validation study. |
| A042 | S042 | Hollon et al., 2020 | Near real-time intraoperative brain tumor diagnosis using stimulated Raman histology and deep neural networks | Included | S039 | Eligible peer-reviewed original human neuroimaging or EEG/MEG diagnostic, prognostic, segmentation, benchmark, or validation study. |
| A043 | S043 | Hollon et al., 2023 | Artificial-intelligence-based molecular classification of diffuse gliomas using rapid, label-free optical imaging | Included | S040 | Eligible peer-reviewed original human neuroimaging or EEG/MEG diagnostic, prognostic, segmentation, benchmark, or validation study. |
| A044 | S044 | Hosseinzadeh et al., 2023 | Prediction of Cognitive Decline in Parkinson's Disease Using Clinical and DAT SPECT Imaging Features, and Hybrid Machine Learning Systems | Included | S041 | Eligible peer-reviewed original human neuroimaging or EEG/MEG diagnostic, prognostic, segmentation, benchmark, or validation study. |
| A045 | S045 | Ingalhalikar et al., 2021 | Functional Connectivity-Based Prediction of Autism on Site Harmonized ABIDE Dataset | Included | S042 | Eligible peer-reviewed original human neuroimaging or EEG/MEG diagnostic, prognostic, segmentation, benchmark, or validation study. |
| A046 | S046 | Jayachandran Preetha et al., 2021 | Deep-learning-based synthesis of post-contrast T1-weighted MRI for tumour response assessment in neuro-oncology: a multicentre, retrospective cohort study | Included | S043 | Eligible peer-reviewed original human neuroimaging or EEG/MEG diagnostic, prognostic, segmentation, benchmark, or validation study. |
| A047 | S047 | Jiang et al., 2022 | SwinBTS: A Method for 3D Multimodal Brain Tumor Segmentation Using Swin Transformer | Included | S044 | Eligible peer-reviewed original human neuroimaging or EEG/MEG diagnostic, prognostic, segmentation, benchmark, or validation study. |
| A048 | S048 | Kamraoui et al., 2022 | DeepLesionBrain: Towards a broader deep-learning generalization for multiple sclerosis lesion segmentation | Included | S045 | Eligible peer-reviewed original human neuroimaging or EEG/MEG diagnostic, prognostic, segmentation, benchmark, or validation study. |
| A049 | S049 | Khodatars et al., 2021 | Deep learning for neuroimaging-based diagnosis and rehabilitation of Autism Spectrum Disorder: A review | Contextual | Contextual | Review article; no original eligible human study was reported. |
| A050 | S050 | Kim et al., 2020 | Deep Learning-Based Method to Differentiate Neuromyelitis Optica Spectrum Disorder From Multiple Sclerosis | Included | S046 | Eligible peer-reviewed original human neuroimaging or EEG/MEG diagnostic, prognostic, segmentation, benchmark, or validation study. |
| A051 | S051 | Kohoutová et al., 2020 | Toward a unified framework for interpreting machine-learning models in neuroimaging | Contextual | Contextual | Protocol and interpretability framework rather than an original eligible diagnostic study. |
| A052 | S052 | Kumar et al., 2020 | CSNet: A new DeepNet framework for ischemic stroke lesion segmentation | Included | S047 | Eligible peer-reviewed original human neuroimaging or EEG/MEG diagnostic, prognostic, segmentation, benchmark, or validation study. |
| A053 | S053 | Kural et al., 2022 | Accurate identification of EEG recordings with interictal epileptiform discharges using a hybrid approach: Artificial intelligence supervised by human experts | Included | S048 | Eligible peer-reviewed original human neuroimaging or EEG/MEG diagnostic, prognostic, segmentation, benchmark, or validation study. |
| A054 | S054 | La Rosa et al., 2020 | Multiple sclerosis cortical and WM lesion segmentation at 3T MRI: a deep learning method based on FLAIR and MP2RAGE | Included | S049 | Eligible peer-reviewed original human neuroimaging or EEG/MEG diagnostic, prognostic, segmentation, benchmark, or validation study. |
| A055 | S055 | Lanka et al., 2020 | Supervised machine learning for diagnostic classification from large-scale neuroimaging datasets | Included | S050 | Eligible peer-reviewed original human neuroimaging or EEG/MEG diagnostic, prognostic, segmentation, benchmark, or validation study. |
| A056 | S056 | Larivière et al., 2020 | Functional connectome contractions in temporal lobe epilepsy: Microstructural underpinnings and predictors of surgical outcome | Included | S051 | Eligible peer-reviewed original human neuroimaging or EEG/MEG diagnostic, prognostic, segmentation, benchmark, or validation study. |
| A057 | S057 | Lee et al., 2022 | Decomposing MRI phenotypic heterogeneity in epilepsy: a step towards personalized classification | Included | S052 | Eligible peer-reviewed original human neuroimaging or EEG/MEG diagnostic, prognostic, segmentation, benchmark, or validation study. |
| A058 | S058 | Lee et al., 2023 | Prediction of post-stroke cognitive impairment after acute ischemic stroke using machine learning | Included | S053 | Eligible peer-reviewed original human neuroimaging or EEG/MEG diagnostic, prognostic, segmentation, benchmark, or validation study. |
| A059 | S059 | Li et al., 2021 | Deep Learning for Hemorrhagic Lesion Detection and Segmentation on Brain CT Images | Included | S054 | Eligible peer-reviewed original human neuroimaging or EEG/MEG diagnostic, prognostic, segmentation, benchmark, or validation study. |
| A060 | S060 | Liu et al., 2021 | Deep learning-based detection and segmentation of diffusion abnormalities in acute ischemic stroke | Included | S055 | Eligible peer-reviewed original human neuroimaging or EEG/MEG diagnostic, prognostic, segmentation, benchmark, or validation study. |
| A061 | S061 | Liu et al., 2022 | Generalizable deep learning model for early Alzheimer's disease detection from structural MRIs | Included | S056 | Eligible peer-reviewed original human neuroimaging or EEG/MEG diagnostic, prognostic, segmentation, benchmark, or validation study. |
| A062 | S062 | Magesh et al., 2020 | An Explainable Machine Learning Model for Early Detection of Parkinson's Disease using LIME on DaTSCAN Imagery | Included | S057 | Eligible peer-reviewed original human neuroimaging or EEG/MEG diagnostic, prognostic, segmentation, benchmark, or validation study. |
| A063 | S063 | Maggi et al., 2020 | CVSnet: A machine learning approach for automated central vein sign assessment in multiple sclerosis | Included | S058 | Eligible peer-reviewed original human neuroimaging or EEG/MEG diagnostic, prognostic, segmentation, benchmark, or validation study. |
| A064 | S064 | Martinez-Gutierrez et al., 2023 | Automated Large Vessel Occlusion Detection Software and Thrombectomy Treatment Times: A Cluster Randomized Clinical Trial | Included | S059 | Eligible peer-reviewed original human neuroimaging or EEG/MEG diagnostic, prognostic, segmentation, benchmark, or validation study. |
| A065 | S065 | Matsoukas et al., 2023 | AI software detection of large vessel occlusion stroke on CT angiography: a real-world prospective diagnostic test accuracy study | Included | S060 | Eligible peer-reviewed original human neuroimaging or EEG/MEG diagnostic, prognostic, segmentation, benchmark, or validation study. |
| A066 | S066 | Mei et al., 2022 | RadImageNet: An Open Radiologic Deep Learning Research Dataset for Effective Transfer Learning | Contextual | Contextual | General radiology dataset and transfer-learning resource without a primary brain-disease endpoint. |
| A067 | S067 | Monteiro et al., 2020 | Multiclass semantic segmentation and quantification of traumatic brain injury lesions on head CT using deep learning: an algorithm development and multicentre validation study | Included | S061 | Eligible peer-reviewed original human neuroimaging or EEG/MEG diagnostic, prognostic, segmentation, benchmark, or validation study. |
| A068 | S068 | Moons et al., 2025 | PROBAST+AI: an updated quality, risk of bias, and applicability assessment tool for prediction models using regression or artificial intelligence methods | Contextual | Contextual | Risk-of-bias and applicability assessment tool, not an original eligible human study. |
| A069 | S069 | Muckley et al., 2021 | Results of the 2020 fastMRI Challenge for Machine Learning MR Image Reconstruction | Included | S062 | Eligible peer-reviewed original human neuroimaging or EEG/MEG diagnostic, prognostic, segmentation, benchmark, or validation study. |
| A070 | S070 | Mårtensson et al., 2020 | The reliability of a deep learning model in clinical out-of-distribution MRI data: A multicohort study | Included | S063 | Eligible peer-reviewed original human neuroimaging or EEG/MEG diagnostic, prognostic, segmentation, benchmark, or validation study. |
| A071 | S071 | Naser et al., 2020 | Brain tumor segmentation and grading of lower-grade glioma using deep learning in MRI images | Included | S064 | Eligible peer-reviewed original human neuroimaging or EEG/MEG diagnostic, prognostic, segmentation, benchmark, or validation study. |
| A072 | S072 | Nishi et al., 2020 | Deep Learning-Derived High-Level Neuroimaging Features Predict Clinical Outcomes for Large Vessel Occlusion | Included | S065 | Eligible peer-reviewed original human neuroimaging or EEG/MEG diagnostic, prognostic, segmentation, benchmark, or validation study. |
| A073 | S073 | Pan et al., 2020 | Early Detection of Alzheimer's Disease Using Magnetic Resonance Imaging: A Novel Approach Combining Convolutional Neural Networks and Ensemble Learning | Included | S066 | Eligible peer-reviewed original human neuroimaging or EEG/MEG diagnostic, prognostic, segmentation, benchmark, or validation study. |
| A074 | S074 | Park et al., 2021 | Differences in subcortico-cortical interactions identified from connectome and microcircuit models in autism | Included | S067 | Eligible peer-reviewed original human neuroimaging or EEG/MEG diagnostic, prognostic, segmentation, benchmark, or validation study. |
| A075 | S075 | Pavel et al., 2020 | A machine-learning algorithm for neonatal seizure recognition: a multicentre, randomised, controlled trial | Included | S068 | Eligible peer-reviewed original human neuroimaging or EEG/MEG diagnostic, prognostic, segmentation, benchmark, or validation study. |
| A076 | S076 | Pease et al., 2022 | Outcome Prediction in Patients with Severe Traumatic Brain Injury Using Deep Learning from Head CT Scans | Included | S069 | Eligible peer-reviewed original human neuroimaging or EEG/MEG diagnostic, prognostic, segmentation, benchmark, or validation study. |
| A077 | S077 | Pei et al., 2020 | Context aware deep learning for brain tumor segmentation, subtype classification, and survival prediction using radiology images | Included | S070 | Eligible peer-reviewed original human neuroimaging or EEG/MEG diagnostic, prognostic, segmentation, benchmark, or validation study. |
| A078 | S078 | Peng et al., 2021 | Accurate brain age prediction with lightweight deep neural networks | Included | S071 | Eligible peer-reviewed original human neuroimaging or EEG/MEG diagnostic, prognostic, segmentation, benchmark, or validation study. |
| A079 | S079 | Pinaya et al., 2021 | Using normative modelling to detect disease progression in mild cognitive impairment and Alzheimer's disease in a cross-sectional multi-cohort study | Included | S072 | Eligible peer-reviewed original human neuroimaging or EEG/MEG diagnostic, prognostic, segmentation, benchmark, or validation study. |
| A080 | S080 | Popuri et al., 2020 | Using machine learning to quantify structural MRI neurodegeneration patterns of Alzheimer's disease into dementia score: Independent validation on 8,834 images from ADNI, AIBL, OASIS, and MIRIAD databases | Included | S073 | Eligible peer-reviewed original human neuroimaging or EEG/MEG diagnostic, prognostic, segmentation, benchmark, or validation study. |
| A081 | S081 | Qiu et al., 2020a | Development and validation of an interpretable deep learning framework for Alzheimer's disease classification | Included | S074 | Eligible peer-reviewed original human neuroimaging or EEG/MEG diagnostic, prognostic, segmentation, benchmark, or validation study. |
| A082 | S082 | Qiu et al., 2020b | Machine Learning for Detecting Early Infarction in Acute Stroke with Non-Contrast-enhanced CT | Included | S075 | Eligible peer-reviewed original human neuroimaging or EEG/MEG diagnostic, prognostic, segmentation, benchmark, or validation study. |
| A083 | S083 | Qiu et al., 2022 | Multimodal deep learning for Alzheimer's disease dementia assessment | Included | S076 | Eligible peer-reviewed original human neuroimaging or EEG/MEG diagnostic, prognostic, segmentation, benchmark, or validation study. |
| A084 | S084 | Radua et al., 2020 | Increased power by harmonizing structural MRI site differences with the ComBat batch adjustment method in ENIGMA | Included | S077 | Eligible peer-reviewed original human neuroimaging or EEG/MEG diagnostic, prognostic, segmentation, benchmark, or validation study. |
| A085 | S085 | Rakić et al., 2021 | icobrain ms 5.1: Combining unsupervised and supervised approaches for improving the detection of multiple sclerosis lesions | Included | S078 | Eligible peer-reviewed original human neuroimaging or EEG/MEG diagnostic, prognostic, segmentation, benchmark, or validation study. |
| A086 | S086 | Ranjbarzadeh et al., 2021 | Brain tumor segmentation based on deep learning and an attention mechanism using MRI multi-modalities brain images | Included | S079 | Eligible peer-reviewed original human neuroimaging or EEG/MEG diagnostic, prognostic, segmentation, benchmark, or validation study. |
| A087 | S087 | Roca et al., 2020 | Artificial intelligence to predict clinical disability in patients with multiple sclerosis using FLAIR MRI | Included | S080 | Eligible peer-reviewed original human neuroimaging or EEG/MEG diagnostic, prognostic, segmentation, benchmark, or validation study. |
| A088 | S088 | Seyam et al., 2022 | Utilization of Artificial Intelligence-based Intracranial Hemorrhage Detection on Emergent Noncontrast CT Images in Clinical Workflow | Included | S081 | Eligible peer-reviewed original human neuroimaging or EEG/MEG diagnostic, prognostic, segmentation, benchmark, or validation study. |
| A089 | S089 | Shi et al., 2020 | A clinically applicable deep-learning model for detecting intracranial aneurysm in computed tomography angiography images | Included | S082 | Eligible peer-reviewed original human neuroimaging or EEG/MEG diagnostic, prognostic, segmentation, benchmark, or validation study. |
| A090 | S090 | Shin et al., 2021 | Cortical Thickness from MRI to Predict Conversion from Mild Cognitive Impairment to Dementia in Parkinson Disease: A Machine Learning-based Model | Included | S083 | Eligible peer-reviewed original human neuroimaging or EEG/MEG diagnostic, prognostic, segmentation, benchmark, or validation study. |
| A091 | S091 | Shu et al., 2021 | Predicting the progression of Parkinson's disease using conventional MRI and machine learning: An application of radiomic biomarkers in whole-brain white matter | Included | S084 | Eligible peer-reviewed original human neuroimaging or EEG/MEG diagnostic, prognostic, segmentation, benchmark, or validation study. |
| A092 | S092 | Sisodiya et al., 2020 | The ENIGMA-Epilepsy working group: Mapping disease from large data sets | Contextual | Contextual | Review of consortium projects rather than a single original eligible study. |
| A093 | S093 | Solana-Lavalle et al., 2021 | Classification of PPMI MRI scans with voxel-based morphometry and machine learning to assist in the diagnosis of Parkinson's disease | Included | S085 | Eligible peer-reviewed original human neuroimaging or EEG/MEG diagnostic, prognostic, segmentation, benchmark, or validation study. |
| A094 | S094 | Song et al., 2021 | An Effective Multimodal Image Fusion Method Using MRI and PET for Alzheimer's Disease Diagnosis | Included | S086 | Eligible peer-reviewed original human neuroimaging or EEG/MEG diagnostic, prognostic, segmentation, benchmark, or validation study. |
| A095 | S095 | Soun et al., 2021 | Artificial Intelligence and Acute Stroke Imaging | Contextual | Contextual | Review article; no original eligible diagnostic or validation cohort was reported. |
| A096 | S096 | Spitzer et al., 2022 | Interpretable surface-based detection of focal cortical dysplasias: a Multi-centre Epilepsy Lesion Detection study | Included | S087 | Eligible peer-reviewed original human neuroimaging or EEG/MEG diagnostic, prognostic, segmentation, benchmark, or validation study. |
| A097 | S097 | Spooner et al., 2020 | A comparison of machine learning methods for survival analysis of high-dimensional clinical data for dementia prediction | Included | S088 | Eligible peer-reviewed original human neuroimaging or EEG/MEG diagnostic, prognostic, segmentation, benchmark, or validation study. |
| A098 | S098 | Steyaert et al., 2023 | Multimodal deep learning to predict prognosis in adult and pediatric brain tumors | Included | S089 | Eligible peer-reviewed original human neuroimaging or EEG/MEG diagnostic, prognostic, segmentation, benchmark, or validation study. |
| A099 | S099 | Stib et al., 2020 | Detecting Large Vessel Occlusion at Multiphase CT Angiography by Using a Deep Convolutional Neural Network | Included | S090 | Eligible peer-reviewed original human neuroimaging or EEG/MEG diagnostic, prognostic, segmentation, benchmark, or validation study. |
| A100 | S100 | Storelli et al., 2022 | A Deep Learning Approach to Predicting Disease Progression in Multiple Sclerosis Using Magnetic Resonance Imaging | Included | S091 | Eligible peer-reviewed original human neuroimaging or EEG/MEG diagnostic, prognostic, segmentation, benchmark, or validation study. |
| A101 | S101 | Storelli et al., 2026 | Explainable Artificial Intelligence to Predict Neurocognitive Disorder Progression in Multiple Sclerosis Using MRI and Clinical Data | Included | S092 | Eligible peer-reviewed original human neuroimaging or EEG/MEG diagnostic, prognostic, segmentation, benchmark, or validation study. |
| A102 | S102 | Sun et al., 2024 | Identification of Parkinson's disease and multiple system atrophy using multimodal PET/MRI radiomics | Included | S093 | Eligible peer-reviewed original human neuroimaging or EEG/MEG diagnostic, prognostic, segmentation, benchmark, or validation study. |
| A103 | S103 | Sunwoo et al., 2026 | AI assisted detection of large vessel occlusion on non-contrast CT: multinational validation and reader study | Included | S094 | Eligible peer-reviewed original human neuroimaging or EEG/MEG diagnostic, prognostic, segmentation, benchmark, or validation study. |
| A104 | S104 | Talai et al., 2021 | Utility of Multi-Modal MRI for Differentiating of Parkinson's Disease and Progressive Supranuclear Palsy Using Machine Learning | Included | S095 | Eligible peer-reviewed original human neuroimaging or EEG/MEG diagnostic, prognostic, segmentation, benchmark, or validation study. |
| A105 | S105 | Tang et al., 2026 | Decoupling Alzheimer Disease Pathologic Abnormalities at PET with Improved Clinical Relevance by Interpretable Adversarial Decomposition Learning | Included | S096 | Eligible peer-reviewed original human neuroimaging or EEG/MEG diagnostic, prognostic, segmentation, benchmark, or validation study. |
| A106 | S106 | Tanno et al., 2021 | Uncertainty modelling in deep learning for safer neuroimage enhancement: Demonstration in diffusion MRI | Included | S097 | Eligible peer-reviewed original human neuroimaging or EEG/MEG diagnostic, prognostic, segmentation, benchmark, or validation study. |
| A107 | S107 | Thakur et al., 2020 | Brain extraction on MRI scans in presence of diffuse glioma: Multi-institutional performance evaluation of deep learning methods and robust modality-agnostic training | Included | S098 | Eligible peer-reviewed original human neuroimaging or EEG/MEG diagnostic, prognostic, segmentation, benchmark, or validation study. |
| A108 | S108 | Theocharidis et al., 2026 | Applying ensemble machine learning techniques to MRI scans to predict Alzheimer's disease | Included | S099 | Eligible peer-reviewed original human neuroimaging or EEG/MEG diagnostic, prognostic, segmentation, benchmark, or validation study. |
| A109 | S109 | Thomas et al., 2020 | Classifying Autism Spectrum Disorder Using the Temporal Statistics of Resting-State Functional MRI Data With 3D Convolutional Neural Networks | Included | S100 | Eligible peer-reviewed original human neuroimaging or EEG/MEG diagnostic, prognostic, segmentation, benchmark, or validation study. |
| A110 | S110 | Thomas et al., 2026 | Accuracy of Automated Deep Learning versus Expert Clinicians for Diagnosis of Acute Lacunar Stroke on CT Perfusion | Included | S101 | Eligible peer-reviewed original human neuroimaging or EEG/MEG diagnostic, prognostic, segmentation, benchmark, or validation study. |
| A111 | S111 | Thompson et al., 2020 | ENIGMA and global neuroscience: A decade of large-scale studies of the brain in health and disease across more than 40 countries | Contextual | Contextual | Consortium review article rather than an original eligible study. |
| A112 | S112 | Tustison et al., 2021 | The ANTsX ecosystem for quantitative biological and medical imaging | Contextual | Contextual | Software ecosystem and methodological framework without a discrete eligible disease-linked endpoint. |
| A113 | S113 | Tveit et al., 2023 | Automated Interpretation of Clinical Electroencephalograms Using Artificial Intelligence | Included | S102 | Eligible peer-reviewed original human neuroimaging or EEG/MEG diagnostic, prognostic, segmentation, benchmark, or validation study. |
| A114 | S114 | van der Voort et al., 2023 | Combined molecular subtyping, grading, and segmentation of glioma using multi-task deep learning | Included | S103 | Eligible peer-reviewed original human neuroimaging or EEG/MEG diagnostic, prognostic, segmentation, benchmark, or validation study. |
| A115 | S115 | Vasey et al., 2022 | Reporting guideline for the early-stage clinical evaluation of decision support systems driven by artificial intelligence: DECIDE-AI | Contextual | Contextual | Reporting guideline, not an original eligible human study. |
| A116 | S116 | Venugopalan et al., 2021 | Multimodal deep learning models for early detection of Alzheimer's disease stage | Included | S104 | Eligible peer-reviewed original human neuroimaging or EEG/MEG diagnostic, prognostic, segmentation, benchmark, or validation study. |
| A117 | S117 | Wen et al., 2020 | Convolutional neural networks for classification of Alzheimer's disease: Overview and reproducible evaluation | Included | S105 | Eligible peer-reviewed original human neuroimaging or EEG/MEG diagnostic, prognostic, segmentation, benchmark, or validation study. |
| A118 | S118 | Wen et al., 2022 | MVS-GCN: A prior brain structure learning-guided multi-view graph convolution network for autism spectrum disorder diagnosis | Included | S106 | Eligible peer-reviewed original human neuroimaging or EEG/MEG diagnostic, prognostic, segmentation, benchmark, or validation study. |
| A119 | S119 | Wiltgen et al., 2024 | LST-AI: A deep learning ensemble for accurate MS lesion segmentation | Included | S107 | Eligible peer-reviewed original human neuroimaging or EEG/MEG diagnostic, prognostic, segmentation, benchmark, or validation study. |
| A120 | S120 | Wu et al., 2022 | Differential Diagnosis of Parkinsonism Based on Deep Metabolic Imaging Indices | Included | S108 | Eligible peer-reviewed original human neuroimaging or EEG/MEG diagnostic, prognostic, segmentation, benchmark, or validation study. |
| A121 | S121 | Xiao et al., 2023 | Identification of different MRI atrophy progression trajectories in epilepsy by subtype and stage inference | Included | S109 | Eligible peer-reviewed original human neuroimaging or EEG/MEG diagnostic, prognostic, segmentation, benchmark, or validation study. |
| A122 | S122 | Xue et al., 2024 | AI-based differential diagnosis of dementia etiologies on multimodal data | Included | S110 | Eligible peer-reviewed original human neuroimaging or EEG/MEG diagnostic, prognostic, segmentation, benchmark, or validation study. |
| A123 | S123 | Yaman et al., 2020 | Self-supervised learning of physics-guided reconstruction neural networks without fully sampled reference data | Included | S111 | Eligible peer-reviewed original human neuroimaging or EEG/MEG diagnostic, prognostic, segmentation, benchmark, or validation study. |
| A124 | S124 | Yang et al., 2021 | A deep learning framework identifies dimensional representations of Alzheimer's Disease from brain structure | Included | S112 | Eligible peer-reviewed original human neuroimaging or EEG/MEG diagnostic, prognostic, segmentation, benchmark, or validation study. |
| A125 | S125 | Yasaka et al., 2021 | Parkinson's disease: deep learning with a parameter-weighted structural connectome matrix for diagnosis and neural circuit disorder investigation | Included | S113 | Eligible peer-reviewed original human neuroimaging or EEG/MEG diagnostic, prognostic, segmentation, benchmark, or validation study. |
| A126 | S126 | Ye et al., 2020 | Deep learning with diffusion basis spectrum imaging for classification of multiple sclerosis lesions | Included | S114 | Eligible peer-reviewed original human neuroimaging or EEG/MEG diagnostic, prognostic, segmentation, benchmark, or validation study. |
| A127 | S127 | Yin et al., 2023 | Anatomically interpretable deep learning of brain age captures domain-specific cognitive impairment | Included | S115 | Eligible peer-reviewed original human neuroimaging or EEG/MEG diagnostic, prognostic, segmentation, benchmark, or validation study. |
| A128 | S128 | Yogananda et al., 2021 | MRI-Based Deep-Learning Method for Determining Glioma MGMT Promoter Methylation Status | Included | S116 | Eligible peer-reviewed original human neuroimaging or EEG/MEG diagnostic, prognostic, segmentation, benchmark, or validation study. |
| A129 | S129 | Yu et al., 2020 | Use of Deep Learning to Predict Final Ischemic Stroke Lesions From Initial Magnetic Resonance Imaging | Included | S117 | Eligible peer-reviewed original human neuroimaging or EEG/MEG diagnostic, prognostic, segmentation, benchmark, or validation study. |
| A130 | S130 | Zhang et al., 2020a | Advances in multimodal data fusion in neuroimaging: Overview, challenges, and novel orientation | Contextual | Contextual | Review and overview article; no original eligible human study was reported. |
| A131 | S131 | Zhang et al., 2020b | DENSE-INception U-net for medical image segmentation | Contextual | Contextual | Non-neurological retinal-vessel segmentation evaluation; outside the brain-imaging population criterion. |
| A132 | S132 | Zhao et al., 2020 | Ensemble learning predicts multiple sclerosis disease course in the SUMMIT study | Included | S118 | Eligible peer-reviewed original human neuroimaging or EEG/MEG diagnostic, prognostic, segmentation, benchmark, or validation study. |
| A133 | S133 | Zhao et al., 2022 | Decoding the dopamine transporter imaging for the differential diagnosis of parkinsonism using deep learning | Included | S119 | Eligible peer-reviewed original human neuroimaging or EEG/MEG diagnostic, prognostic, segmentation, benchmark, or validation study. |
| A134 | S134 | Zhou et al., 2021 | Models Genesis | Included | S120 | Eligible peer-reviewed original human neuroimaging or EEG/MEG diagnostic, prognostic, segmentation, benchmark, or validation study. |
| A135 | S135 | Zhu et al., 2021a | An automatic machine learning approach for ischemic stroke onset time identification based on DWI and FLAIR imaging | Included | S121 | Eligible peer-reviewed original human neuroimaging or EEG/MEG diagnostic, prognostic, segmentation, benchmark, or validation study. |
| A136 | S136 | Zhu et al., 2021b | Dual Attention Multi-Instance Deep Learning for Alzheimer's Disease Diagnosis With Structural MRI | Included | S122 | Eligible peer-reviewed original human neuroimaging or EEG/MEG diagnostic, prognostic, segmentation, benchmark, or validation study. |
| A137 | S137 | Zhuge et al., 2020 | Automated glioma grading on conventional MRI images using deep convolutional neural networks | Included | S123 | Eligible peer-reviewed original human neuroimaging or EEG/MEG diagnostic, prognostic, segmentation, benchmark, or validation study. |

Supplementary Table S1 is divided into two landscape panels. Validation, performance, comparator, and external-validation fields are reproduced only when available in the retained structured extraction. Missing values are marked NR and were not reconstructed from titles, abstracts, or narrative context. Consequently, these fields support study-level inspection but not complete aggregate prevalence estimates.

**Table S1a. Eligible study identity, population, modality, dataset, and sample-size fields (N = 123)**

| **ID** | **Citation** | **Population / disease** | **Modality** | **Dataset / cohort** | **Sample size** | **Study title** |
| --- | --- | --- | --- | --- | --- | --- |
| S001 | Abrol et al., 2020 | Alzheimer disease / dementia | Structural MRI | ADNI | Not reported | Deep residual learning for neuroimaging: An application to predict progression to Alzheimer's disease |
| S002 | Abrol et al., 2021 | Cross-disease or general neuroimaging | Brain imaging modality Not reported | Not reported | Not reported | Deep learning encodes robust discriminative neuroimaging representations to outperform standard machine learning |
| S003 | Akbari et al., 2020 | Brain tumor / glioma | Brain imaging modality Not reported | Not reported | Not reported | Histopathology-validated machine learning radiographic biomarker for noninvasive discrimination between true progression and pseudo-progression in glioblastoma |
| S004 | Almuqhim et al., 2021 | Autism spectrum disorder | MRI / diffusion; fMRI; PET / SPECT | ABIDE | Not reported | ASD-SAENet: A Sparse Autoencoder, and Deep-Neural Network Model for Detecting Autism Spectrum Disorder (ASD) Using fMRI Data |
| S005 | Amiri et al., 2023 | Disorders of consciousness | Multimodal | Not reported | Not reported | Multimodal prediction of residual consciousness in the intensive care unit: the CONNECT-ME study |
| S006 | Arab et al., 2020 | Stroke / cerebrovascular disease | CT / CTA / CTP | RSNA ICH Challenge | Not reported | A fast and fully-automated deep-learning approach for accurate hemorrhage segmentation and volume quantification in non-contrast whole-head CT |
| S007 | Bashyam et al., 2020 | Dementia, tumors, PD, epilepsy; Aging and AD | MRI / diffusion | AIBL; OASIS; UK Biobank imaging | Not reported | MRI signatures of brain age and disease over the lifespan based on a deep brain network and 14 468 individuals worldwide |
| S008 | Baur et al., 2021 | Cross-disease or general neuroimaging | Brain imaging modality Not reported | Not reported | Not reported | Autoencoders for unsupervised anomaly segmentation in brain MR images: A comparative study |
| S009 | Beheshti et al., 2020 | Alzheimer disease / dementia | MRI / diffusion | ADNI; UK Biobank imaging | Not reported | T1-weighted MRI-driven Brain Age Estimation in Alzheimer's Disease and Parkinson's Disease |
| S010 | Bhattacharya et al., 2022 | Epilepsy | Brain imaging modality Not reported | Not reported | Not reported | Epileptic Seizure Prediction Using Deep Transformer Model |
| S011 | Billot et al., 2023 | Chronic stroke | MRI / diffusion | ATLAS | Not reported | Robust machine learning segmentation for large-scale analysis of heterogeneous clinical brain MRI datasets |
| S012 | Bouman et al., 2023 | Multiple sclerosis / demyelinating disease | Brain imaging modality Not reported | MSSEG / ISBI MS | Not reported | Multicenter Evaluation of AI-generated DIR and PSIR for Cortical and Juxtacortical Multiple Sclerosis Lesion Detection |
| S013 | Boutet et al., 2021 | PD stimulation | fMRI + clinical | DBS cohort | Not reported | Predicting optimal deep brain stimulation parameters for Parkinson's disease using functional MRI and machine learning |
| S014 | Brugnara et al., 2020 | Stroke / cerebrovascular disease | Multimodal | Not reported | Not reported | Multimodal Predictive Modeling of Endovascular Treatment Outcome for Acute Ischemic Stroke Using Machine-Learning |
| S015 | Calabrese et al., 2020 | Glioblastoma | Preoperative MRI | Institutional/TCIA imaging | Not reported | A fully automated artificial intelligence method for non-invasive, imaging-based identification of genetic alterations in glioblastomas |
| S016 | Camacho et al., 2023 | PD | T1 MRI | 2,041 scans; 13 studies | 2,041, 13 | Explainable classification of Parkinson's disease using deep learning trained on a large multi-center database of T1-weighted MRI datasets |
| S017 | Cao et al., 2020 | Parkinson disease / parkinsonism | Brain imaging modality Not reported | PPMI | Not reported | A Radiomics Approach to Predicting Parkinson's Disease by Incorporating Whole-Brain Functional Activity and Gray Matter Structure |
| S018 | Carré et al., 2020 | Cross-disease or general neuroimaging | MRI / diffusion | Not reported | Not reported | Standardization of brain MR images across machines and protocols: bridging the gap for MRI-based radiomics |
| S019 | Castellazzi et al., 2020 | Alzheimer disease / dementia | MRI / diffusion | Not reported | Not reported | A Machine Learning Approach for the Differential Diagnosis of Alzheimer and Vascular Dementia Fed by MRI Selected Features |
| S020 | Cheng et al., 2022 | Brain tumor / glioma | MRI / diffusion | BraTS / TCGA-TCIA | Not reported | A Fully Automated Multimodal MRI-Based Multi-Task Learning for Glioma Segmentation and IDH Genotyping |
| S021 | Choi et al., 2021 | Glioma | Multimodal MRI | 1,166 scans; 2 external sets | 1,166, 2 | Fully automated hybrid approach to predict the IDH mutation status of gliomas via deep learning and radiomics |
| S022 | Chougar et al., 2021 | Parkinsonism | MRI + DTI | 322 subjects; clinical replication n=143 | 322, 143 | Automated Categorization of Parkinsonian Syndromes Using Magnetic Resonance Imaging in a Clinical Setting |
| S023 | Clèrigues et al., 2020 | Stroke / cerebrovascular disease | MRI / diffusion | ISLES; ATLAS | Not reported | Acute and sub-acute stroke lesion segmentation from multimodal MRI |
| S024 | Conte et al., 2021 | Brain tumor / glioma | MRI / diffusion | BraTS / TCGA-TCIA | Not reported | Generative Adversarial Networks to Synthesize Missing T1 and FLAIR MRI Sequences for Use in a Multisequence Brain Tumor Segmentation Model |
| S025 | Dinsdale et al., 2021 | Cross-disease or general neuroimaging | MRI / diffusion | Not reported | Not reported | Deep learning-based unlearning of dataset bias for MRI harmonisation and confound removal |
| S026 | Duc et al., 2020 | Alzheimer disease / dementia | MRI / diffusion; fMRI | Not reported | Not reported | 3D-Deep Learning Based Automatic Diagnosis of Alzheimer's Disease with Joint MMSE Prediction Using Resting-State fMRI |
| S027 | El-Sappagh et al., 2021 | Alzheimer disease / dementia | Brain imaging modality Not reported | ADNI | Not reported | A multilayer multimodal detection and prediction model based on explainable artificial intelligence for Alzheimer's disease |
| S028 | Elijovich et al., 2022 | Stroke / cerebrovascular disease | Brain imaging modality Not reported | Not reported | Not reported | Automated emergent large vessel occlusion detection by artificial intelligence improves stroke workflow in a hub and spoke stroke system of care |
| S029 | Eshaghi et al., 2021 | Multiple sclerosis / demyelinating disease | MRI / diffusion | ENIGMA | Not reported | Identifying multiple sclerosis subtypes using unsupervised machine learning and MRI data |
| S030 | Finck et al., 2020 | Multiple sclerosis / demyelinating disease | Brain imaging modality Not reported | MSSEG / ISBI MS | Not reported | Deep-Learning Generated Synthetic Double Inversion Recovery Images Improve Multiple Sclerosis Lesion Detection |
| S031 | Flanders et al., 2020 | Stroke / cerebrovascular disease | CT / CTA / CTP | RSNA ICH Challenge | Not reported | Construction of a Machine Learning Dataset through Collaboration: The RSNA 2019 Brain CT Hemorrhage Challenge |
| S032 | Fürbass et al., 2020 | Epilepsy | EEG | Not reported | Not reported | An artificial intelligence-based EEG algorithm for detection of epileptiform EEG discharges: Validation against the diagnostic gold standard |
| S033 | Gabeff et al., 2021 | Epilepsy | EEG | Not reported | Not reported | Interpreting deep learning models for epileptic seizure detection on EEG signals |
| S034 | Gill et al., 2021 | Epilepsy/FCD | T1 + FLAIR MRI | 148 multicenter; 23 independent | 148, 23 | Multicenter Validation of a Deep Learning Detection Algorithm for Focal Cortical Dysplasia |
| S035 | Gleichgerrcht et al., 2021 | Epilepsy | MRI / diffusion | ENIGMA | Not reported | Artificial intelligence for classification of temporal lobe epilepsy with ROI-level MRI data: A worldwide ENIGMA-Epilepsy study |
| S036 | Gros et al., 2021 | Multiple sclerosis | Brain imaging modality Not reported | MSSEG / ISBI MS | Not reported | SoftSeg: Advantages of soft versus binary training for image segmentation |
| S037 | Hakim et al., 2021 | Stroke | CT perfusion | ISLES challenge | Not reported | Predicting Infarct Core From Computed Tomography Perfusion in Acute Ischemia With Machine Learning: Lessons From the ISLES Challenge |
| S038 | Henschel et al., 2020 | Normal aging and dementia | Brain imaging modality Not reported | OASIS | Not reported | FastSurfer - A fast and accurate deep learning based neuroimaging pipeline |
| S039 | Hollon et al., 2020 | Brain tumor | Stimulated Raman histology | 278-patient prospective multicenter trial | 278 | Near real-time intraoperative brain tumor diagnosis using stimulated Raman histology and deep neural networks |
| S040 | Hollon et al., 2023 | Brain tumor / glioma | Optical / histology imaging | Not reported | Not reported | Artificial-intelligence-based molecular classification of diffuse gliomas using rapid, label-free optical imaging |
| S041 | Hosseinzadeh et al., 2023 | Alzheimer disease / dementia | PET / SPECT | PPMI | Not reported | Prediction of Cognitive Decline in Parkinson's Disease Using Clinical and DAT SPECT Imaging Features, and Hybrid Machine Learning Systems |
| S042 | Ingalhalikar et al., 2021 | ASD | Resting-state fMRI | ABIDE multisite | Not reported | Functional Connectivity-Based Prediction of Autism on Site Harmonized ABIDE Dataset |
| S043 | Jayachandran Preetha et al., 2021 | Cross-disease or general neuroimaging | MRI / diffusion; PET / SPECT | Not reported | Not reported | Deep-learning-based synthesis of post-contrast T1-weighted MRI for tumour response assessment in neuro-oncology: a multicentre, retrospective cohort study |
| S044 | Jiang et al., 2022 | Brain tumor / glioma | Brain imaging modality Not reported | BraTS / TCGA-TCIA | Not reported | SwinBTS: A Method for 3D Multimodal Brain Tumor Segmentation Using Swin Transformer |
| S045 | Kamraoui et al., 2022 | Multiple sclerosis / demyelinating disease | FLAIR / T2 MRI | MSSEG / ISBI MS | Not reported | DeepLesionBrain: Towards a broader deep-learning generalization for multiple sclerosis lesion segmentation |
| S046 | Kim et al., 2020 | Multiple sclerosis / demyelinating disease | PET / SPECT | Not reported | Not reported | Deep Learning-Based Method to Differentiate Neuromyelitis Optica Spectrum Disorder From Multiple Sclerosis |
| S047 | Kumar et al., 2020 | Stroke / cerebrovascular disease | Brain imaging modality Not reported | ISLES | Not reported | CSNet: A new DeepNet framework for ischemic stroke lesion segmentation |
| S048 | Kural et al., 2022 | Epilepsy | CT / CTA / CTP; EEG | Not reported | Not reported | Accurate identification of EEG recordings with interictal epileptiform discharges using a hybrid approach: Artificial intelligence supervised by human experts |
| S049 | La Rosa et al., 2020 | MS | FLAIR + MP2RAGE | 90 patients; 2 centers | 90, 2 | Multiple sclerosis cortical and WM lesion segmentation at 3T MRI: a deep learning method based on FLAIR and MP2RAGE |
| S050 | Lanka et al., 2020 | Normative populations | Brain imaging modality Not reported | HCP | Not reported | Supervised machine learning for diagnostic classification from large-scale neuroimaging datasets |
| S051 | Larivière et al., 2020 | Epilepsy | MRI / diffusion | Not reported | Not reported | Functional connectome contractions in temporal lobe epilepsy: Microstructural underpinnings and predictors of surgical outcome |
| S052 | Lee et al., 2022 | Epilepsy | Structural MRI | Multicenter cohort | Not reported | Decomposing MRI phenotypic heterogeneity in epilepsy: a step towards personalized classification |
| S053 | Lee et al., 2023 | Stroke / cerebrovascular disease | Brain imaging modality Not reported | ATLAS | Not reported | Prediction of post-stroke cognitive impairment after acute ischemic stroke using machine learning |
| S054 | Li et al., 2021 | Stroke, TBI, aneurysm; Intracranial hemorrhage | CT / CTA / CTP | RSNA ICH Challenge | Not reported | Deep Learning for Hemorrhagic Lesion Detection and Segmentation on Brain CT Images |
| S055 | Liu et al., 2021 | Stroke | DWI MRI | 2,348 development; 280 external | 2,348, 280 | Deep learning-based detection and segmentation of diffusion abnormalities in acute ischemic stroke |
| S056 | Liu et al., 2022 | AD | Structural MRI | Multi-cohort | Not reported | Generalizable deep learning model for early Alzheimer's disease detection from structural MRIs |
| S057 | Magesh et al., 2020 | Parkinson disease / parkinsonism | PET / SPECT | PPMI | Not reported | An Explainable Machine Learning Model for Early Detection of Parkinson's Disease using LIME on DaTSCAN Imagery |
| S058 | Maggi et al., 2020 | Multiple sclerosis / demyelinating disease | Brain imaging modality Not reported | MSSEG / ISBI MS | Not reported | CVSnet: A machine learning approach for automated central vein sign assessment in multiple sclerosis |
| S059 | Martinez-Gutierrez et al., 2023 | Stroke / cerebrovascular disease | CT / CTA / CTP | Not reported | Not reported | Automated Large Vessel Occlusion Detection Software and Thrombectomy Treatment Times: A Cluster Randomized Clinical Trial |
| S060 | Matsoukas et al., 2023 | Stroke | CTA | 1,822 prospective CTAs | 1,822 | AI software detection of large vessel occlusion stroke on CT angiography: a real-world prospective diagnostic test accuracy study |
| S061 | Monteiro et al., 2020 | TBI | Head CT | 839 multicenter scans; 500 external | 839, 500 | Multiclass semantic segmentation and quantification of traumatic brain injury lesions on head CT using deep learning: an algorithm development and multicentre validation study |
| S062 | Muckley et al., 2021 | MRI reconstruction | MRI / diffusion | fastMRI | Not reported | Results of the 2020 fastMRI Challenge for Machine Learning MR Image Reconstruction |
| S063 | Mårtensson et al., 2020 | Aging and AD; Normal aging and dementia | MRI / diffusion | AIBL; OASIS | Not reported | The reliability of a deep learning model in clinical out-of-distribution MRI data: A multicohort study |
| S064 | Naser et al., 2020 | Lower-grade glioma | MRI | Public clinical images | Not reported | Brain tumor segmentation and grading of lower-grade glioma using deep learning in MRI images |
| S065 | Nishi et al., 2020 | Stroke / cerebrovascular disease | DWI / perfusion MRI | ISLES | Not reported | Deep Learning-Derived High-Level Neuroimaging Features Predict Clinical Outcomes for Large Vessel Occlusion |
| S066 | Pan et al., 2020 | AD | MRI | Public MRI cohort | Not reported | Early Detection of Alzheimer's Disease Using Magnetic Resonance Imaging: A Novel Approach Combining Convolutional Neural Networks and Ensemble Learning |
| S067 | Park et al., 2021 | Autism spectrum disorder | MRI / diffusion | HCP | Not reported | Differences in subcortico-cortical interactions identified from connectome and microcircuit models in autism |
| S068 | Pavel et al., 2020 | Epilepsy | EEG | Not reported | Not reported | A machine-learning algorithm for neonatal seizure recognition: a multicentre, randomised, controlled trial |
| S069 | Pease et al., 2022 | Traumatic brain injury | CT / CTA / CTP | Not reported | Not reported | Outcome Prediction in Patients with Severe Traumatic Brain Injury Using Deep Learning from Head CT Scans |
| S070 | Pei et al., 2020 | Brain tumor / glioma | Brain imaging modality Not reported | BraTS / TCGA-TCIA | Not reported | Context aware deep learning for brain tumor segmentation, subtype classification, and survival prediction using radiology images |
| S071 | Peng et al., 2021 | Population cohort | Brain imaging modality Not reported | UK Biobank imaging | Not reported | Accurate brain age prediction with lightweight deep neural networks |
| S072 | Pinaya et al., 2021 | Alzheimer disease / dementia | Brain imaging modality Not reported | ADNI | Not reported | Using normative modelling to detect disease progression in mild cognitive impairment and Alzheimer's disease in a cross-sectional multi-cohort study |
| S073 | Popuri et al., 2020 | Alzheimer disease / dementia | MRI / diffusion | ADNI; AIBL; OASIS | Not reported | Using machine learning to quantify structural MRI neurodegeneration patterns of Alzheimer's disease into dementia score: Independent validation on 8,834 images from ADNI, AIBL, OASIS, and MIRIAD databases |
| S074 | Qiu et al., 2020a | AD | MRI + clinical | 417 development; 1,066 across 3 external cohorts | 417, 1,066, 3 | Development and validation of an interpretable deep learning framework for Alzheimer's disease classification |
| S075 | Qiu et al., 2020b | Stroke | Non-contrast CT | Acute ischemic stroke | Not reported | Machine Learning for Detecting Early Infarction in Acute Stroke with Non-Contrast-enhanced CT |
| S076 | Qiu et al., 2022 | Dementia | Multimodal clinical + imaging | Multi-cohort | Not reported | Multimodal deep learning for Alzheimer's disease dementia assessment |
| S077 | Radua et al., 2020 | Cross-disorder consortia | MRI / diffusion | ENIGMA | Not reported | Increased power by harmonizing structural MRI site differences with the ComBat batch adjustment method in ENIGMA |
| S078 | Rakić et al., 2021 | Multiple sclerosis / demyelinating disease | Brain imaging modality Not reported | Not reported | Not reported | icobrain ms 5.1: Combining unsupervised and supervised approaches for improving the detection of multiple sclerosis lesions |
| S079 | Ranjbarzadeh et al., 2021 | Brain tumor / glioma | MRI / diffusion | BraTS / TCGA-TCIA | Not reported | Brain tumor segmentation based on deep learning and an attention mechanism using MRI multi-modalities brain images |
| S080 | Roca et al., 2020 | Multiple sclerosis / demyelinating disease | MRI / diffusion | ISLES | Not reported | Artificial intelligence to predict clinical disability in patients with multiple sclerosis using FLAIR MRI |
| S081 | Seyam et al., 2022 | Stroke / cerebrovascular disease | CT / CTA / CTP | RSNA ICH Challenge | Not reported | Utilization of Artificial Intelligence-based Intracranial Hemorrhage Detection on Emergent Noncontrast CT Images in Clinical Workflow |
| S082 | Shi et al., 2020 | Stroke / cerebrovascular disease | CT / CTA / CTP | Not reported | Not reported | A clinically applicable deep-learning model for detecting intracranial aneurysm in computed tomography angiography images |
| S083 | Shin et al., 2021 | Alzheimer disease / dementia | MRI / diffusion | PPMI | Not reported | Cortical Thickness from MRI to Predict Conversion from Mild Cognitive Impairment to Dementia in Parkinson Disease: A Machine Learning-based Model |
| S084 | Shu et al., 2021 | Parkinson disease / parkinsonism | MRI / diffusion | PPMI | Not reported | Predicting the progression of Parkinson's disease using conventional MRI and machine learning: An application of radiomic biomarkers in whole-brain white matter |
| S085 | Solana-Lavalle et al., 2021 | Parkinson disease / parkinsonism | MRI / diffusion | PPMI | Not reported | Classification of PPMI MRI scans with voxel-based morphometry and machine learning to assist in the diagnosis of Parkinson's disease |
| S086 | Song et al., 2021 | Alzheimer disease / dementia | MRI / diffusion; PET / SPECT | ADNI | Not reported | An Effective Multimodal Image Fusion Method Using MRI and PET for Alzheimer's Disease Diagnosis |
| S087 | Spitzer et al., 2022 | Epilepsy/FCD | Surface MRI features | 1,015 participants; 22 centers | 1,015, 22 | Interpretable surface-based detection of focal cortical dysplasias: a Multi-centre Epilepsy Lesion Detection study |
| S088 | Spooner et al., 2020 | Alzheimer disease / dementia | Brain imaging modality Not reported | Not reported | Not reported | A comparison of machine learning methods for survival analysis of high-dimensional clinical data for dementia prediction |
| S089 | Steyaert et al., 2023 | Brain tumor / glioma | Brain imaging modality Not reported | BraTS / TCGA-TCIA | Not reported | Multimodal deep learning to predict prognosis in adult and pediatric brain tumors |
| S090 | Stib et al., 2020 | Stroke / cerebrovascular disease | CT / CTA / CTP | Not reported | Not reported | Detecting Large Vessel Occlusion at Multiphase CT Angiography by Using a Deep Convolutional Neural Network |
| S091 | Storelli et al., 2022 | MS | Longitudinal MRI | Clinical cohort | Not reported | A Deep Learning Approach to Predicting Disease Progression in Multiple Sclerosis Using Magnetic Resonance Imaging |
| S092 | Storelli et al., 2026 | Multiple sclerosis / demyelinating disease | MRI / diffusion | Not reported | Not reported | Explainable Artificial Intelligence to Predict Neurocognitive Disorder Progression in Multiple Sclerosis Using MRI and Clinical Data |
| S093 | Sun et al., 2024 | PD vs MSA | PET/MRI + clinical | 119 participants | 119 | Identification of Parkinson's disease and multiple system atrophy using multimodal PET/MRI radiomics |
| S094 | Sunwoo et al., 2026 | Stroke / cerebrovascular disease | Brain imaging modality Not reported | Not reported | Not reported | AI assisted detection of large vessel occlusion on non-contrast CT: multinational validation and reader study |
| S095 | Talai et al., 2021 | Parkinson disease / parkinsonism | MRI / diffusion | Not reported | Not reported | Utility of Multi-Modal MRI for Differentiating of Parkinson's Disease and Progressive Supranuclear Palsy Using Machine Learning |
| S096 | Tang et al., 2026 | Alzheimer disease / dementia | PET / SPECT | Not reported | Not reported | Decoupling Alzheimer Disease Pathologic Abnormalities at PET with Improved Clinical Relevance by Interpretable Adversarial Decomposition Learning |
| S097 | Tanno et al., 2021 | Cross-disease or general neuroimaging | MRI / diffusion | Not reported | Not reported | Uncertainty modelling in deep learning for safer neuroimage enhancement: Demonstration in diffusion MRI |
| S098 | Thakur et al., 2020 | Brain tumor / glioma | MRI / diffusion | BraTS / TCGA-TCIA | Not reported | Brain extraction on MRI scans in presence of diffuse glioma: Multi-institutional performance evaluation of deep learning methods and robust modality-agnostic training |
| S099 | Theocharidis et al., 2026 | Alzheimer disease / dementia | MRI / diffusion | ADNI | Not reported | Applying ensemble machine learning techniques to MRI scans to predict Alzheimer's disease |
| S100 | Thomas et al., 2020 | Autism spectrum disorder | MRI / diffusion; PET / SPECT | ABIDE | Not reported | Classifying Autism Spectrum Disorder Using the Temporal Statistics of Resting-State Functional MRI Data With 3D Convolutional Neural Networks |
| S101 | Thomas et al., 2026 | Stroke / cerebrovascular disease | CT / CTA / CTP | Not reported | Not reported | Accuracy of Automated Deep Learning versus Expert Clinicians for Diagnosis of Acute Lacunar Stroke on CT Perfusion |
| S102 | Tveit et al., 2023 | Epilepsy and diffuse dysfunction | EEG | Not reported | Not reported | Automated Interpretation of Clinical Electroencephalograms Using Artificial Intelligence |
| S103 | van der Voort et al., 2023 | Glioma | 3D structural MRI | 1,508 development; 240 external | 1,508, 240 | Combined molecular subtyping, grading, and segmentation of glioma using multi-task deep learning |
| S104 | Venugopalan et al., 2021 | Alzheimer disease / dementia | Multimodal | ADNI | Not reported | Multimodal deep learning models for early detection of Alzheimer's disease stage |
| S105 | Wen et al., 2020 | AD | T1 MRI | ADNI, AIBL, OASIS | Not reported | Convolutional neural networks for classification of Alzheimer's disease: Overview and reproducible evaluation |
| S106 | Wen et al., 2022 | Autism spectrum disorder | PET / SPECT | ABIDE | Not reported | MVS-GCN: A prior brain structure learning-guided multi-view graph convolution network for autism spectrum disorder diagnosis |
| S107 | Wiltgen et al., 2024 | MS | T1 + FLAIR | 491 training; 103 public tests | 491, 103 | LST-AI: A deep learning ensemble for accurate MS lesion segmentation |
| S108 | Wu et al., 2022 | Parkinson disease / parkinsonism | Brain imaging modality Not reported | Not reported | Not reported | Differential Diagnosis of Parkinsonism Based on Deep Metabolic Imaging Indices |
| S109 | Xiao et al., 2023 | Epilepsy | MRI / diffusion | Not reported | Not reported | Identification of different MRI atrophy progression trajectories in epilepsy by subtype and stage inference |
| S110 | Xue et al., 2024 | Dementia | Multimodal | 51,269 participants; 9 datasets | 51,269, 9 | AI-based differential diagnosis of dementia etiologies on multimodal data |
| S111 | Yaman et al., 2020 | MRI reconstruction | Brain imaging modality Not reported | fastMRI | Not reported | Self-supervised learning of physics-guided reconstruction neural networks without fully sampled reference data |
| S112 | Yang et al., 2021 | Alzheimer disease / dementia | Brain imaging modality Not reported | ADNI | Not reported | A deep learning framework identifies dimensional representations of Alzheimer's Disease from brain structure |
| S113 | Yasaka et al., 2021 | Parkinson disease / parkinsonism | MRI / diffusion | Not reported | Not reported | Parkinson's disease: deep learning with a parameter-weighted structural connectome matrix for diagnosis and neural circuit disorder investigation |
| S114 | Ye et al., 2020 | Multiple sclerosis / demyelinating disease | MRI / diffusion; PET / SPECT | Not reported | Not reported | Deep learning with diffusion basis spectrum imaging for classification of multiple sclerosis lesions |
| S115 | Yin et al., 2023 | Dementia, tumors, PD, epilepsy; Normative populations | Structural MRI | HCP; UK Biobank imaging | Not reported | Anatomically interpretable deep learning of brain age captures domain-specific cognitive impairment |
| S116 | Yogananda et al., 2021 | Brain tumor / glioma | MRI / diffusion | BraTS / TCGA-TCIA | Not reported | MRI-Based Deep-Learning Method for Determining Glioma MGMT Promoter Methylation Status |
| S117 | Yu et al., 2020 | Stroke | Baseline DWI/perfusion MRI | 182 patients | 182 | Use of Deep Learning to Predict Final Ischemic Stroke Lesions From Initial Magnetic Resonance Imaging |
| S118 | Zhao et al., 2020 | Multiple sclerosis / demyelinating disease | Brain imaging modality Not reported | Not reported | Not reported | Ensemble learning predicts multiple sclerosis disease course in the SUMMIT study |
| S119 | Zhao et al., 2022 | Parkinson disease / parkinsonism | PET / SPECT | PPMI | Not reported | Decoding the dopamine transporter imaging for the differential diagnosis of parkinsonism using deep learning |
| S120 | Zhou et al., 2021 | MRI reconstruction | Brain imaging modality Not reported | fastMRI | Not reported | Models Genesis |
| S121 | Zhu et al., 2021a | Stroke / cerebrovascular disease | MRI / diffusion | ISLES | Not reported | An automatic machine learning approach for ischemic stroke onset time identification based on DWI and FLAIR imaging |
| S122 | Zhu et al., 2021b | Alzheimer disease / dementia | MRI / diffusion | ADNI | Not reported | Dual Attention Multi-Instance Deep Learning for Alzheimer's Disease Diagnosis With Structural MRI |
| S123 | Zhuge et al., 2020 | Brain tumor / glioma | MRI / diffusion | BraTS / TCGA-TCIA | Not reported | Automated glioma grading on conventional MRI images using deep convolutional neural networks |

**Table S1b. Eligible study model, validation, outcome, performance, external-validation, and limitation fields (N = 123)**

| **ID** | **Model / task** | **Comparator** | **Validation design** | **Outcome / task** | **Performance estimate** | **External validation** | **Principal limitation** |
| --- | --- | --- | --- | --- | --- | --- | --- |
| S001 | prediction / prognosis | Not reported; not inferred | Not reported; not inferred | Task identified from title or retained mapping: prediction / prognosis | Not reported; not inferred | Not reported; not inferred | Comparator, validation, performance, and external-validation fields were unavailable in the retained structured extraction and were not inferred. |
| S002 | Study-specific AI/CAD task | Not reported; not inferred | Not reported; not inferred | Task identified from title or retained mapping: Study-specific AI/CAD task | Not reported; not inferred | Not reported; not inferred | Comparator, validation, performance, and external-validation fields were unavailable in the retained structured extraction and were not inferred. |
| S003 | prediction / prognosis | Not reported; not inferred | Not reported; not inferred | Task identified from title or retained mapping: prediction / prognosis | Not reported; not inferred | Not reported; not inferred | Comparator, validation, performance, and external-validation fields were unavailable in the retained structured extraction and were not inferred. |
| S004 | Autoencoders; Study-specific AI/CAD task | Not reported; not inferred | Not reported; not inferred | Task identified from title or retained mapping: Study-specific AI/CAD task | Not reported; not inferred | Not reported; not inferred | Comparator, validation, performance, and external-validation fields were unavailable in the retained structured extraction and were not inferred. |
| S005 | Multimodal / emerging; prediction / prognosis | Not reported; not inferred | Not reported; not inferred | Task identified from title or retained mapping: prediction / prognosis | Not reported; not inferred | Not reported; not inferred | Comparator, validation, performance, and external-validation fields were unavailable in the retained structured extraction and were not inferred. |
| S006 | U-Net family; detection / segmentation | Not reported; not inferred | Not reported; not inferred | Task identified from title or retained mapping: detection / segmentation | Not reported; not inferred | Not reported; not inferred | Comparator, validation, performance, and external-validation fields were unavailable in the retained structured extraction and were not inferred. |
| S007 | 3D CNN; Study-specific AI/CAD task | Not reported; not inferred | Not reported; not inferred | Task identified from title or retained mapping: Study-specific AI/CAD task | Not reported; not inferred | Not reported; not inferred | Comparator, validation, performance, and external-validation fields were unavailable in the retained structured extraction and were not inferred. |
| S008 | Autoencoders; detection / segmentation | Not reported; not inferred | Not reported; not inferred | Task identified from title or retained mapping: detection / segmentation | Not reported; not inferred | Not reported; not inferred | Comparator, validation, performance, and external-validation fields were unavailable in the retained structured extraction and were not inferred. |
| S009 | Study-specific AI/CAD task | Not reported; not inferred | Not reported; not inferred | Task identified from title or retained mapping: Study-specific AI/CAD task | Not reported; not inferred | Not reported; not inferred | Comparator, validation, performance, and external-validation fields were unavailable in the retained structured extraction and were not inferred. |
| S010 | Transformers; prediction / prognosis | Not reported; not inferred | Not reported; not inferred | Task identified from title or retained mapping: prediction / prognosis | Not reported; not inferred | Not reported; not inferred | Comparator, validation, performance, and external-validation fields were unavailable in the retained structured extraction and were not inferred. |
| S011 | detection / segmentation | Not reported; not inferred | Not reported; not inferred | Task identified from title or retained mapping: detection / segmentation | Not reported; not inferred | Not reported; not inferred | Comparator, validation, performance, and external-validation fields were unavailable in the retained structured extraction and were not inferred. |
| S012 | detection / segmentation | Not reported; not inferred | Not reported; not inferred | Task identified from title or retained mapping: detection / segmentation | Not reported; not inferred | Not reported; not inferred | Comparator, validation, performance, and external-validation fields were unavailable in the retained structured extraction and were not inferred. |
| S013 | ML parameter prediction | Not reported; not inferred | Out-of-sample prediction | Task identified from title or retained mapping: prediction / prognosis | Functional imaging supported individualized estimation of effective stimulation settings. | Not established in retained structured extraction | Validation and performance fields reproduce the retained structured extraction; no standardized aggregate category was assigned for this revision. |
| S014 | Multimodal / emerging; prediction / prognosis | Not reported; not inferred | Not reported; not inferred | Task identified from title or retained mapping: prediction / prognosis | Not reported; not inferred | Not reported; not inferred | Comparator, validation, performance, and external-validation fields were unavailable in the retained structured extraction and were not inferred. |
| S015 | Automated genetic-alteration prediction | Not reported; not inferred | Cross-validation | Task identified from title or retained mapping: classification / differential diagnosis | Demonstrated noninvasive prediction of selected molecular alterations from imaging. | Not established in retained structured extraction | Validation and performance fields reproduce the retained structured extraction; no standardized aggregate category was assigned for this revision. |
| S016 | Explainable CNN classification | Not reported; not inferred | Independent test AUC | Task identified from title or retained mapping: classification / differential diagnosis | Accuracy 79.3% and AUC 0.87 with similar independent performance. | Not established in retained structured extraction | Validation and performance fields reproduce the retained structured extraction; no standardized aggregate category was assigned for this revision. |
| S017 | prediction / prognosis | Not reported; not inferred | Not reported; not inferred | Task identified from title or retained mapping: prediction / prognosis | Not reported; not inferred | Not reported; not inferred | Comparator, validation, performance, and external-validation fields were unavailable in the retained structured extraction and were not inferred. |
| S018 | Handcrafted ML; Study-specific AI/CAD task | Not reported; not inferred | Not reported; not inferred | Task identified from title or retained mapping: Study-specific AI/CAD task | Not reported; not inferred | Not reported; not inferred | Comparator, validation, performance, and external-validation fields were unavailable in the retained structured extraction and were not inferred. |
| S019 | Handcrafted ML; classification / differential diagnosis | Not reported; not inferred | Not reported; not inferred | Task identified from title or retained mapping: classification / differential diagnosis | Not reported; not inferred | Not reported; not inferred | Comparator, validation, performance, and external-validation fields were unavailable in the retained structured extraction and were not inferred. |
| S020 | Transformers; detection / segmentation | Not reported; not inferred | Not reported; not inferred | Task identified from title or retained mapping: detection / segmentation | Not reported; not inferred | Not reported; not inferred | Comparator, validation, performance, and external-validation fields were unavailable in the retained structured extraction and were not inferred. |
| S021 | CNN-radiomics IDH prediction | Not reported; not inferred | Dice and external AUC | Task identified from title or retained mapping: prediction / prognosis | Segmentation Dice 0.86-0.92; IDH AUC declined from 0.96 internal to 0.86 external. | Reported in retained structured extraction | Validation and performance fields reproduce the retained structured extraction; no standardized aggregate category was assigned for this revision. |
| S022 | Supervised differential classification | Not reported; not inferred | Balanced accuracy/AUC | Task identified from title or retained mapping: Study-specific AI/CAD task | Replication AUC 0.907-0.995 for several pairwise syndromic comparisons. | Not established in retained structured extraction | Validation and performance fields reproduce the retained structured extraction; no standardized aggregate category was assigned for this revision. |
| S023 | U-Net family; detection / segmentation | Not reported; not inferred | Not reported; not inferred | Task identified from title or retained mapping: detection / segmentation | Not reported; not inferred | Not reported; not inferred | Comparator, validation, performance, and external-validation fields were unavailable in the retained structured extraction and were not inferred. |
| S024 | Generative models; detection / segmentation; reconstruction / synthesis / harmonization | Not reported; not inferred | Not reported; not inferred | Task identified from title or retained mapping: detection / segmentation; reconstruction / synthesis / harmonization | Not reported; not inferred | Not reported; not inferred | Comparator, validation, performance, and external-validation fields were unavailable in the retained structured extraction and were not inferred. |
| S025 | reconstruction / synthesis / harmonization | Not reported; not inferred | Not reported; not inferred | Task identified from title or retained mapping: reconstruction / synthesis / harmonization | Not reported; not inferred | Not reported; not inferred | Comparator, validation, performance, and external-validation fields were unavailable in the retained structured extraction and were not inferred. |
| S026 | 3D CNN; classification / differential diagnosis; prediction / prognosis | Not reported; not inferred | Not reported; not inferred | Task identified from title or retained mapping: classification / differential diagnosis; prediction / prognosis | Not reported; not inferred | Not reported; not inferred | Comparator, validation, performance, and external-validation fields were unavailable in the retained structured extraction and were not inferred. |
| S027 | Attention networks; detection / segmentation; prediction / prognosis | Not reported; not inferred | Not reported; not inferred | Task identified from title or retained mapping: detection / segmentation; prediction / prognosis | Not reported; not inferred | Not reported; not inferred | Comparator, validation, performance, and external-validation fields were unavailable in the retained structured extraction and were not inferred. |
| S028 | detection / segmentation | Not reported; not inferred | Not reported; not inferred | Task identified from title or retained mapping: detection / segmentation | Not reported; not inferred | Not reported; not inferred | Comparator, validation, performance, and external-validation fields were unavailable in the retained structured extraction and were not inferred. |
| S029 | Study-specific AI/CAD task | Not reported; not inferred | Not reported; not inferred | Task identified from title or retained mapping: Study-specific AI/CAD task | Not reported; not inferred | Not reported; not inferred | Comparator, validation, performance, and external-validation fields were unavailable in the retained structured extraction and were not inferred. |
| S030 | Generative models; detection / segmentation; reconstruction / synthesis / harmonization | Not reported; not inferred | Not reported; not inferred | Task identified from title or retained mapping: detection / segmentation; reconstruction / synthesis / harmonization | Not reported; not inferred | Not reported; not inferred | Comparator, validation, performance, and external-validation fields were unavailable in the retained structured extraction and were not inferred. |
| S031 | Study-specific AI/CAD task | Not reported; not inferred | Not reported; not inferred | Task identified from title or retained mapping: Study-specific AI/CAD task | Not reported; not inferred | Not reported; not inferred | Comparator, validation, performance, and external-validation fields were unavailable in the retained structured extraction and were not inferred. |
| S032 | RNN / temporal models; detection / segmentation; classification / differential diagnosis | Not reported; not inferred | Not reported; not inferred | Task identified from title or retained mapping: detection / segmentation; classification / differential diagnosis | Not reported; not inferred | Not reported; not inferred | Comparator, validation, performance, and external-validation fields were unavailable in the retained structured extraction and were not inferred. |
| S033 | RNN / temporal models; detection / segmentation | Not reported; not inferred | Not reported; not inferred | Task identified from title or retained mapping: detection / segmentation | Not reported; not inferred | Not reported; not inferred | Comparator, validation, performance, and external-validation fields were unavailable in the retained structured extraction and were not inferred. |
| S034 | CNN detection + uncertainty | Not reported; not inferred | Sensitivity/specificity | Task identified from title or retained mapping: detection / segmentation | Sensitivity 93% in leave-one-site-out analysis and 83% independently. | Not established in retained structured extraction | Validation and performance fields reproduce the retained structured extraction; no standardized aggregate category was assigned for this revision. |
| S035 | Support vector machines; classification / differential diagnosis | Not reported; not inferred | Not reported; not inferred | Task identified from title or retained mapping: classification / differential diagnosis | Not reported; not inferred | Not reported; not inferred | Comparator, validation, performance, and external-validation fields were unavailable in the retained structured extraction and were not inferred. |
| S036 | U-Net family; detection / segmentation | Not reported; not inferred | Not reported; not inferred | Task identified from title or retained mapping: detection / segmentation | Not reported; not inferred | Not reported; not inferred | Comparator, validation, performance, and external-validation fields were unavailable in the retained structured extraction and were not inferred. |
| S037 | ML infarct-core prediction | Not reported; not inferred | Challenge comparison | Task identified from title or retained mapping: prediction / prognosis | Highlighted cross-method lessons and the effect of reference definition on infarct-core estimates. | Not established in retained structured extraction | Validation and performance fields reproduce the retained structured extraction; no standardized aggregate category was assigned for this revision. |
| S038 | 3D CNN; Study-specific AI/CAD task | Not reported; not inferred | Not reported; not inferred | Task identified from title or retained mapping: Study-specific AI/CAD task | Not reported; not inferred | Not reported; not inferred | Comparator, validation, performance, and external-validation fields were unavailable in the retained structured extraction and were not inferred. |
| S039 | CNN intraoperative diagnosis | Not reported; not inferred | Accuracy; noninferiority | Task identified from title or retained mapping: classification / differential diagnosis | 94.6% CNN accuracy versus 93.9% conventional pathologist interpretation. | Not established in retained structured extraction | Validation and performance fields reproduce the retained structured extraction; no standardized aggregate category was assigned for this revision. |
| S040 | classification / differential diagnosis | Not reported; not inferred | Not reported; not inferred | Task identified from title or retained mapping: classification / differential diagnosis | Not reported; not inferred | Not reported; not inferred | Comparator, validation, performance, and external-validation fields were unavailable in the retained structured extraction and were not inferred. |
| S041 | prediction / prognosis | Not reported; not inferred | Not reported; not inferred | Task identified from title or retained mapping: prediction / prognosis | Not reported; not inferred | Not reported; not inferred | Comparator, validation, performance, and external-validation fields were unavailable in the retained structured extraction and were not inferred. |
| S042 | ComBat + connectivity classifiers | Not reported; not inferred | Cross-site accuracy | Task identified from title or retained mapping: prediction / prognosis; reconstruction / synthesis / harmonization | Harmonization improved classification across several models and reduced site effects. | Not established in retained structured extraction | Validation and performance fields reproduce the retained structured extraction; no standardized aggregate category was assigned for this revision. |
| S043 | Generative models; reconstruction / synthesis / harmonization | Not reported; not inferred | Not reported; not inferred | Task identified from title or retained mapping: reconstruction / synthesis / harmonization | Not reported; not inferred | Not reported; not inferred | Comparator, validation, performance, and external-validation fields were unavailable in the retained structured extraction and were not inferred. |
| S044 | Transformers; detection / segmentation | Not reported; not inferred | Not reported; not inferred | Task identified from title or retained mapping: detection / segmentation | Not reported; not inferred | Not reported; not inferred | Comparator, validation, performance, and external-validation fields were unavailable in the retained structured extraction and were not inferred. |
| S045 | U-Net family; detection / segmentation | Not reported; not inferred | Not reported; not inferred | Task identified from title or retained mapping: detection / segmentation | Not reported; not inferred | Not reported; not inferred | Comparator, validation, performance, and external-validation fields were unavailable in the retained structured extraction and were not inferred. |
| S046 | Support vector machines; classification / differential diagnosis | Not reported; not inferred | Not reported; not inferred | Task identified from title or retained mapping: classification / differential diagnosis | Not reported; not inferred | Not reported; not inferred | Comparator, validation, performance, and external-validation fields were unavailable in the retained structured extraction and were not inferred. |
| S047 | U-Net family; detection / segmentation | Not reported; not inferred | Not reported; not inferred | Task identified from title or retained mapping: detection / segmentation | Not reported; not inferred | Not reported; not inferred | Comparator, validation, performance, and external-validation fields were unavailable in the retained structured extraction and were not inferred. |
| S048 | classification / differential diagnosis | Not reported; not inferred | Not reported; not inferred | Task identified from title or retained mapping: classification / differential diagnosis | Not reported; not inferred | Not reported; not inferred | Comparator, validation, performance, and external-validation fields were unavailable in the retained structured extraction and were not inferred. |
| S049 | 3D U-Net lesion segmentation | Not reported; not inferred | Detection/false positives | Task identified from title or retained mapping: detection / segmentation | 76% detection for cortical and WM lesions with cross-center evaluation. | Not established in retained structured extraction | Validation and performance fields reproduce the retained structured extraction; no standardized aggregate category was assigned for this revision. |
| S050 | Handcrafted ML / Support vector machines; classification / differential diagnosis | Not reported; not inferred | Not reported; not inferred | Task identified from title or retained mapping: classification / differential diagnosis | Not reported; not inferred | Not reported; not inferred | Comparator, validation, performance, and external-validation fields were unavailable in the retained structured extraction and were not inferred. |
| S051 | Graph neural networks; prediction / prognosis | Not reported; not inferred | Not reported; not inferred | Task identified from title or retained mapping: prediction / prognosis | Not reported; not inferred | Not reported; not inferred | Comparator, validation, performance, and external-validation fields were unavailable in the retained structured extraction and were not inferred. |
| S052 | Unsupervised phenotype decomposition | Not reported; not inferred | Trajectory stability | Task identified from title or retained mapping: classification / differential diagnosis | Identified distinct atrophy progression trajectories across epilepsy subtypes. | Not established in retained structured extraction | Validation and performance fields reproduce the retained structured extraction; no standardized aggregate category was assigned for this revision. |
| S053 | prediction / prognosis | Not reported; not inferred | Not reported; not inferred | Task identified from title or retained mapping: prediction / prognosis | Not reported; not inferred | Not reported; not inferred | Comparator, validation, performance, and external-validation fields were unavailable in the retained structured extraction and were not inferred. |
| S054 | U-Net family; detection / segmentation | Not reported; not inferred | Not reported; not inferred | Task identified from title or retained mapping: detection / segmentation | Not reported; not inferred | Not reported; not inferred | Comparator, validation, performance, and external-validation fields were unavailable in the retained structured extraction and were not inferred. |
| S055 | Deep lesion detection/segmentation | Not reported; not inferred | External lesion metrics | Task identified from title or retained mapping: detection / segmentation | Improved small-lesion detection and retained performance under clinical heterogeneity. | Reported in retained structured extraction | Validation and performance fields reproduce the retained structured extraction; no standardized aggregate category was assigned for this revision. |
| S056 | Generalizable deep classification | Not reported; not inferred | External evaluation | Task identified from title or retained mapping: detection / segmentation | Emphasized transfer across structural-MRI cohorts rather than a single benchmark. | Reported in retained structured extraction | Validation and performance fields reproduce the retained structured extraction; no standardized aggregate category was assigned for this revision. |
| S057 | Attention networks; detection / segmentation | Not reported; not inferred | Not reported; not inferred | Task identified from title or retained mapping: detection / segmentation | Not reported; not inferred | Not reported; not inferred | Comparator, validation, performance, and external-validation fields were unavailable in the retained structured extraction and were not inferred. |
| S058 | Study-specific AI/CAD task | Not reported; not inferred | Not reported; not inferred | Task identified from title or retained mapping: Study-specific AI/CAD task | Not reported; not inferred | Not reported; not inferred | Comparator, validation, performance, and external-validation fields were unavailable in the retained structured extraction and were not inferred. |
| S059 | detection / segmentation | Not reported; not inferred | Not reported; not inferred | Task identified from title or retained mapping: detection / segmentation | Not reported; not inferred | Not reported; not inferred | Comparator, validation, performance, and external-validation fields were unavailable in the retained structured extraction and were not inferred. |
| S060 | Commercial LVO detection | Not reported; not inferred | Sensitivity/specificity/AUC | Task identified from title or retained mapping: detection / segmentation; classification / differential diagnosis | AUC 0.95 for ICA-T/M1, with reduced sensitivity for M2 occlusion. | Not established in retained structured extraction | Validation and performance fields reproduce the retained structured extraction; no standardized aggregate category was assigned for this revision. |
| S061 | Multiclass CNN segmentation | Not reported; not inferred | Volume agreement/detection | Task identified from title or retained mapping: detection / segmentation | Quantified hemorrhage and edema with multicenter and external validation. | Reported in retained structured extraction | Validation and performance fields reproduce the retained structured extraction; no standardized aggregate category was assigned for this revision. |
| S062 | Self-supervised / transfer; reconstruction / synthesis / harmonization | Not reported; not inferred | Not reported; not inferred | Task identified from title or retained mapping: reconstruction / synthesis / harmonization | Not reported; not inferred | Not reported; not inferred | Comparator, validation, performance, and external-validation fields were unavailable in the retained structured extraction and were not inferred. |
| S063 | Study-specific AI/CAD task | Not reported; not inferred | Not reported; not inferred | Task identified from title or retained mapping: Study-specific AI/CAD task | Not reported; not inferred | Not reported; not inferred | Comparator, validation, performance, and external-validation fields were unavailable in the retained structured extraction and were not inferred. |
| S064 | Deep segmentation and grading | Not reported; not inferred | Dice/classification metrics | Task identified from title or retained mapping: detection / segmentation | Combined tumor delineation with grade-related classification. | Not established in retained structured extraction | Validation and performance fields reproduce the retained structured extraction; no standardized aggregate category was assigned for this revision. |
| S065 | prediction / prognosis | Not reported; not inferred | Not reported; not inferred | Task identified from title or retained mapping: prediction / prognosis | Not reported; not inferred | Not reported; not inferred | Comparator, validation, performance, and external-validation fields were unavailable in the retained structured extraction and were not inferred. |
| S066 | CNN + ensemble classification | Not reported; not inferred | Internal accuracy | Task identified from title or retained mapping: detection / segmentation | Combined learned and ensemble evidence for early-stage classification. | Not established in retained structured extraction | Validation and performance fields reproduce the retained structured extraction; no standardized aggregate category was assigned for this revision. |
| S067 | Graph neural networks; Study-specific AI/CAD task | Not reported; not inferred | Not reported; not inferred | Task identified from title or retained mapping: Study-specific AI/CAD task | Not reported; not inferred | Not reported; not inferred | Comparator, validation, performance, and external-validation fields were unavailable in the retained structured extraction and were not inferred. |
| S068 | RNN / temporal models; Study-specific AI/CAD task | Not reported; not inferred | Not reported; not inferred | Task identified from title or retained mapping: Study-specific AI/CAD task | Not reported; not inferred | Not reported; not inferred | Comparator, validation, performance, and external-validation fields were unavailable in the retained structured extraction and were not inferred. |
| S069 | prediction / prognosis | Not reported; not inferred | Not reported; not inferred | Task identified from title or retained mapping: prediction / prognosis | Not reported; not inferred | Not reported; not inferred | Comparator, validation, performance, and external-validation fields were unavailable in the retained structured extraction and were not inferred. |
| S070 | detection / segmentation; classification / differential diagnosis; prediction / prognosis | Not reported; not inferred | Not reported; not inferred | Task identified from title or retained mapping: detection / segmentation; classification / differential diagnosis; prediction / prognosis | Not reported; not inferred | Not reported; not inferred | Comparator, validation, performance, and external-validation fields were unavailable in the retained structured extraction and were not inferred. |
| S071 | 3D CNN; prediction / prognosis | Not reported; not inferred | Not reported; not inferred | Task identified from title or retained mapping: prediction / prognosis | Not reported; not inferred | Not reported; not inferred | Comparator, validation, performance, and external-validation fields were unavailable in the retained structured extraction and were not inferred. |
| S072 | Autoencoders; prediction / prognosis | Not reported; not inferred | Not reported; not inferred | Task identified from title or retained mapping: prediction / prognosis | Not reported; not inferred | Not reported; not inferred | Comparator, validation, performance, and external-validation fields were unavailable in the retained structured extraction and were not inferred. |
| S073 | Study-specific AI/CAD task | Not reported; not inferred | Not reported; not inferred | Task identified from title or retained mapping: Study-specific AI/CAD task | Not reported; not inferred | Not reported; not inferred | Comparator, validation, performance, and external-validation fields were unavailable in the retained structured extraction and were not inferred. |
| S074 | Interpretable FCN classification | Not reported; not inferred | External AUC | Task identified from title or retained mapping: classification / differential diagnosis | AUC 0.996 internally and 0.876-0.974 across independent cohorts. | Reported in retained structured extraction | Validation and performance fields reproduce the retained structured extraction; no standardized aggregate category was assigned for this revision. |
| S075 | ML infarct segmentation | Not reported; not inferred | Volume agreement | Task identified from title or retained mapping: Study-specific AI/CAD task | Automated infarct extent agreed with diffusion-MRI reference volume. | Not established in retained structured extraction | Validation and performance fields reproduce the retained structured extraction; no standardized aggregate category was assigned for this revision. |
| S076 | Flexible deep differential diagnosis | Not reported; not inferred | Expert and pathology comparison | Task identified from title or retained mapping: Study-specific AI/CAD task | Supported NC, MCI, AD, and non-AD dementia assessment with disease-specific maps. | Not established in retained structured extraction | Validation and performance fields reproduce the retained structured extraction; no standardized aggregate category was assigned for this revision. |
| S077 | reconstruction / synthesis / harmonization | Not reported; not inferred | Not reported; not inferred | Task identified from title or retained mapping: reconstruction / synthesis / harmonization | Not reported; not inferred | Not reported; not inferred | Comparator, validation, performance, and external-validation fields were unavailable in the retained structured extraction and were not inferred. |
| S078 | detection / segmentation | Not reported; not inferred | Not reported; not inferred | Task identified from title or retained mapping: detection / segmentation | Not reported; not inferred | Not reported; not inferred | Comparator, validation, performance, and external-validation fields were unavailable in the retained structured extraction and were not inferred. |
| S079 | 2D CNN / Attention networks; detection / segmentation | Not reported; not inferred | Not reported; not inferred | Task identified from title or retained mapping: detection / segmentation | Not reported; not inferred | Not reported; not inferred | Comparator, validation, performance, and external-validation fields were unavailable in the retained structured extraction and were not inferred. |
| S080 | Tree/ensemble ML; prediction / prognosis | Not reported; not inferred | Not reported; not inferred | Task identified from title or retained mapping: prediction / prognosis | Not reported; not inferred | Not reported; not inferred | Comparator, validation, performance, and external-validation fields were unavailable in the retained structured extraction and were not inferred. |
| S081 | detection / segmentation | Not reported; not inferred | Not reported; not inferred | Task identified from title or retained mapping: detection / segmentation | Not reported; not inferred | Not reported; not inferred | Comparator, validation, performance, and external-validation fields were unavailable in the retained structured extraction and were not inferred. |
| S082 | Study-specific AI/CAD task | Not reported; not inferred | Not reported; not inferred | Task identified from title or retained mapping: Study-specific AI/CAD task | Not reported; not inferred | Not reported; not inferred | Comparator, validation, performance, and external-validation fields were unavailable in the retained structured extraction and were not inferred. |
| S083 | prediction / prognosis | Not reported; not inferred | Not reported; not inferred | Task identified from title or retained mapping: prediction / prognosis | Not reported; not inferred | Not reported; not inferred | Comparator, validation, performance, and external-validation fields were unavailable in the retained structured extraction and were not inferred. |
| S084 | prediction / prognosis | Not reported; not inferred | Not reported; not inferred | Task identified from title or retained mapping: prediction / prognosis | Not reported; not inferred | Not reported; not inferred | Comparator, validation, performance, and external-validation fields were unavailable in the retained structured extraction and were not inferred. |
| S085 | Handcrafted ML; classification / differential diagnosis | Not reported; not inferred | Not reported; not inferred | Task identified from title or retained mapping: classification / differential diagnosis | Not reported; not inferred | Not reported; not inferred | Comparator, validation, performance, and external-validation fields were unavailable in the retained structured extraction and were not inferred. |
| S086 | classification / differential diagnosis | Not reported; not inferred | Not reported; not inferred | Task identified from title or retained mapping: classification / differential diagnosis | Not reported; not inferred | Not reported; not inferred | Comparator, validation, performance, and external-validation fields were unavailable in the retained structured extraction and were not inferred. |
| S087 | Interpretable neural detection | Not reported; not inferred | Held-out/site tests | Task identified from title or retained mapping: detection / segmentation | Gold-standard sensitivity 85%; full withheld cohort sensitivity 59%. | Not established in retained structured extraction | Validation and performance fields reproduce the retained structured extraction; no standardized aggregate category was assigned for this revision. |
| S088 | Handcrafted ML / Tree/ensemble ML; prediction / prognosis | Not reported; not inferred | Not reported; not inferred | Task identified from title or retained mapping: prediction / prognosis | Not reported; not inferred | Not reported; not inferred | Comparator, validation, performance, and external-validation fields were unavailable in the retained structured extraction and were not inferred. |
| S089 | Multimodal / emerging; prediction / prognosis | Not reported; not inferred | Not reported; not inferred | Task identified from title or retained mapping: prediction / prognosis | Not reported; not inferred | Not reported; not inferred | Comparator, validation, performance, and external-validation fields were unavailable in the retained structured extraction and were not inferred. |
| S090 | Study-specific AI/CAD task | Not reported; not inferred | Not reported; not inferred | Task identified from title or retained mapping: Study-specific AI/CAD task | Not reported; not inferred | Not reported; not inferred | Comparator, validation, performance, and external-validation fields were unavailable in the retained structured extraction and were not inferred. |
| S091 | Deep progression prediction | Not reported; not inferred | Longitudinal discrimination | Task identified from title or retained mapping: prediction / prognosis | Modeled disease progression from MRI rather than lesion burden alone. | Not established in retained structured extraction | Validation and performance fields reproduce the retained structured extraction; no standardized aggregate category was assigned for this revision. |
| S092 | Multimodal / emerging; prediction / prognosis | Not reported; not inferred | Not reported; not inferred | Task identified from title or retained mapping: prediction / prognosis | Not reported; not inferred | Not reported; not inferred | Comparator, validation, performance, and external-validation fields were unavailable in the retained structured extraction and were not inferred. |
| S093 | Radiomics fusion | Not reported; not inferred | Train/test AUC | Task identified from title or retained mapping: classification / differential diagnosis | Combined-model test AUC 0.86; multimodal radiomics improved diagnostic context. | Not established in retained structured extraction | Validation and performance fields reproduce the retained structured extraction; no standardized aggregate category was assigned for this revision. |
| S094 | detection / segmentation | Not reported; not inferred | Not reported; not inferred | Task identified from title or retained mapping: detection / segmentation | Not reported; not inferred | Not reported; not inferred | Comparator, validation, performance, and external-validation fields were unavailable in the retained structured extraction and were not inferred. |
| S095 | Multimodal / emerging; classification / differential diagnosis | Not reported; not inferred | Not reported; not inferred | Task identified from title or retained mapping: classification / differential diagnosis | Not reported; not inferred | Not reported; not inferred | Comparator, validation, performance, and external-validation fields were unavailable in the retained structured extraction and were not inferred. |
| S096 | Multimodal / emerging; Study-specific AI/CAD task | Not reported; not inferred | Not reported; not inferred | Task identified from title or retained mapping: Study-specific AI/CAD task | Not reported; not inferred | Not reported; not inferred | Comparator, validation, performance, and external-validation fields were unavailable in the retained structured extraction and were not inferred. |
| S097 | reconstruction / synthesis / harmonization | Not reported; not inferred | Not reported; not inferred | Task identified from title or retained mapping: reconstruction / synthesis / harmonization | Not reported; not inferred | Not reported; not inferred | Comparator, validation, performance, and external-validation fields were unavailable in the retained structured extraction and were not inferred. |
| S098 | Study-specific AI/CAD task | Not reported; not inferred | Not reported; not inferred | Task identified from title or retained mapping: Study-specific AI/CAD task | Not reported; not inferred | Not reported; not inferred | Comparator, validation, performance, and external-validation fields were unavailable in the retained structured extraction and were not inferred. |
| S099 | Tree/ensemble ML; prediction / prognosis | Not reported; not inferred | Not reported; not inferred | Task identified from title or retained mapping: prediction / prognosis | Not reported; not inferred | Not reported; not inferred | Comparator, validation, performance, and external-validation fields were unavailable in the retained structured extraction and were not inferred. |
| S100 | 3D CNN; classification / differential diagnosis | Not reported; not inferred | Not reported; not inferred | Task identified from title or retained mapping: classification / differential diagnosis | Not reported; not inferred | Not reported; not inferred | Comparator, validation, performance, and external-validation fields were unavailable in the retained structured extraction and were not inferred. |
| S101 | classification / differential diagnosis | Not reported; not inferred | Not reported; not inferred | Task identified from title or retained mapping: classification / differential diagnosis | Not reported; not inferred | Not reported; not inferred | Comparator, validation, performance, and external-validation fields were unavailable in the retained structured extraction and were not inferred. |
| S102 | Study-specific AI/CAD task | Not reported; not inferred | Not reported; not inferred | Task identified from title or retained mapping: Study-specific AI/CAD task | Not reported; not inferred | Not reported; not inferred | Comparator, validation, performance, and external-validation fields were unavailable in the retained structured extraction and were not inferred. |
| S103 | Multitask subtype/grade/segmentation | Not reported; not inferred | AUC and Dice | Task identified from title or retained mapping: detection / segmentation | External IDH AUC 0.90, 1p/19q AUC 0.85, grade AUC 0.81, whole-tumor Dice 0.84. | Reported in retained structured extraction | Validation and performance fields reproduce the retained structured extraction; no standardized aggregate category was assigned for this revision. |
| S104 | k-NN / probabilistic ML / Multimodal / emerging; detection / segmentation | Not reported; not inferred | Not reported; not inferred | Task identified from title or retained mapping: detection / segmentation | Not reported; not inferred | Not reported; not inferred | Comparator, validation, performance, and external-validation fields were unavailable in the retained structured extraction and were not inferred. |
| S105 | 2D/3D CNN classification | Not reported; not inferred | Leakage-safe hold-out; accuracy/AUC | Task identified from title or retained mapping: classification / differential diagnosis | 3D variants were similar; 2D was lower; CNN did not consistently exceed SVM. | Not established in retained structured extraction | Validation and performance fields reproduce the retained structured extraction; no standardized aggregate category was assigned for this revision. |
| S106 | Graph neural networks; classification / differential diagnosis | Not reported; not inferred | Not reported; not inferred | Task identified from title or retained mapping: classification / differential diagnosis | Not reported; not inferred | Not reported; not inferred | Comparator, validation, performance, and external-validation fields were unavailable in the retained structured extraction and were not inferred. |
| S107 | 3D U-Net ensemble | Not reported; not inferred | Dice/F1 | Task identified from title or retained mapping: detection / segmentation | MSSEG-1 Dice 0.65 and F1 0.63; released as open-source LST-AI. | Not established in retained structured extraction | Validation and performance fields reproduce the retained structured extraction; no standardized aggregate category was assigned for this revision. |
| S108 | classification / differential diagnosis | Not reported; not inferred | Not reported; not inferred | Task identified from title or retained mapping: classification / differential diagnosis | Not reported; not inferred | Not reported; not inferred | Comparator, validation, performance, and external-validation fields were unavailable in the retained structured extraction and were not inferred. |
| S109 | classification / differential diagnosis; prediction / prognosis | Not reported; not inferred | Not reported; not inferred | Task identified from title or retained mapping: classification / differential diagnosis; prediction / prognosis | Not reported; not inferred | Not reported; not inferred | Comparator, validation, performance, and external-validation fields were unavailable in the retained structured extraction and were not inferred. |
| S110 | Etiological differential diagnosis | Not reported; not inferred | Micro-AUROC | Task identified from title or retained mapping: classification / differential diagnosis | AUROC 0.94 for cognitive state and 0.96 for dementia etiology. | Not established in retained structured extraction | Validation and performance fields reproduce the retained structured extraction; no standardized aggregate category was assigned for this revision. |
| S111 | Self-supervised / transfer; reconstruction / synthesis / harmonization | Not reported; not inferred | Not reported; not inferred | Task identified from title or retained mapping: reconstruction / synthesis / harmonization | Not reported; not inferred | Not reported; not inferred | Comparator, validation, performance, and external-validation fields were unavailable in the retained structured extraction and were not inferred. |
| S112 | Study-specific AI/CAD task | Not reported; not inferred | Not reported; not inferred | Task identified from title or retained mapping: Study-specific AI/CAD task | Not reported; not inferred | Not reported; not inferred | Comparator, validation, performance, and external-validation fields were unavailable in the retained structured extraction and were not inferred. |
| S113 | Support vector machines; classification / differential diagnosis | Not reported; not inferred | Not reported; not inferred | Task identified from title or retained mapping: classification / differential diagnosis | Not reported; not inferred | Not reported; not inferred | Comparator, validation, performance, and external-validation fields were unavailable in the retained structured extraction and were not inferred. |
| S114 | classification / differential diagnosis | Not reported; not inferred | Not reported; not inferred | Task identified from title or retained mapping: classification / differential diagnosis | Not reported; not inferred | Not reported; not inferred | Comparator, validation, performance, and external-validation fields were unavailable in the retained structured extraction and were not inferred. |
| S115 | Study-specific AI/CAD task | Not reported; not inferred | Not reported; not inferred | Task identified from title or retained mapping: Study-specific AI/CAD task | Not reported; not inferred | Not reported; not inferred | Comparator, validation, performance, and external-validation fields were unavailable in the retained structured extraction and were not inferred. |
| S116 | Study-specific AI/CAD task | Not reported; not inferred | Not reported; not inferred | Task identified from title or retained mapping: Study-specific AI/CAD task | Not reported; not inferred | Not reported; not inferred | Comparator, validation, performance, and external-validation fields were unavailable in the retained structured extraction and were not inferred. |
| S117 | U-Net final-infarct prediction | Not reported; not inferred | AUC, Dice, volume error | Task identified from title or retained mapping: prediction / prognosis | Median AUC 0.92 and Dice 0.53 for final lesion prediction. | Not established in retained structured extraction | Validation and performance fields reproduce the retained structured extraction; no standardized aggregate category was assigned for this revision. |
| S118 | Tree/ensemble ML; prediction / prognosis | Not reported; not inferred | Not reported; not inferred | Task identified from title or retained mapping: prediction / prognosis | Not reported; not inferred | Not reported; not inferred | Comparator, validation, performance, and external-validation fields were unavailable in the retained structured extraction and were not inferred. |
| S119 | classification / differential diagnosis | Not reported; not inferred | Not reported; not inferred | Task identified from title or retained mapping: classification / differential diagnosis | Not reported; not inferred | Not reported; not inferred | Comparator, validation, performance, and external-validation fields were unavailable in the retained structured extraction and were not inferred. |
| S120 | Self-supervised / transfer; Study-specific AI/CAD task | Not reported; not inferred | Not reported; not inferred | Task identified from title or retained mapping: Study-specific AI/CAD task | Not reported; not inferred | Not reported; not inferred | Comparator, validation, performance, and external-validation fields were unavailable in the retained structured extraction and were not inferred. |
| S121 | classification / differential diagnosis | Not reported; not inferred | Not reported; not inferred | Task identified from title or retained mapping: classification / differential diagnosis | Not reported; not inferred | Not reported; not inferred | Comparator, validation, performance, and external-validation fields were unavailable in the retained structured extraction and were not inferred. |
| S122 | Attention networks; classification / differential diagnosis | Not reported; not inferred | Not reported; not inferred | Task identified from title or retained mapping: classification / differential diagnosis | Not reported; not inferred | Not reported; not inferred | Comparator, validation, performance, and external-validation fields were unavailable in the retained structured extraction and were not inferred. |
| S123 | Study-specific AI/CAD task | Not reported; not inferred | Not reported; not inferred | Task identified from title or retained mapping: Study-specific AI/CAD task | Not reported; not inferred | Not reported; not inferred | Comparator, validation, performance, and external-validation fields were unavailable in the retained structured extraction and were not inferred. |

## 1.3 Appendix C. Coding dictionary and corrected screening record

**Table S2. Revised coding dictionary**

| **Field** | **Operational definition** | **Rule or allowed value** |
| --- | --- | --- |
| Eligibility decision | Included or contextual/ineligible after record-level re-audit | Decision and reason reported for every prior matrix record in Table S0 |
| Population | Human neurological disease, diagnostic population, or healthy comparator | Disease group, age context, and clinical setting where recoverable |
| Modality | Brain imaging or clinically relevant EEG/MEG source | MRI sequence, fMRI, diffusion, CT, PET/SPECT, EEG, MEG, or multimodal |
| Dataset | Named public resource or institutional/multicenter cohort | Name and site provenance where recoverable |
| Sample size | Participants or examinations in the analytical set | Reported only when recoverable from the retained record |
| Model / task | Principal AI family or task descriptor | May derive from retained mapping or the study title |
| Comparator | Reference standard, clinician, conventional method, or alternative model | NR unless available in the retained structured extraction |
| Validation design | Reported development or evaluation design | Raw recoverable description; no retrospective aggregate category assigned |
| Performance | Reported estimate and evaluation setting | NR unless available in the retained structured extraction |
| External status | Independent cohort, site, geography, or time-period evaluation | Reported, not established, or NR in the retained structured extraction |
| NR | Value absent from the retained structured extraction | Not interpreted as absence from the primary article and not coded as a negative |

**Table S3. Corrected screening-flow and full-text exclusion summary**

| **Screening stage or exclusion reason** | **Count** |
| --- | --- |
| Records identified | 1,078 |
| Duplicates removed | 236 |
| Unique records screened | 842 |
| Title/abstract exclusions | 537 |
| Reports sought | 305 |
| Reports not retrieved | 47 |
| Full texts assessed | 258 |
| No primary brain-imaging focus | 38 |
| No AI/CAD evaluation | 30 |
| Not related to neurological disease | 21 |
| Not an eligible original report | 26 |
| Insufficient technical detail | 9 |
| Duplicate/overlapping analysis | 6 |
| No eligible disease-linked endpoint | 1 |
| Other prespecified reason | 4 |
| Full-text exclusions | 135 |
| Included studies | 123 |

Record-level title/abstract decisions and the itemized list of 135 excluded full texts are retained by the corresponding author and are available on reasonable request. They were not deposited in a public repository for this revision. Table S0 supplies the complete re-audit crosswalk for the 137 records previously presented in the evidence matrix, but it is not a substitute for the original screening log.
